# Sunny with a Chance of Curtailment: Operating the US Grid with Very High Levels of Solar Photovoltaics

**DOI:** 10.1016/j.isci.2019.10.017

**Published:** 2019-10-10

**Authors:** Bethany Frew, Wesley Cole, Paul Denholm, A. Will Frazier, Nina Vincent, Robert Margolis

**Affiliations:** 1 Strategic Energy Analysis Center, National Renewable Energy Laboratory, Golden, CO 80401, USA

**Keywords:** Energy Policy, Energy Engineering, Energy Flexibility

## Abstract

With rapid declines in solar photovoltaic (PV) and energy storage costs, futures with PV penetrations approaching or exceeding 50% of total annual US generation are becoming conceivable. The operational merits of such a national-scale system have not been evaluated sufficiently. Here, we analyze in detail the operational impacts of a future US power system with very high annual levels of PV (>50%) with storage. We show that load and operating reserve requirements can be met for all hours while considering key generator operational constraints. Storage plays an active role in maintaining the balance of supply and demand during sunset hours. Under the highest PV penetration scenario, hours with >90% PV penetration are relatively common, which require rapid transitions between predominately conventional synchronous generation and mostly inverter-based generation. We observe hours with almost 400 GW (over 40%) of economic curtailment and frequent (up to 36%) hours with very low energy prices.

## Highlights

• Load and operating reserves can be met in US grid with up to 55% PV with storage
• Power system must rapidly transition between synchronous and inverter-based generation
• Significant curtailment is seen, with hours of >40% economic curtailment
• Hours with very low energy prices become more frequent, up to 36% of hours

## Graphical Abstract

**Figure:**
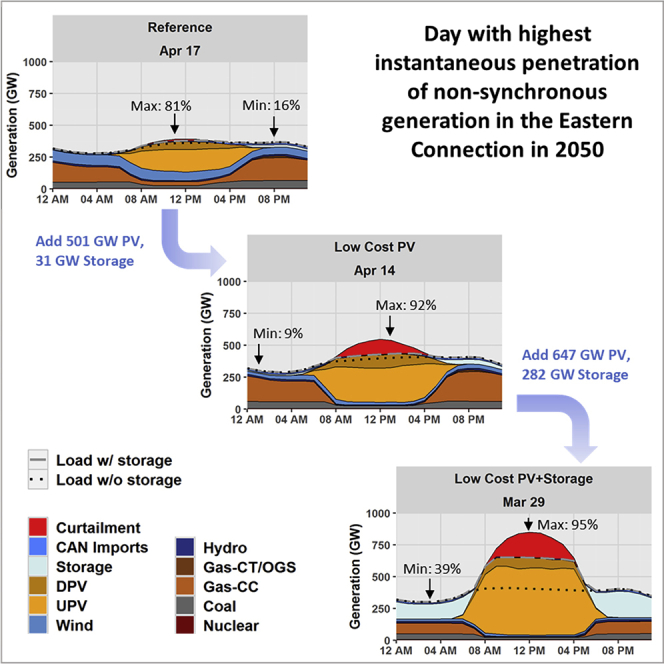

## Introduction

A large and growing body of work has examined the impact of high levels of renewable energy deployment on electric grid planning and operation. A common theme among many of these studies is that a diverse mix of renewable or other low-carbon resources typically produces the lowest system cost (Becker et al., 2014, Frew et al., 2016, Jenkins et al., 2018, MacDonald et al., 2016, Mai et al., 2014). Among the benefits of a diverse resource mix is minimizing the amount of storage required to balance the grid, which has historically been very expensive. However, continued cost declines for solar photovoltaic (PV) plants and aggressively declining energy storage prices suggest that a future with very high penetration levels of PV and storage is plausible and motivate detailed examination of the operational feasibility of such a future (Creutzig et al., 2017, Haegel et al., 2017, Hirth, 2013, Jones-Albertus et al., 2018, Kurtz et al., 2017, Mills and Wiser, 2012, Sivaram and Kann, 2016). The possibility of such a PV-storage dominate future was recently highlighted in the study by Haegel et al. (2019), where the authors “envision a future with ∼10 TW of PV by 2030 and 30 to 70 TW by 2050, providing a majority of global energy.” Of particular importance, the synergies of PV with increasing cost competitive battery storage with durations of 8 h or less are much greater than those with other variable renewable energy (VRE) technologies that do not demonstrate a consistent diurnal pattern. Several of the most highly cited “high renewable penetration” studies that model the contiguous United States have examined scenarios of well under 50% annual PV penetration levels (MacDonald et al., 2016, Mai et al., 2014), whereas studies of very high PV scenarios have had limited spatial extent (Sepulveda et al., 2018) or modeling resolution (Zweibel et al., 2008), resulting in incomplete understanding of the impacts of PV on grid operations and reliability at a national scale. In order to address this gap in the existing literature, we use best-in-class modeling tools with detailed operational treatment to examine a scenario of the full contiguous United States in which PV becomes a predominant source of energy—up to 55% on an annual basis—enabled by sizable deployment of energy storage.

We apply a utility-grade chronologic economic unit commitment and dispatch model of generation and transmission to assess the detailed operation of very high PV systems. We evaluate power system operation under three scenarios of future PV deployment developed by Cole et al. (2018). These scenarios, which are based on outputs from a capacity expansion model, serve as a starting point for this operations-focused study; capacity expansion modeling was not part of the analysis presented here. These three scenarios reflect least-cost system builds in the year 2050 that could result solely from achieving declining cost trajectories for both PV and storage (meaning no additional policy drivers such as renewable portfolio standards or carbon policies). Furthermore, unlike existing US high VRE studies, the system buildout in these scenarios was not prescribed. The “Reference” scenario uses the National Renewable Energy Laboratory (NREL) 2016 Annual Technology Baseline (ATB) (Cole et al., 2016a) mid-level PV cost projections and reaches 17% PV penetration (as a percentage of electricity supplied) in 2050. The “Low-Cost PV” scenario uses the US Department of Energy SunShot 2030 cost targets ($0.03/kilowatt-hour [kWh] for utility-scale PV in 2030 and $0.02/kWh in 2050) and reaches about 32% PV penetration in 2050 (Cole et al., 2018, Cole et al., 2016b). Finally, the “Low-Cost PV + Storage” scenario includes more aggressive battery cost reductions ($97/kWh instead of $220/kWh in the previous two scenarios) and reaches about 55% PV penetration in 2050 (Cole et al., 2018, Cole et al., 2016b). For reference, the total annual solar penetration for the United States in 2017 was about 1.9%, with California leading the nation at 16% (Feldman et al., 2018).

We find that, with appropriate changes to grid operation, 55% PV penetration could be achieved in 2050 while ensuring resource adequacy, addressing net-load variability, and providing sufficient operating reserves. However, typical grid operation with 55% PV would look very different from how the grid operates today. It would include very high instantaneous penetration of nonsynchronous (inverter-based) generators, net-load ramp rates, and curtailment, as well as many hours of zero energy prices. Energy storage can play an active role in maintaining the balance of supply and demand in high PV penetration systems, especially during sunset hours, but it would need to be efficiently scheduled with appropriate foresight of solar and load patterns across large regions. These changes would require new ways of thinking about the role of curtailed energy, along with new market designs and compensation mechanisms for sources of energy that have no variable costs.

**Figure 1:**
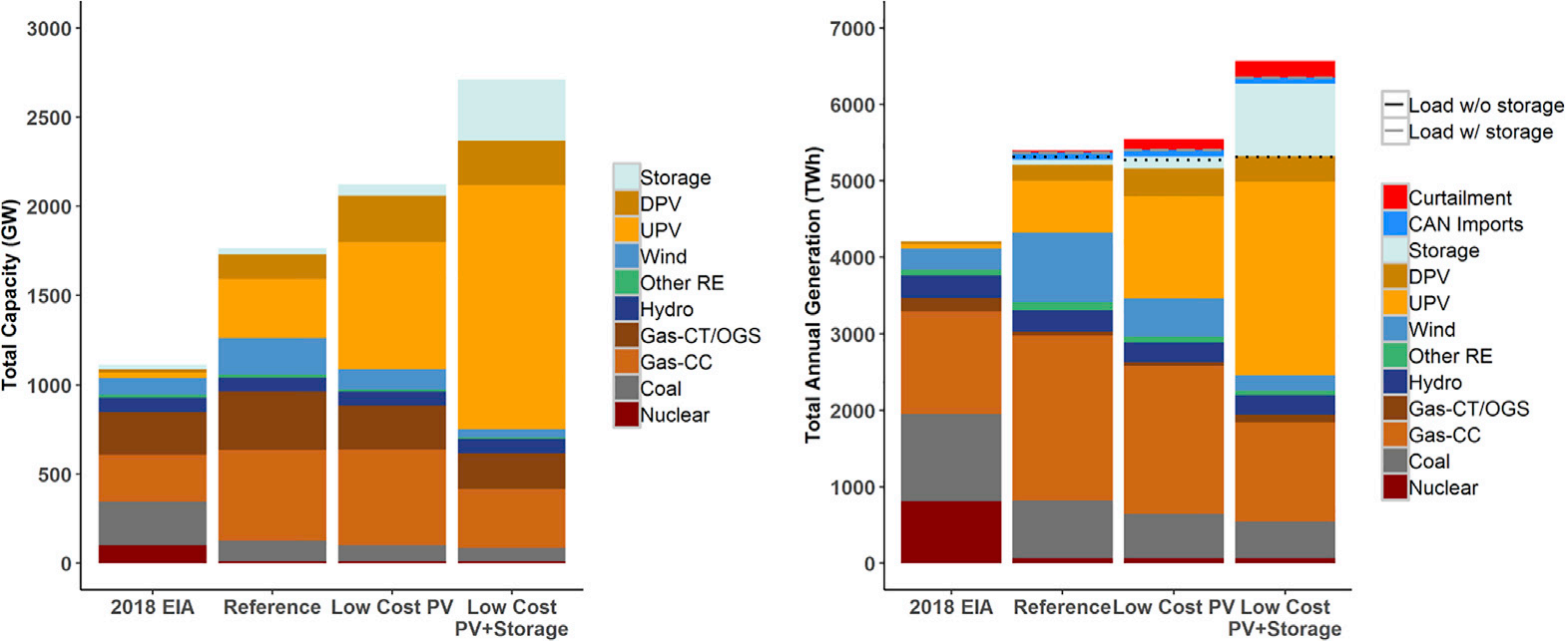
Total Nationwide Installed Capacity (Left) and Annual Generation (Right) in the Actual 2018 System and in 2050 by Scenario Notes: DPV = distributed PV; UPV = utility-scale PV; CAN Imports = net energy imports from Canada; OGS = oil-gas-steam; Storage = battery, pumped hydro, and CAES; Other RE = biopower, geothermal, and concentrated solar power.

We begin with the three scenarios developed in Cole et al. (2018) and illustrated in Figure 1, which shows both nationwide capacity (left) and annual generation (right) by technology type, along with a comparison with the actual 2018 system from EIA data (EIA, 2019). The Reference scenario has 470 GW of PV and 33 GW storage; Low-Cost PV has 971 GW PV and 64 GW storage; and Low-Cost PV + Storage has 1,618 GW PV and 346 GW storage. We then used the commercial production cost model (PCM) software PLEXOS to model hourly system operation for each of these scenarios (Energy Exemplar, 2017). Note that all scenarios and results in this study reflect 2050 only.

## Results

### No Show-Stoppers, but Four Important Deviations from Today's Grid

The PLEXOS production cost modeling results for each scenario were analyzed for unserved load and reserve violations, which are key metrics of grid reliability and resource adequacy. Unserved load, or unmet customer demand, most likely occurs during periods of high demand, such as hot summer afternoons or days with cold weather. Power system planners typically evaluate resource adequacy of systems in detail during these periods (Pfeifenberger et al., 2013). Reserve violations are periods in which there is insufficient spare capacity to meet power plant or transmission line failures or respond to normal random subhourly variations in demand. In addition, we examine PV operations, storage operations, ramping conditions, curtailment, and energy price trends. Collectively, these results provide a view of what a US power system would look like with more than 50% of annual load served by PV-generated electricity.

**Figure 2:**
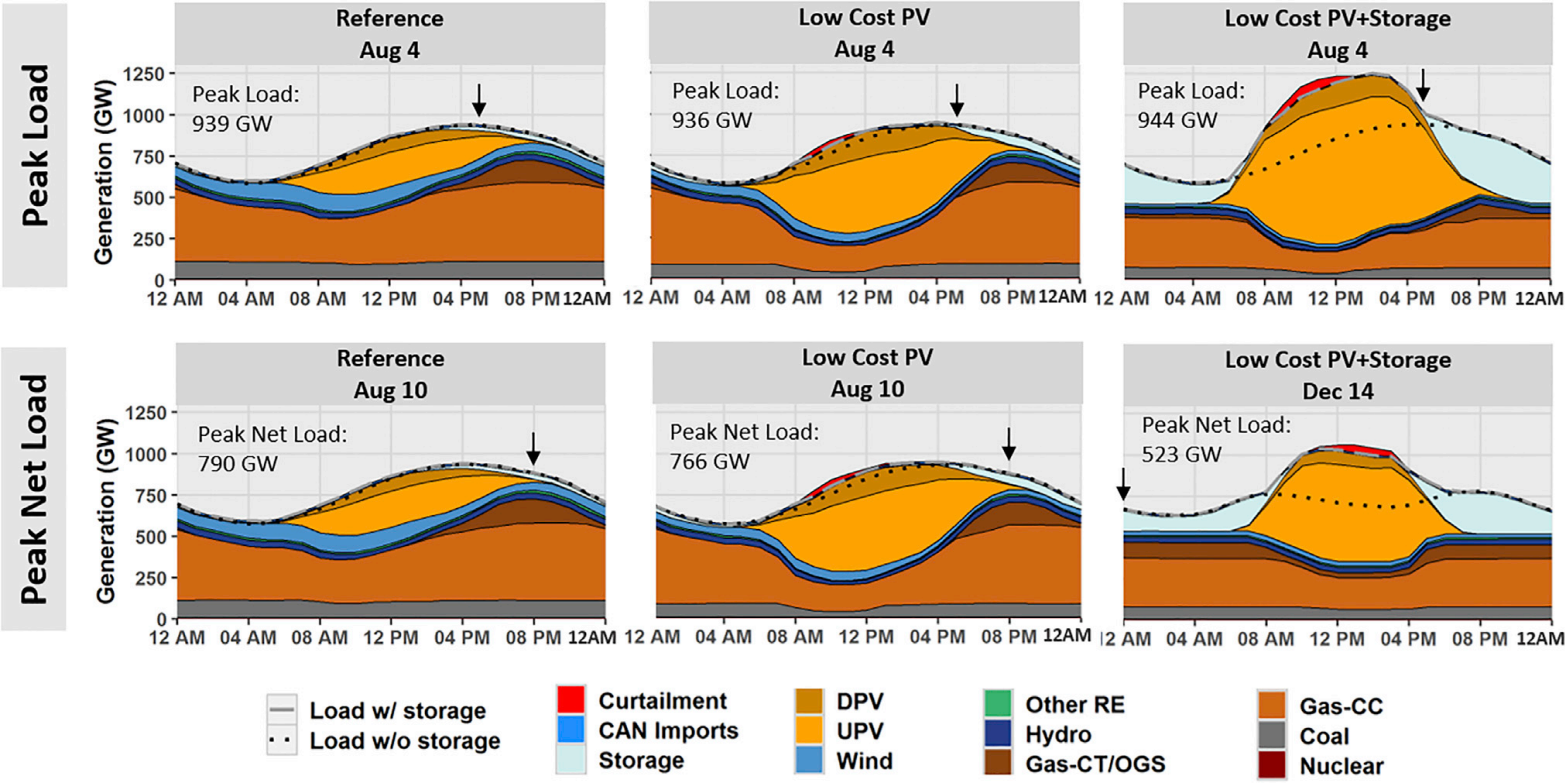
System Dispatch on the Day with Highest Load Hour (Top Row) and Net Load Hour (Bottom Row) for National Systems in 2050 The day of the highest net peak load can be different across scenarios and regions. See also Supplemental Information, Figures S6–S23 for dispatch at RTO region level, as well as Table S5 for the capacity factor and penetration of storage and PV during these hours.

Figure 2 illustrates the dispatch for the day in each scenario with the highest load (without storage) and net load (where net load is the nonstorage load minus the contribution from VRE), which can be different across scenarios and regions. We focus on nationwide results here, but dispatch plots for all RTO regions are included in the Supplemental Information.

The top row of the figure is the day with the peak load hour, whereas the bottom row is the day with the peak net load hour. These hours and their corresponding values are marked in each plot. The dispatch shows the output of each generator, aggregated by class, as well as total system load both with and without storage charging. PV generation is reported as utilized energy (i.e., after curtailment). Total generation will always match load at a national level, but imports and exports may exist at a regional level due to transmission (see Supplemental Information).

Figure 2 illustrates the need for a variety of capacity resources under high PV scenarios to meet peak load. As shown in the top row, the national coincident peak load in 2050 is about 940 GW (+/− depending on the scenario, without storage charging). In the bottom row, the firm capacity contributions from wind and solar reduce this August 4th peak load to an August 10th peak *net* load of 790 GW; in the Reference scenario, this VRE firm capacity contribution results from installed capacities of 470 MW PV and 203 MW wind. In other words, these VRE resources are able to provide some firm system capacity and replace conventional generation capacity. Moving to the bottom middle panel with the Low-Cost PV scenario, PV (971 GW installed capacity) and wind (113 GW installed capacity) are able to further reduce peak net load to 766 GW. However, PV has shifted the net peak load to late afternoon and cannot by itself reduce that load. Moving to the bottom right panel for the Low-Cost PV + Storage scenario, the net peak load is now shifted to the winter. In this scenario, the large contribution of PV and storage (using mostly stored solar energy) provides additional firm capacity, further reducing the need for conventional generation on the peak summer days. This solar- and storage-driven reduction in net load during summer days shifts the hours with highest net load from the summer afternoon to the winter morning, when solar is not producing. A winter peak net load would put more emphasis on winter reliability across many regions of the country. See the Table S5 for a summary of nationwide contributions of PV and storage during peak load and net load hours.

In addition to meeting load during all hours in all locations, the mix of generation resources built in each of the least-cost scenarios was able to maintain three classes of operating reserves: spinning contingency, regulating (frequency regulation), and flexibility reserves. While these results indicate reliable system operation, further examination of the results identifies four major changes that would affect system planning and operation in these future scenarios:

1) Much greater net load ramps and more frequent stops/starts
2) Very high instantaneous penetration of inverter-based generation
3) Significant (but economic) curtailment
4) Greater frequency of low and zero energy prices

### Much Greater Net Load Ramps and More Frequent Stops/Starts

A common theme in studies of wind and solar deployment is increased ramping by the balance of the system (GE Energy, 2010, Lew et al., 2013, Schill et al., 2017). In the scenarios evaluated, we observe even greater need for additional generator ramping, as well as more daily power plant stops and starts.

**Figure 3:**
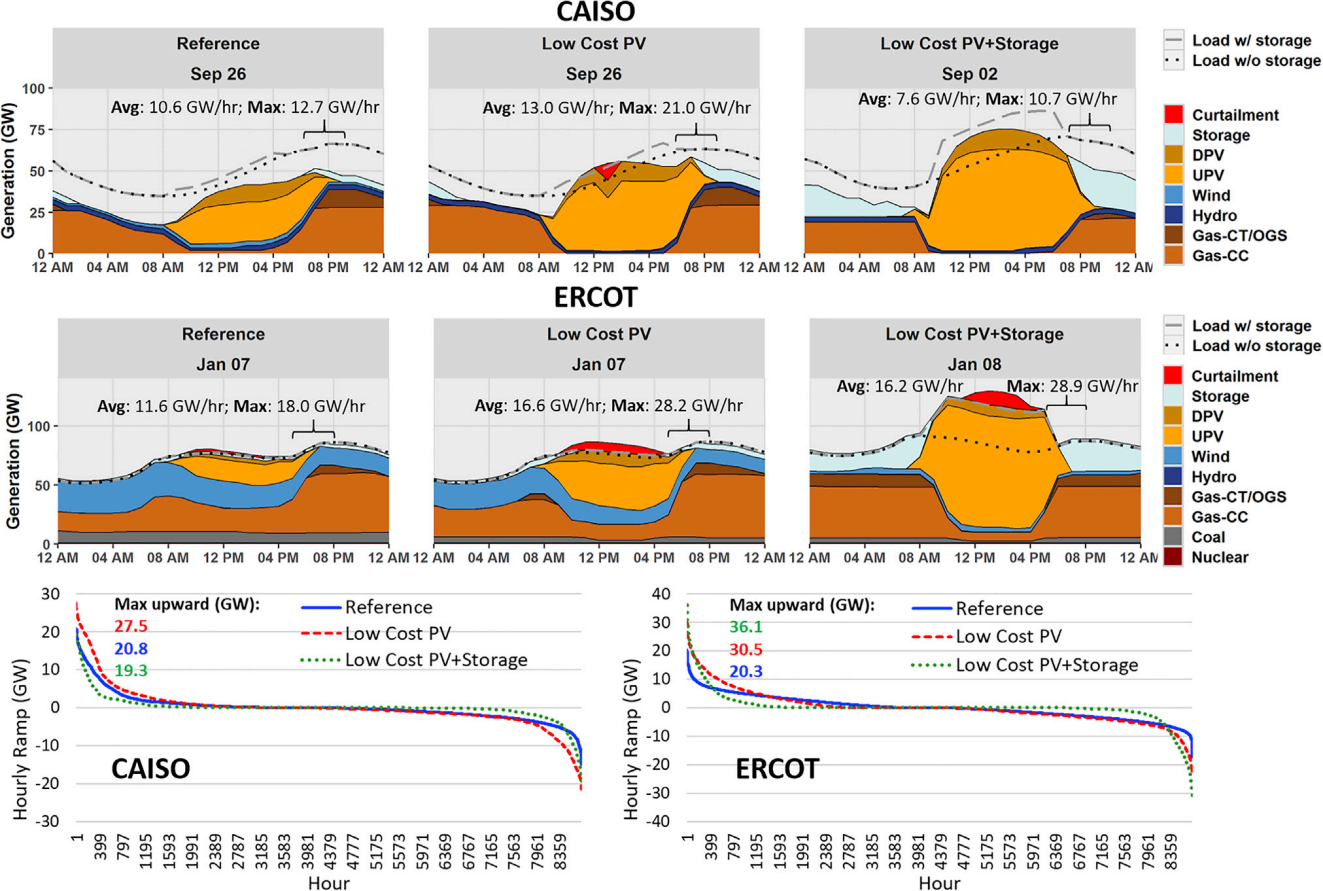
CAISO (First Row) and ERCOT (Second Row) Hourly Dispatch for Each Scenario's Day of Largest Upward 3-h Ramp, and CAISO and ERCOT Hourly Ramp Duration Curve (Third Row), All in 2050 “Avg” is the average hourly ramp across the maximum 3-h period. “Max” is the maximum hourly ramp within the 3-h period. Additional RTO regions, including the days with the largest upward and downward 3-h ramps, are in the Supplemental Information, Figures S24–S41.

The first two rows in Figure 3 provide hourly dispatch stacks for the day with the largest 3-h upward ramp for two regional transmission organization (RTO) regions (see the Figures S24–S41 for dispatch stacks of all 18 RTO regions with both upward and downward 3-h ramps). The PV-dominated CAISO system is shown in the first row of the figure, whereas the mixed PV-wind Electric Reliability Council of Texas (ERCOT) system is shown in the second row. In general, the upward ramps are of greater concern than downward ramps, as ramp-down capabilities are much greater for all plant types and can also be derived from renewables. Renewables are capable of providing rapid upward ramping capacity, but this requires precurtailment, and the value of curtailed energy is typically high during upward ramp events (Denholm et al., 2019). Furthermore, much of the upward ramping requirements in these scenarios is driven by sunset, when solar would not be able to contribute significant upward ramping response.

We also include hourly ramp duration curves (1-h ramps sorted from highest to lowest) for these same regions in the last row of Figure 3; positive values reflect upward ramps, whereas negative values represent downward ramps. In all scenarios for these two regions, the maximum upward ramp occurs during a winter sunset period. Across the scenarios, larger PV levels increase ramp rates, which can be mitigated to varying degrees with energy storage. The Low-Cost PV cases (relative to the Reference scenarios) add significant PV in both regions illustrated, greatly increasing ramp rates (max hourly upward ramps increase by 32% in CAISO and 50% in ERCOT). Adding low-cost storage (the Low-Cost PV + Storage scenario) increases PV in CAISO, but also results in a large amount of storage, which reduces the net ramp rates compared with the Low-Cost PV case (a reduction in max hourly upward ramps of 30%). In other regions (such as ERCOT), the additional PV enabled by low-cost storage increases net ramp rates, resulting in an increase in hourly ramp rates relative to the Low-Cost PV scenario (an increase in max hourly upward ramps of 18%).

Significant ramps have always been part of normal system operation. For example, the current ERCOT power system routinely has 1-h upward ramps that exceed 4,000 megawatts (MW), with occasional ramps of greater than 5,000 MW per hour due to normal load variability (ERCOT 2015-2017). To put this in context of the systems evaluated in this study, if we assume that these ramp rates scale proportionately based solely on the load growth projections used (31% growth in total annual load from 2018 to 5260 TWh in 2050 (EIA, 2016)), this normal load ramping would scale to about 6,500 MW/h in 2050. CAISO has had ramps of similar magnitude and has seen increases in afternoon upward ramps due to increased PV deployment (CAISO, 2018). However, the results in these scenarios show a significant increase in magnitude of upward ramp rate requirements that occur during sunset periods, as shown by the maximum 3-h and 1-h ramp rates in each panel, i.e., upwards of 20–30 GW/h in CAISO and ERCOT (see Figure 3).

The increased ramp rates in these simulations are accomplished by a number of measures, including a more flexible overall mix (with fewer slow-ramping thermal steam units), more frequent starts and stops of combined-cycle (CC) units, greater ramping of online units, and use of fast-ramping energy storage. The use of energy storage, in particular, mitigates some of the ramping challenges. While the ramp rate requirements of these scenarios are within the capabilities of modern generators (see the Table S3 for exact requirements), the almost daily cycling of the CC gas fleet would represent a fundamentally new operating mode for many plants (Lew et al., 2013). A critical factor for achieving these ramping requirements is accurate PV forecasts to ensure CC units can be committed at the appropriate times (Brancucci Martinez-Anido et al., 2016).

### Very High Instantaneous Penetration of Inverter-Based Generation

**Figure 4:**
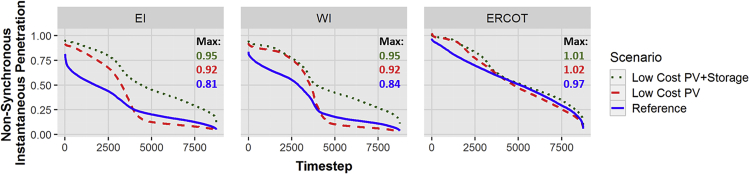
Duration Curves for Instantaneous Penetration of Nonsynchronous Generators in 2050 Notes: EI = Eastern Interconnection, WI = Western Interconnection, ERCOT = Electric Reliability Council of Texas Interconnection.

A second major feature of high PV penetration scenarios is very high instantaneous penetration and associated potential changes in system operation to maintain frequency stability. Figure 4 shows the duration curves of the instantaneous penetration of all nonsynchronous generation (PV, storage, and wind) across the three US interconnections for each of the three scenarios modeled. Instantaneous penetration levels start at a relatively high baseline in the Reference scenario, with maximum values above 80% in all three interconnections. Both the magnitude and frequency of high instantaneous penetration increase moving from the Reference scenario to the Low-Cost PV + Storage scenario. Simultaneously, the number of low instantaneous penetration periods sharply declines, particularly in the Eastern and Western Interconnections (EI and WI). We see instances with nonsynchronous generation meeting all load and in one case (ERCOT in the Low-Cost PV scenario) exceeding 100% due to net exports across direct-current (DC) interface lines. In these periods, nonsynchronous generation must provide all operating reserves, including frequency-responsive reserves needed to maintain frequency stability.

These penetrations are above the levels historically achieved in existing interconnected power systems. ERCOT has reached 56% penetration from wind (ERCOT, 2019), whereas smaller systems outside the three North American Interconnections have achieved higher penetrations of inverter-based generation, including 65% in Ireland (a 6.5 GW peaking system) (EirGrid Group, 2019). System operators and planners are anticipating the transition toward higher levels of VRE (NERC, 2015) and are implementing changes to provide frequency-responsive reserves and voltage control from VRE sources (Kroposki et al., 2017). This includes supplementing traditional inertial and primary frequency response from synchronous generators with nonsynchronous resources that can automatically and rapidly sense and respond to grid frequency; these include demand response, storage, and VRE. For example, since 2012, ERCOT has required new wind generators to have the capability of sensing and responding to system frequency, and in 2018, FERC required new utility-scale wind and PV plants to have frequency responsive capabilities (Asmine et al., 2018, FERC, 2018, Hydro-Quebec, 2009, Matevosyan, 2019). Research has demonstrated that frequency stability can be maintained in both the EI and WI with greatly increased penetration of VRE (Liu et al., 2018, Miller et al., 2015, Tan et al., 2018), although studies have yet to consider 100% instantaneous penetration achieved in the simulations in this work. This will require “grid forming” inverters as opposed to today's grid following inverters that require external sources to provide and maintain a steady reference frequency. Active research is evaluating designs of these devices (Brabandere et al., 2007, Johnson et al., 2016, Seo et al., 2019), as well as considering elements of maintaining system protection (Keller and Kroposki, 2010, Muljadi et al., 2010, Plet and Green, 2014) and other essential reliability services such as black-start (Lopes et al., 2005).

**Figure 5:**
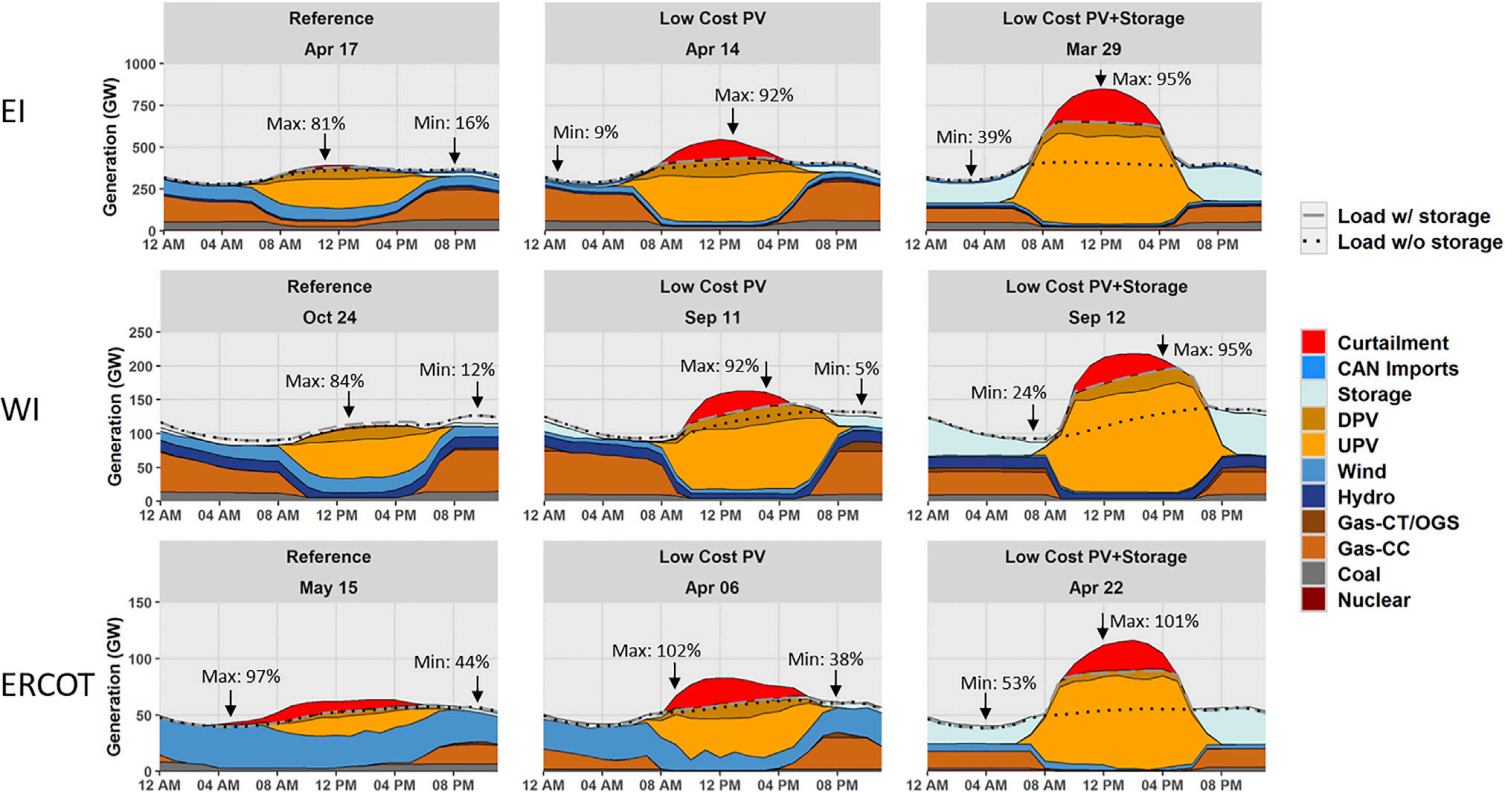
Interconnection-wide Hourly Dispatch during Each Scenario's Day with the Highest Instantaneous Penetration of Nonsynchronous Generation by Interconnection in 2050 Notes: EI = Eastern Interconnection, WI = Western Interconnection, ERCOT = ERCOT Interconnection, DPV = distributed PV, UPV = utility-scale PV, CAN Imports = Canadian net energy imports, OGS = Oil-gas-steam. Arrows show the hour of highest and lowest instantaneous penetration of nonsynchronous generation.

An important feature of a high PV system is the ability to respond to large swings in the amount of synchronous generation online, as shown by the examples in Figure 5. These hourly dispatch plots capture the day with the maximum instantaneous penetration of nonsynchronous generators for each interconnection. Each plot shows the fraction of load met by nonsynchronous generation at the hours with highest and lowest instantaneous penetration. The system moves from having significant operation of synchronous generation to almost none and back again over the span of several hours, meaning that these systems will need to continue maintaining conventional generator stability (such as generator synchronism and rotor angle stability) (Kundur, 1994) and issues related to 100% inverter-based grids (such as very low inherent inertial response) and all conditions in-between.

### Significant (but Economic) Curtailment

Energy is curtailed when generation exceeds demand and/or when system conditions impose operational constraints that preclude all of the available energy from being utilized (Bird et al., 2016, Bird et al., 2014), such as minimum generation levels from conventional plants (Bistline, 2018, Denholm et al., 2018). The ability to curtail output from VRE sources is an important element of maintaining supply/demand balance and system frequency.

Large amounts of total and instantaneous curtailment are seen in our scenarios, particularly in the higher PV cases (see Table 1). Not surprisingly, and consistent with previous literature (Bird et al., 2016, Golden and Paulos, 2015, Hitaj, 2015), PV curtailment is highest during the spring and fall, and the regions with the most curtailment tend to be those with the greatest PV generation (see the Figure S4 illustrating regional curtailment patterns).

**Table 1:** Nationwide 2050 Curtailment Summary Statistics

	Total Annual Curtailment (% of Potential)	Max Instantaneous Curtailment (GW, % of Potential)	Percent of Hours with Curtailed Energy
Reference	1.5%	43.1 GW, 14.0%	1.7%
Low-Cost PV	6.1%	278 GW, 58.6%	5.3%
Low-Cost PV + Storage	6.6%	394 GW, 41.1%	4.9%

See also Figures S4 and S5.

The very large quantity of instantaneous curtailment reflects a possible fundamental paradigm shift in how systems will need to operate in high solar futures, that is, the idea that curtailment may be a “new normal” for everyday operation of the power system under high VRE—namely wind and PV—penetrations (E3, 2014). This idea is supported by recent changes in utility planning and operations activities that incorporate a certain curtailment, or availability, level of VRE resources (APS, 2017, CAISO, 2019). Curtailed energy is increasingly seen as a source of operational flexibility, including as a potential source for operating reserves (Nelson et al., 2018). Given the PV cost reductions assumed in these scenarios, curtailment does not preclude economic deployment of solar. In fact, total PV production exceeds load plus storage for many hours of the year (see, e.g., Figure 5). While instantaneous curtailment can reach over 58%, the annual average is just over 6.6% for the highest penetration case. For context, this result implies that PV curtailment *increases* the effective levelized cost of energy (LCOE) of PV by about 7%. However, given the low initial assumed cost (achieving $0.02/kWh by 2050 before curtailment), it is still less expensive than the variable costs of operating most thermal generators. This annual curtailment value is consistent with recent ultra-high, low-cost VRE scenarios across multiple US regions when firm non-VRE capacity is available (Sepulveda et al., 2018).

### Greater Frequency of Low and Zero Energy Prices

Currently, approximately 70% of annual US electricity demand is met in regions with restructured wholesale markets. These locations have markets for energy and a variety of operating reserves where the prices (and generator payments) are set by the variable costs of the “marginal” generator (FERC, 2015). Similar markets exist in many regions around the world. An important outstanding question in electricity market design is the possible need for changes due to increasing deployment of technologies that have zero variable cost and can thus set the market price of energy (or reserves) to zero. Production cost models such as PLEXOS report prices for energy and each reserve product in a manner similar to the market management software used by system operators and can therefore provide a general idea of what market conditions might look like under high PV penetration scenarios.

For each scenario, a single time series of hourly energy market marginal prices was produced by PLEXOS for each of the 134 PLEXOS regions (see the Figure S2 for a map defining these regions). Key trends reveal that as more PV is added to the system, energy prices decline, energy price variability increases, and the frequency of hours with zero prices increases.

**Figure 6:**
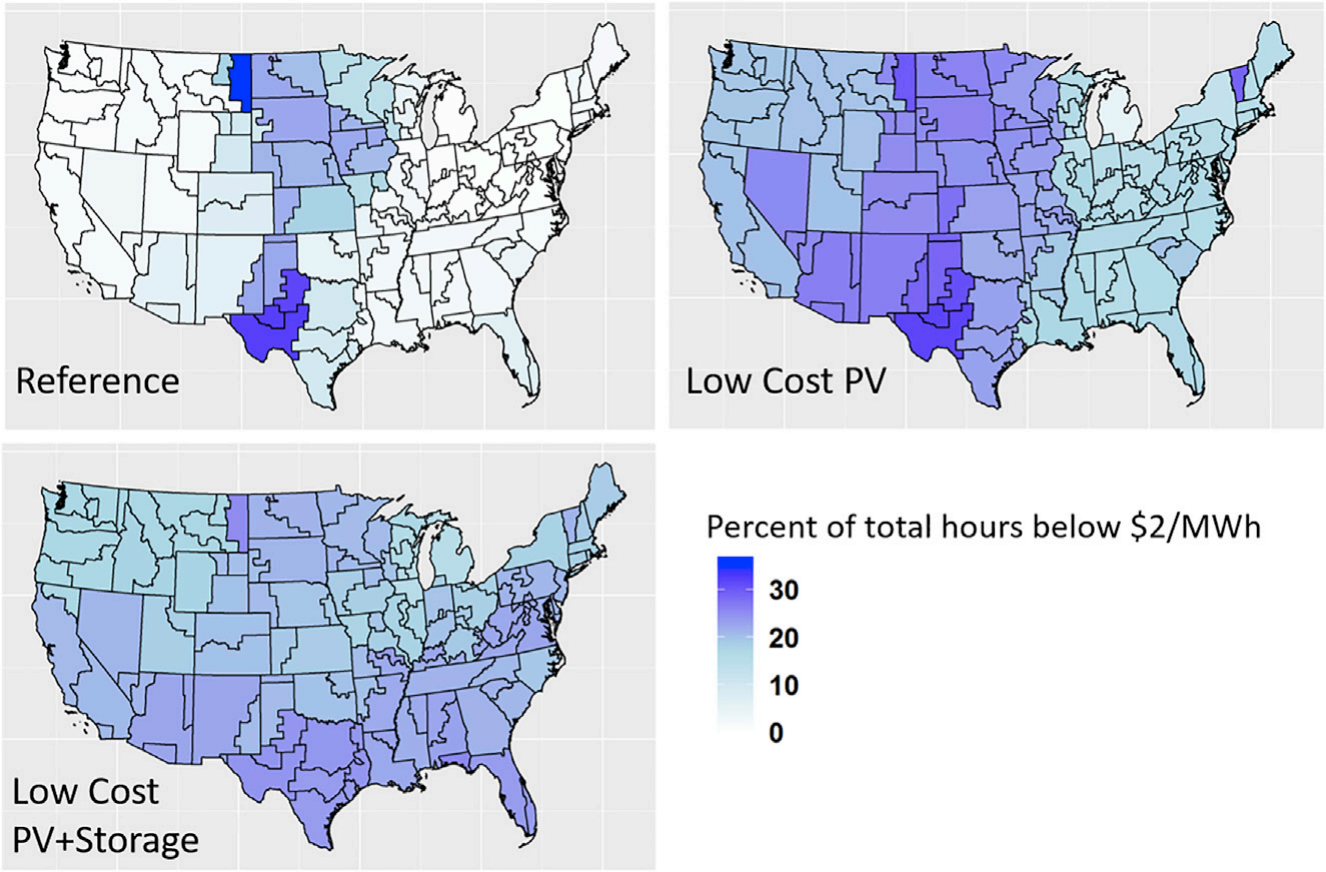
Frequency of Hours with Regional Energy Prices below the Variable Operation Cost of Battery Storage by the 134 Modeled Regions for the Model Year 2050

A spatial breakdown of the frequency of near-zero-price (less than $2 per Megawatt-hour [MWh]) hours across the full year by region is summarized in Figure 6. The assumed variable operation cost for battery storage units is less than $2/MWh; thus, this was used as the near-zero-price threshold. The Reference case sees a relatively high frequency of near-zero-price hours in the wind-dominated central region of the United States next to interconnection interfaces where transmission constraints drive price differences. As more PV is deployed in the Low-Cost PV and Low-Cost PV + Storage scenarios, the frequency of near-zero-price hours becomes more uniformly distributed, with a smaller magnitude in any individual region.

Near-zero-price events can occur when any zero-marginal-cost resource is generating, including PV or wind or pumped hydro storage. However, the majority of events in this case are driven by PV curtailment, with 85%–95% of all zero-price hours occurring during VRE curtailment events. As a result, the number of zero-price hours scales similarly to the number of curtailment hours seen previously in Table 1, resulting in a more than tripling of the number of zero-price hours between the Reference case and the Low-Cost PV and Low-Cost PV + Storage scenarios. Simultaneously, the load-weighted average energy price also declines as more PV is added to the system, driven in part by the increasing number of zero-price hours. In addition to ensuring revenue sufficiency in markets with large amounts of zero marginal prices, markets will likely require careful scheduling of energy storage considering system-wide load patterns.

## Discussion

Using a linked capacity expansion and production cost modeling approach, we examined the economic and technical feasibility of achieving very high penetrations of PV on the US power system. Simulations reveal the changes to operation that may be required to allow over 90% instantaneous penetration levels of PV alone, while accounting for various operational considerations, including unit commitment, startup costs, ramp rates, minimum generation levels, and transmission constraints.

In possible futures with these high levels of PV, storage can effectively help maintain balance of supply and demand, especially during critical sunset hours. In our results, we see a significant increase in the charging and discharging quantities as more PV is added to the system. Discharging is particularly beneficial during sunset transition periods when the system is ramping thermal units back up after startup conditions or operating at minimum generation. As storage scheduling and dispatch may become more important in futures with high levels of PV, so do market design and operational rules, as these guide how storage resources are signaled and compensated for operation.

Even with high levels of storage, we see significant curtailment during many spring days. This may require a fundamental shift in the view of curtailment as a mechanism—and not a barrier—to integrating large amounts of VRE resources. Future work should consider the value of utilizing this otherwise-curtailed energy, including the role of price-responsive demand, controlled electric vehicle charging, and new industrial processes (Hale et al., 2018a, Hale et al., 2018b, Jain et al., 2019, Jonghe et al., 2014, Mai et al., 2018, Ruth et al., 2019, Stoll et al., 2017).

Finally, we see that as more PV is added to the system, energy prices decline and the frequency of hours with zero prices increases. This again points to the need to consider the broader market assumptions and implications in possible futures with high PV penetrations. Of particular interest is the so called missing money (or revenue sufficiency) problem, where markets do not provide sufficient opportunity for generators that are needed for reliability to recover both their fixed and variable costs (Hogan, 2017). The concern is that low electricity prices—collectively across the suite of energy, capacity (where is exists), and ancillary service products—do not reflect the true long-run value of electricity and thereby undermine reliability. While a wide range of market-based flaws can contribute to this problem, price suppression effects from zero-marginal-cost VRE resources are well documented in theory and in practice (Cramton et al., 2013, Frew et al., 2019, Levin and Botterud, 2015, Seel et al., 2018, Wiser et al., 2017). In futures with very high PV penetrations, it is critical that areas with competitive wholesale electricity markets have efficient price signals that align with the full set of physical system requirements.

### Limitations of the Study

While this study demonstrates that PV and storage can meet US power system needs at very high penetrations, results only reflect select elements of operational feasibility. Future efforts should account for the impact and costs of additional physical constraints such as frequency stability and system protection, as well as institutional aspects such as market design that can impact the economics and therefore the operational outcomes. This includes the ongoing need to maintain and compensate low capacity-factor peaking resources or capacity needed for frequent cycling in response to increased variability.

Additionally, while the modeling results presented here reflect many highly resolved and detailed components, the study is limited by some data and computational constraints. These include using one year of time-synchronous load and VRE data (instead of multiple years that reflect varying weather outcomes) and capturing load growth on a total annual basis (instead of reflecting changes to the load shape over time that might occur due to technology changes such as electric vehicles, demand response, energy efficiency measures, or climate change). Future work could improve upon these elements.

## Methods

All methods can be found in the accompanying Transparent Methods supplemental file.

## Supplemental Information

Document S1. Transparent Methods, Figures S1–S41, and Tables S1–S5

## References

[bib1] APS (2017). 2017 Integrated Resource Plan.

[bib2] Asmine M., Langlois C., Aubut N. (2018). Inertial response from wind power plants during a frequency disturbance on the Hydro-Quebec system – event analysis and validation. IET Renew. Power Gener..

[bib3] Becker S., Frew B.A., Andresen G.B., Zeyer T., Schramm S., Greiner M., Jacobson M.Z. (2014). Features of a fully renewable US electricity system: optimized mixes of wind and solar PV and transmission grid extensions. Energy.

[bib4] Bird L., Cochran J., Wang X. (2014). Wind and Solar Energy Curtailment: Experience and Practices in the United States (No. NREL/TP-6A20-60983).

[bib5] Bird L., Lew D., Milligan M., Carlini E.M., Estanqueiro A., Flynn D., Gomez-Lazaro E., Holttinen H., Menemenlis N., Orths A. (2016). Wind and solar energy curtailment: a review of international experience. Renew. Sustain. Energy Rev..

[bib6] Bistline J. (2018). Turn down for what? the economic value of operational flexibility in electricity markets. IEEE Trans. Power Syst..

[bib7] Brabandere K.D., Bolsens B., den Keybus J.V., Woyte A., Driesen J., Belmans R. (2007). A voltage and frequency droop control method for parallel inverters. IEEE Trans. Power Electron..

[bib8] Brancucci Martinez-Anido C., Botor B., Florita A.R., Draxl C., Lu S., Hamann H.F., Hodge B.-M. (2016). The value of day-ahead solar power forecasting improvement. Sol. Energy.

[bib9] CAISO (2018). Historical EMS Hourly Load (2007-2017). http://www.caiso.com/planning/Pages/ReliabilityRequirements/Default.aspx#Historical.

[bib10] CAISO (2019). Managing Oversupply. http://www.caiso.com/informed/Pages/ManagingOversupply.aspx#dailyCurtailment.

[bib11] Cole W., Kurup P., Hand M., Feldman D., Sigrin B., Lantz E., Stehly T., Augustine C., Turchi C., O’Connor P., Waldoch C. (2016). 2016 Annual Technology Baseline (No. NREL/PR-6A20-66944).

[bib12] Cole, W.J., Marcy, C., Krishnan, V.K., Margolis, R. (2016b). Utility-scale lithium-ion storage cost projections for use in capacity expansion models, In: 2016 North American Power Symposium (NAPS). Presented at the 2016 North American Power Symposium (NAPS), pp. 1–6. 10.1109/NAPS.2016.7747866.

[bib13] Cole W., Frew B., Gagnon P., Reimers A., Zuboy J., Margolis R. (2018). Envisioning a low-cost solar future: Exploring the potential impact of Achieving the SunShot 2030 targets for photovoltaics. Energy Oxf..

[bib14] Cramton P., Ockenfels A., Stoft S. (2013). Capacity market fundamentals. Econ. Energy Environ. Policy.

[bib15] Creutzig F., Agoston P., Goldschmidt J.C., Luderer G., Nemet G., Pietzcker R.C. (2017). The underestimated potential of solar energy to mitigate climate change. Nat. Energy.

[bib16] Denholm P., Brinkman G., Mai T. (2018). How low can you go? The importance of quantifying minimum generation levels for renewable integration. Energy Policy.

[bib17] Denholm P.L., Sun Y., Mai T.T. (2019). An Introduction to Grid Services: Concepts, Technical Requirements, and Provision from Wind (No. NREL/TP-6A20–72578).

[bib18] E3 (2014). Investigating a Higher Renewables Portfolio Standard in California..

[bib19] EIA (2016). Annual Energy Outlook 2016 (No. DOE/EIA-0383(2016)).

[bib20] EIA (2019). Electric Power Monthly.

[bib21] EirGrid Group (2019). Renewable Energy [WWW Document]. http://www.eirgridgroup.com/how-the-grid-works/renewables/.

[bib22] Energy Exemplar, (2017). PLEXOS Simulation Software. https://energyexemplar.com/products/plexos-simulation-software/.

[bib23] ERCOT (2012-2017). Hourly Load Data Archives. http://www.ercot.com/gridinfo/load/load_hist/.

[bib24] ERCOT (2019). Wind Integration Report [WWW Document]. http://mis.ercot.com/misapp/GetReports.do?reportTypeId=13105&reportTitle=Wind%20Integration%20Reports%20&showHTMLView=&mimicKey.

[bib25] Feldman D.J., Margolis R.M., Hoskins J. (2018). Q4 2017/Q1 2018 Solar Industry Update (No. NREL/PR-6A20–71493).

[bib26] FERC (2015). Energy Primer: A Handbook of Energy Market Basics.

[bib27] FERC (2018). Order No. 842: Essential Reliability Services and the Evolving Bulk-Power System—Primary Frequency Response, Issued February 15, 2018. https://cdn.misoenergy.org/2018-02-15%20162%20FERC%20%C2%B6%2061,128%20Docket%20No.%20RM16-6-000133298.pdf.

[bib28] Frew B.A., Becker S., Dvorak M.J., Andresen G.B., Jacobson M.Z. (2016). Flexibility mechanisms and pathways to a highly renewable US electricity future. Energy.

[bib29] Frew B., Stephen G., Sigler D., Lau J., Jones W., Bloom A. (2019). forthcoming. Evaluating resource adequacy impacts on energy market prices across wind and solar penetration levels. Electr. J..

[bib30] GE Energy (2010). Western Wind and Solar Integration Study: Phase 1 (No. NREL/SR-550–47434).

[bib31] Golden R., Paulos B. (2015). Curtailment of renewable energy in California and beyond. Electr. J..

[bib32] Haegel N.M., Margolis R., Buonassisi T., Feldman D., Froitzheim A., Garabedian R., Green M., Glunz S., Henning H.-M., Holder B. (2017). Terawatt-scale photovoltaics: trajectories and challenges. Science.

[bib33] Haegel N.M., Atwater H., Barnes T., Breyer C., Burrell A., Chiang Y.-M., Wolf S.D., Dimmler B., Feldman D., Glunz S. (2019). Terawatt-scale photovoltaics: Transform global energy. Science.

[bib34] Hale E.T., Bird L.A., Padmanabhan R., Volpi C.M. (2018). Potential Roles for Demand Response in High-Growth Electric Systems with Increasing Shares of Renewable Generation (No. NREL/TP-6A20–70630).

[bib35] Hale E.T., Stoll B.L., Novacheck J.E. (2018). Integrating solar into Florida’s power system: potential roles for flexibility. Sol. Energy.

[bib36] Hirth L. (2013). The market value of variable renewables: the effect of solar wind power variability on their relative price. Energy Econ..

[bib37] Hitaj C. (2015). Location matters: the impact of renewable power on transmission congestion and emissions. Energy Policy.

[bib38] Hogan M. (2017). Follow the missing money: ensuring reliability at least cost to consumers in the transition to a low-carbon power system. Electr. J..

[bib39] Hydro-Quebec (2009). Transmission Provider Technical Requirements for the Connection of Power Plants to the Hydro-Quebec Transmission System. http://www.hydroquebec.com/transenergie/fr/commerce/pdf/exigence_raccordement_fev_09_en.pdf.

[bib40] Jain H., Palmintier B.S., Krishnamurthy D., Krad I., Hale E.T. (2019). Evaluating the Impact of Price-Responsive Load on Power Systems Using Integrated T&D Simulation: Preprint (No. NREL/CP-5D00–70197).

[bib41] Jenkins J.D., Luke M., Thernstrom S. (2018). Getting to zero carbon emissions in the electric power sector. Joule.

[bib42] Johnson B.B., Sinha M., Ainsworth N.G., Dörfler F., Dhople S.V. (2016). Synthesizing virtual oscillators to control islanded inverters. IEEE Trans. Power Electron..

[bib43] Jones-Albertus R., Cole W., Denholm P., Feldman D., Woodhouse M., Margolis R. (2018). Solar on the rise: how cost declines and grid integration shape solar’s growth potential in the United States. MRS Energy Sustain..

[bib44] Jonghe C.D., Hobbs B.F., Belmans R. (2014). Value of price responsive load for wind integration in unit commitment. IEEE Trans. Power Syst..

[bib45] Keller J., Kroposki B. (2010). Understanding Fault Characteristics of Inverter-Based Distributed Energy Resources (No. NREL/TP-550-46698).

[bib46] Kroposki B., Johnson B., Zhang Y., Gevorgian V., Denholm P., Hodge B.M., Hannegan B. (2017). Achieving a 100% renewable grid: operating electric power systems with extremely high levels of variable renewable energy. IEEE Power Energy Mag..

[bib47] Kundur P. (1994). Power System Stability and Control.

[bib48] Kurtz S., Haegel N., Sinton R., Margolis R. (2017). A new era for solar [WWW Document]. Nat. Photon..

[bib49] Levin T., Botterud A. (2015). Electricity market design for generator revenue sufficiency with increased variable generation. Energy Policy.

[bib50] Lew D., Brinkman G., Ibanez E., Hodge B.M., Hummon M., Florita A., Heaney M. (2013). The Western Wind and Solar Integration Study Phase 2 (No. NREL/TP-5500–55588).

[bib51] Liu Y., You S., Tan J., Zhang Y., Liu Y. (2018). Frequency response assessment and enhancement of the U.S. power grids toward extra-high photovoltaic generation penetrations—an industry perspective. IEEE Trans. Power Syst..

[bib52] Lopes, J.A.P., Moreira, C.L., Madureira, A.G., Resende, F.O., Wu, X., Jayawarna, N., Zhang, Y., Jenkins, N., Kanellos, F., Hatziargyriou, N. (2005). Control strategies for microgrids emergency operation, In: 2005 International Conference on Future Power Systems. Presented at the 2005 International Conference on Future Power Systems, pp. 6. 10.1109/FPS.2005.204226.

[bib53] MacDonald A.E., Clack C.T.M., Alexander A., Dunbar A., Wilczak J., Xie Y. (2016). Future cost-competitive electricity systems and their impact on US CO_2_ emissions. Nat. Clim. Change.

[bib54] Mai T., Mulcahy D., Hand M.M., Baldwin S.F. (2014). Envisioning a renewable electricity future for the United States. Energy.

[bib55] Mai T.T., Jadun P., Logan J.S., McMillan C.A., Muratori M., Steinberg D.C., Vimmerstedt L.J., Haley B., Jones R., Nelson B. (2018). Electrification Futures Study: Scenarios of Electric Technology Adoption and Power Consumption for the United States (No. NREL/TP-6A20-71500).

[bib56] Matevosyan, J. (2019). Evolution of ERCOT’s Frequency Control and Ancillary Services for Higher Levels of Inverter-Based Generation. https://www.esig.energy/resources/evolution-of-ercots-frequency-control-and-ancillary-services-for-higher-levels-of-inverter-based-generation-julia-matevosyan-february-2019/.

[bib57] Miller, N.W., Shao, M., D’aquila, R., Pajic, S., Clark, K. (2015). Frequency Response of the US Eastern Interconnection Under Conditions of High Wind and Solar Generation, In: 2015 Seventh Annual IEEE Green Technologies Conference. Presented at the 2015 Seventh Annual IEEE Green Technologies Conference, pp. 21–28. 10.1109/GREENTECH.2015.31.

[bib58] Mills, A.D., Wiser, R.H. (2012). Changes in the economic value of photovoltaic generation at high penetration levels: A pilot case study of California, in: 2012 IEEE 38th Photovoltaic Specialists Conference (PVSC) PART 2. Presented at the 2012 IEEE 38th Photovoltaic Specialists Conference (PVSC) PART 2, pp. 1–9. 10.1109/PVSC-Vol2.2012.6656763.

[bib59] Muljadi E., Samaan N.A., Gevorgian V., Li J., Pasupulati S. (2010). Short Circuit Current Contribution for Different Wind Turbine Generator Types (No. PNNL-SA-70076).

[bib60] Nelson J., Kasina S., Stevens J., Moore J., Olson A., Morjaria M., Smolenski J., Aponte J. (2018). Investigating the Economic Value of Flexible Solar Power Plant Operation.

[bib61] NERC (2015). Integration of Variable Generation Task Force: Summary and Recommendations of 12 Tasks. Atlanta, GA. https://www.nerc.com/comm/PC/Integration%20of%20Variable%20Generation%20Task%20Force%20I1/IVGTF%20Summary%20and%20Recommendation%20Report_Final.pdf.

[bib62] Pfeifenberger J.P., Spees K., Carden K., Wintermantel N. (2013). Resource Adequacy Requirements: Reliability and Economic Implications.

[bib63] Plet C.A., Green T.C. (2014). Fault response of inverter interfaced distributed generators in grid-connected applications. Electr. Power Syst. Res..

[bib64] Ruth, M., Jadun, P., Elgowainy, A. (2019). H2@Scale Analysis. Presented at DOE Hydrogen and Fuel Cells Program 2019 Annual Merit Review and Peer Evaluation Meeting. https://www.nrel.gov/docs/fy19osti/73559.pdf.

[bib65] Schill W.-P., Pahle M., Gambardella C. (2017). Start-up costs of thermal power plants in markets with increasing shares of variable renewable generation. Nat. Energy.

[bib66] Seel J., Mills A.D., Wiser R.H., Deb S., Asokkumar A., Hassanzadeh M., Aarabali A. (2018). Impacts of High Variable Renewable Energy Futures on Wholesale Electricity Prices, and on Electric-Sector Decision Making (No. LBNL-2001163).

[bib67] Seo, G., Colombino, M., Subotic, I., Johnson, B., Groß, D., Dörfler, F. (2019). Dispatchable Virtual Oscillator Control for Decentralized Inverter-dominated Power Systems: Analysis and Experiments, In: 2019 IEEE Applied Power Electronics Conference and Exposition (APEC). Presented at the 2019 IEEE Applied Power Electronics Conference and Exposition (APEC), pp. 561–566. 10.1109/APEC.2019.8722028.

[bib68] Sepulveda N.A., Jenkins J.D., de Sisternes F.J., Lester R.K. (2018). The role of firm low-carbon electricity resources in deep decarbonization of power generation. Joule.

[bib69] Sivaram V., Kann S. (2016). Solar power needs a more ambitious cost target [WWW Document]. Nat. Energy.

[bib70] Stoll, B., Buechler, E., Hale, E. (2017). The value of demand response in Florida. Electr. J., Energy Policy Institute’s Seventh Annual Energy Policy Research Conference 30, 57–64.

[bib71] Tan, J., Zhang, Y., You, S., Liu, Y., Liu, Y. (2018). Frequency Response Study of U.S. Western Interconnection under Extra-High Photovoltaic Generation Penetrations, In: 2018 IEEE Power Energy Society General Meeting (PESGM). Presented at the 2018 IEEE Power Energy Society General Meeting (PESGM), pp. 1–5. 10.1109/PESGM.2018.8586163.

[bib72] Wiser R.H., Mills A., Seel J., Levin T., Botterud A. (2017). Impacts of Variable Renewable Energy on Bulk Power System Assets, Pricing, and Costs (No. LBNL-2001082).

[bib73] Zweibel K., Mason J., Fthenakis V. (2008). A solar grand plan. Sci. Am..

