# Supplementary material for: Sunny with a Chance of Curtailment: Operating the US Grid with Very High Levels of Solar Photovoltaics
Source: iScience. 2019 Oct 10;21:436–47. doi: 10.1016/j.isci.2019.10.017 (PMC6849084; doi:10.1016/j.isci.2019.10.017)
Supplement: Document S1. Transparent Methods, Figures S1–S41, and Tables S1–S5 [file mmc1.docx]

# Transparent Methods

## Analytical Approach

Several recent analyses have evaluated the buildout of highly renewable U.S. power systems, including several “100%” renewable energy studies. Those studies often assume a predetermined renewable penetration target and then determine the corresponding least-cost system buildout accounting for a simplified representation of operations (Becker et al., 2015, 2014; Bussar et al., 2014; Connolly et al., 2016; Frew et al., 2016; Mathiesen et al., 2011). This study differs in two primary ways. First, the scenarios were developed using a more bottom-up approach: assumed technology cost trajectories were input to a capacity expansion model to yield the least-cost penetration level of PV and storage. No fixed penetration target was set, and there was no carbon constraint. Second, the subsequent use of a production cost model allowed for a more detailed representation of system operations, including unit commitment and dispatch of individual generators, chronological tracking of storage, ramping and minimum generation constraints, and transmission power flow across 134 regions. The low-cost PV scenarios here result in very high PV penetrations, while most high renewable energy studies consider a more balanced mix of renewable energy technologies, with PV typically providing a much lower fraction of annual generation (Cochran et al., 2014).

## Multi-Model Linkage

The general modeling flow follows a sequence common in utility planning practices (such as integrated resource planning). A diagram of this process is provided in Figure S1. This process starts with capacity expansion modeling to determine the cost-optimal buildout, followed by production cost modeling to validate system operability. Limitations revealed by production cost modeling are used to modify capacity expansion scenarios, and the process is repeated until a viable solution is found. Each of these modeling tools, as well as the translation process from various model databases, are described below.

### Capacity Expansion Modeling

Capacity expansion models (CEMs) are tools commonly used by power system planners, policymakers, and other stakeholders to inform investment and retirement decisions for electrical grid assets. CEMs range in spatial scope from a single utility or region (Mai et al., 2015; WECC, 2013) to national tools (Electric Power Research Institute (EPRI), 2017; Eurek et al., 2016; U.S. Energy Information Administration (EIA), 2014). These models typically solve for the least-cost portfolio of generators, transmission, storage, and other resources that are needed to reliably serve load while satisfying a set of economic and policy constraints. Because expansion decisions are usually made in interim steps across a multi-decadal horizon, system operations (including dispatch of generators) are typically simplified and represented at a coarser temporal extent and resolution (Cole et al., 2017; G. Blanford et al., 2016; Pfenninger, 2017). These necessary simplifications with CEMs are the primary reason why multiple steps are often used in grid planning studies.

This study relied on previously published expansion scenarios of the contiguous United States from two capacity expansion models: NREL’s Regional Energy Deployment System (ReEDS) model (for the bulk power system) and the Distributed Generation Market Demand (dGen) model (for distributed PV [DPV] adoption) (Cole et al., 2018b).

ReEDS is developed in the General Algebraic Modeling System (GAMS) (Eurek et al., 2016). ReEDS optimizes the regional mix of technologies that meets the physical and policy requirements of the electric sector at least cost. The model is spatially resolved into 134 regions. Load balancing, planning reserve requirements, and most policy and operational constraints are applied at these 134 regions. These regions are also aggregated into 18 regional transmission operators (RTOs) that approximately represent regional cooperation areas. ReEDS is temporally resolved into 17 “time slices” to capture seasonal and diurnal variations in load and resources. ReEDS optimizes investment decisions within two-year solve periods, sequentially solving from the present-day system out to the model horizon of 2050. ReEDS includes a wide range of generator technology types and spatially resolved resource availabilities from which to make investment and retirement decisions. ReEDS has been designed with special emphasis on capturing the unique traits of variable renewable energy, including VRE capacity credit, VRE curtailment, and transmission, and has regularly been used to examine very high penetration scenarios (Cole et al., 2018a; Mai et al., 2014). We note that ReEDS does not include combined heat and power resources, except for those with the primary purpose of electric generation. The model also does include any changes to the load shape over time that might occur due to technology changes such as electric vehicles, demand response, energy efficiency measures, or climate change (Auffhammer et al., 2017; Mai et al., 2018). However, our total annual load growth incorporates these factors to the extent that they are included in the Annual Energy Outlook load growth projections (EIA, 2016).

All cost and system assumptions for establishing the system buildouts for each scenario are detailed by (Cole et al., 2018b) A summary of key PV and storage cost assumptions and ReEDS buildout values is provided in Table S1 (Cole et al., 2018b). In each scenario, new storage was mostly in the form of batteries, given cost and resource assumptions. The 22 GW of existing pumped-hydro storage was included in each scenario. Storage duration was set to 12 hours for pumped-hydro storage and compressed-air energy storage (CAES) and to 8 hours for battery storage (Cole et al., 2018b). This 8 hours duration is longer than most battery storage currently being deployed (Department of Energy Office of Electricity Delivery and Energy Reliability, n.d.) but enables full capacity credit at higher deployment levels (Denholm et al., 2019). Total annual renewable penetration levels were 41% for the Reference scenario, 48% for Low-Cost PV, and 64% for Low Cost PV+Storage.

Like most CEMS, ReEDS does not inherently include customer adoption of distributed resources. Utility-side investment decisions in ReEDS are based on least-cost investment decisions, subject to meeting load, reliability requirements, transmission constraints, and environmental and policy regulations. Rooftop solar investment decisions are based on a very different set of criteria, including individual end-user choice, so a second model is needed to estimate possible DPV adoption. The dGen model produces scenarios of market uptake of DPV considering the electricity consumption projections for various industrial, residential, and commercial customers; resource and generation capacity potentials in their respective building rooftops; regional electricity rate structures; and estimated cost savings (Sigrin et al., 2016). The model uses Bass diffusion theory and DPV market adoption data to generate projections. The dGen model supports ReEDS by providing exogenously determined DPV projections for consideration in the broader system buildout decision process.

dGen and ReEDS are run iteratively to capture the interaction between the bulk power system and DGPV adoption based on rates. An initial dGen run is used to identify possible adoption if the electricity system were run in a “business as usual” scenario in which rate structures do not change over time. This typically results in relatively high PV adoption, as it does not reflect the decline in PV value that will occur upon large-scale adoption. This initial run is then used by ReEDS to identify impacts on wholesale electricity prices. This impact is used to adjust rate structures, followed by a second dGen run, which modifies customer adoption accordingly. This second run is then used by ReEDS to generate a final build scenario.

The output of the combined CEM is a database of power plant locations, sizes, and transmission capacity. While ReEDS estimates system operational performance during 17 time slices and for annual generation, these results are used only as interim estimates, with more detailed analysis of grid operations performed in the next phase of the analysis.

### Production Cost Modeling

Production cost models (PCMs) are used by system planners, operators, and other decision makers to evaluate the operation of power systems. PCMs study networks ranging in size from small test systems (“RTS-GMLC: Reliability Test System - Grid Modernization Lab Consortium,” 2018) to national- or continental-scale systems (Martinek et al., 2018). These tools economically and chronologically commit and dispatch a fixed fleet of generators with a fixed network topology, often at hourly or subhourly resolution, typically for a full year. Operational decisions minimize the system-wide operating costs, which include variable operations and maintenance, fuel, and startup/shutdown costs. A variety of commercially available PCMs are used by various utility planners. The basic formulation of a PCM is well documented, with the overall objective function of minimizing the system’s operating cost (mainly costs associated with the generator fleet). These costs are shown in a simplified form in Equation 1, where *e_g,t_* is electricity produced by generator *g* at time point *t*; *C_g,t_* is the incremental generation cost for *g* including fuel, variable operations and maintenance, and any emissions costs; *C_g_^su^* is the cost of starting generator *g*; *y_g,t_^su^* is a binary variable denoting startup of generator *g* at time *t;* and *τ* is the set of all time points within the optimization horizon.

| $\sum_{t\in\tau} \sum_{g} \left( C_{g,t}e_{g,t}+C_{g}^{su}y_{g,t}^{su} \right)$ |  | (1) |
| --- | --- | --- |

This cost minimization objective is subject to many constraints. These include constraints for regulating (frequency regulation) reserves, spinning reserves, flexibility reserves, ramping, minimum up and down time, unit commitment, power flow, minimum generation, state-of-charge (for storage), seasonal availability (for hydropower) and unit capacity (e.g., a unit cannot generator more than its rated capacity).

We used a commercial PCM software (PLEXOS) to model hourly system operation (Energy Exemplar, n.d.). The capacity buildout decisions from the ReEDS 2050 solve year were translated into a PLEXOS database to then allow for modeling of unit commitment and economic dispatch in PLEXOS. The model uses a zonal resolution (134 zones, with DC power flow and transmission flow limits between regions, but no intra-regional transmission representation). A map defining the ReEDS 134 load balancing areas (BAs) and 18 regional transmission organization (RTO) regions is shown in Figure S2. Mixed integer programming (MIP) simulations were performed hourly for a single year. To improve run time, each month of the year was run in parallel, and the 12 monthly runs were then combined to provide annual results, where an additional 24-hour look-ahead informs but does not extend the solution beyond the last time step in each month. For discussion of temporal decomposition of the unit commitment problem, see (Barrows et al., 2014).

### ReEDS-to-PLEXOS Data

ReEDS/PLEXOS share many input data sets. The values used in ReEDS are documented in detail by (Cole et al., 2018b) and in the ReEDS documentation (Eurek et al., 2016). This includes hourly load from 2006 (Eurek et al., 2016), hourly PV generation profiles from the National Solar Resource Database using 2006 meteorological conditions (Habte et al., 2017), and hourly wind profiles from AWS Truepower for a typical meteorological year (DOE, 2015); 2006 was identified as a typical year based on previous analysis (Lew et al., 2013).

Each of the 134 regional fuel prices for natural gas, coal, and uranium from ReEDS are passed directly to PLEXOS, and generator emission rates are consistent between both models. Natural gas prices varied between each of the scenarios modeled here because of the elasticity of natural gas prices assumed in the development of these scenarios. Table S2 summarizes the natural gas fuel prices across the 134 PLEXOS regions that were used for each scenario. Fuel prices are divided into winter (January, February, November, and December) and non-winter (March through October) seasons.

Because ReEDS is a linear program, generators may be constructed in continuous sizes. As a result, some generator properties were adjusted when converted into the PLEXOS database. For example, ReEDS might build 1,000 MW of new natural-gas CC (NG-CC) capacity in a region and treat that capacity as a single model plant. However, if a 1,000 MW plant were represented in a unit commitment and dispatch model, that plant’s outage and unit commitment would have outsized impacts on the system operation. Therefore, in any region where such a model plant exceeds the size of the largest plant for a given generator type from NREL’s Low Carbon Grid Study (LCGS) database (Brinkman et al., 2016), the model plant is disaggregated into multiple plants, with the new plants assuming the average plant size for that generator type in the LCGS database. Throughout this subdividing process, the total generation capacity is maintained between ReEDS and PLEXOS. Similar aggregation was performed for smaller plants.

Generator heat rates were also modified to provide a range of values across units in PLEXOS. Many of the new conventional technologies in ReEDS have the same heat rate (e.g., all NG-CC units built after 2030 have identical heat rates in ReEDS). These repeated heat rates can cause issues with computational degeneracy in PLEXOS. To avoid these issues, we randomly adjusted the heat rates of new conventional generators by ±5%. Other generator parameters were derived from the LCGS database and include minimum up time, minimum down time, forced outage rate, maintenance rate, mean time to repair, startup cost, minimum generation level, and maximum ramp rate. The specific operational parameters used, which again were unique to PLEXOS and not specified in ReEDS, are shown in Table S3 for each technology type. These parameters were not specified for the VRE technologies. In addition, the following parameter values were assumed for storage: battery maintenance rate of 0.55%, battery forced-outage rate of 2%, pumped-hydro storage maintenance rate of 3%, pumped-hydro storage forced-outage rate of 3.8%, CAES maintenance rate of 4%, CAES forced-outage rate of 3%, and CAES minimum stable level of 0.25. Lastly, all hydropower generators were modified in PLEXOS to be must run. We note that only a limited set of hydropower usage constraints were represented, as reflected by the historic capacity factor and minimum operating level values from ReEDS; for example, some environmental constraints that could impact the dispatch level of hydro were not fully represented.

During its creation, the LCGS data set was thoroughly vetted with input from a technical advisory committee, which included utility, regulatory, and other stakeholder entities. Particular attention was given to ramp rates and start costs, which are important factors in determining unit commitment and dispatch, especially in high VRE systems with greater requirements for thermal cycling and ramping. Because if their importance, the resulting industry-vetted ramp rates and start costs were intentionally conservative (i.e., more restrictive).

The other key modification in the ReEDS-to-PLEXOS conversion process is the representation of transmission losses. PLEXOS does not represent transmission losses in the formulation used here, so transmission losses from ReEDS were included in PLEXOS as additional load in the regions sending the power in order to maintain consistent generation requirements between ReEDS and PLEXOS.

Operating reserves were enforced at the RTO level, similar to current utility practices. We enforce three reserve products: spinning contingency reserves, regulating reserves (frequency regulation), and a flexible ramping reserve. Spinning reserves are used to address large generator or transmission line failures, while regulation and flexibility reserves are used to address both normal and unscheduled variability in net demand over sub-hourly timescales. The reserve requirements enforced by PLEXOS in each time step are assumed to be 3% of load for spinning, 1% of load for regulation, and 2% of VRE generation for flexibility. Each of these types of reserves can be held by partially loaded generators with sufficient ramping capability to respond in a given timeframe, including energy storage. All reserve requirements are enforced through a proxy for the scheduling—but not releasing—of that capacity. For regulating reserves, these requirements approximate the amount of capacity to hold back at an hourly resolution that would be needed, on average, at the 4-second frequency regulation timescale. However, curtailed VRE energy is not assumed to be available to provide any reserves in these scenarios. This is a conservative assumption. Although some locations in the U.S. do not currently allow VRE to provide reserves, others locations do, and tests have demonstrated that pre-curtailed PV can follow a regulation signal with greater speed and accuracy than conventional thermal power plants (Loutan et al., 2017).

## Validation and Iteration

As discussed, PCM simulations act to validate CEM buildouts as well as provide additional results. The initial CEM buildout in the Low Cost PV+Storage scenario resulted in an unreliable power system, particularly in the EI and ERCOT. Specifically, load was dropped during 168 hours per year, with about 1.2 terawatt-hours (TWh)—0.02% of total load—unserved. The PLEXOS modeling revealed that ReEDS overestimated the ability of storage to meet demand. The original assumption was that 8-hour storage can provide nearly full capacity credit (97%) (Sioshansi et al., 2014). More recent work has found that at increasing penetration, the capacity credit of storage drops, and this was identified as an issue in the PLEXOS modeling (Denholm and Margolis, 2018; Frew et al., 2018). To correct this, the storage capacity credit was derated in the EI and ERCOT, and the ReEDS scenario was rerun. This resulted in a new scenario with additional thermal capacity (and storage), as reported in the Results section. This new scenario was then run in PLEXOS, which found no instances of dropped load (or significant reserve violations).

The final scenario results in this work used the capacity mix from ReEDS and dGen, but all operational results presented are from the PLEXOS production cost model. In this way we were able to leverage a capacity expansion model for investment and capacity adequacy decisions and an hourly, chronological production cost model for operation decisions, and then validate the results.

# Additional Modeling Details

## ReEDS-to-PLEXOS Conversion Process

The full modeling workflow used for this analysis (refer back to Figure S1) included exogenous distributed PV adoption from dGen, capacity expansion by ReEDS, and operational modeling by PLEXOS, with iterations between each. This section provides more details on the ReEDS-to-PLEXOS conversion process within that workflow. This step takes a ReEDS model solution and creates an input database for the PLEXOS production cost model. Where reasonable, the values or settings from the ReEDS model are maintained in PLEXOS. These settings include:

- Regions: The 134 ReEDS regions are converted to 134 PLEXOS nodes (see “Model Regions” section below).
- Transmission flow limits: transmission flow limits between regions (nodes) are the same between models, and both models use a DC power flow.
- Fuel prices: Each of the 134 regions has the same fuel price in PLEXOS and ReEDS. Natural gas prices are different at each node, but the same between the models.
- Generator emission rates
- Load and renewable energy generation profiles: ReEDS uses the hourly load and renewable energy profiles for estimating renewable energy curtailment and capacity value. These hourly profiles are passed to PLEXOS. Each of the 134 regions has a unique load profile. For renewable energy, there is a unique generation profile for each technology and resource class in each region.

Other values were slightly adjusted as they were converted to PLEXOS. These values include:

- Generator capacities: ReEDS combines similar plants with the same cooling technology in a region into a single model plant.^^[[1]](#footnote-2)^^ For example, a 300-MW NG-CC unit with recirculated cooling and a 200-MW NG-CC unit with recirculated cooling are seen as a single 500-MW plant in ReEDS. In any region where such a model plant exceeds the size of the largest plant for a given generator type in the Western Electricity Coordinating Council (WECC) Transmission Expansion Planning Policy Committee (TEPPC) database, the model plant is subdivided into multiple plants, with the new plants assuming the average plant size for that generator type in the WECC TEPPC database. Throughout this subdividing process, the total generation capacity is maintained between ReEDS and PLEXOS.
- Small generators: Any generators less than 10 MW in size are aggregated with any other small generators of the same technology type in the state. If, after this aggregation, any units with less than 5 MW of capacity remain, they are removed. These small units dramatically increase the runtime of the production cost model, which is why they are removed via aggregation or deleted.
- Heat rates: Many of the new conventional technologies in ReEDS have the same heat rate (e.g., all NG-CC units built after 2030 have identical heat rates in ReEDS). These repeated heat rates can cause issues with degeneracy in PLEXOS. To avoid these issues, we randomly adjusted the heat rates of new conventional generators by ±5%.
- Transmission losses: PLEXOS does not represent transmission losses, so the transmission losses in ReEDS were included as additional load in the regions sending the power.

Finally, other values are required for PLEXOS, but these values do not exist in ReEDS or were superseded by values typically used in PLEXOS. These values were taken from the WECC TEPPC database and include:

- Minimum up time
- Minimum down time
- Forced outage rate
- Maintenance rate
- Mean time to repair
- Startup cost
- Minimum generation level
- Maximum ramp rate.

The values adopted for the PLEXOS modeling are described below in the section “PLEXOS Input Parameters.” A comparison of the resulting generating capacity (all in AC) in PLEXOS versus the original ReEDS fleet is shown in Figure S3 for each of the three scenarios.

## Modifications to Low-Cost PV+Storage Scenario

Our Low-Cost PV+Storage scenario buildout was modified slightly from the native scenario in (Cole et al., 2018b) to ensure sufficient capacity was deployed to meet load at all hours during system operations. See the Methods section for details on these changes, which reflect the importance of accurate capacity valuation for storage during the system planning process.

In order to remedy the dropped load observed in PLEXOS for the Low-Cost PV+Storage scenario (see Table S4), we modified the battery storage capacity value. Our adjusted battery storage capacity value in ReEDS derated the capacity contribution of storage by the maximum hourly quantity of dropped load (Equation 1), effectively assuming that all previously dropped load was due to a storage capacity shortage. The Low-Cost PV+Storage ReEDS 2050 buildout year was rerun with this storage capacity value adjustment, assuming the same PV buildout as the initial run. The assumed storage capacity value and resulting amount of installed storage capacity are summarized in Table S4.

One additional dimension of this storage capacity value is the underlying data used to inform the storage capacity value modification. The initial storage capacity was assumed to be a static 0.97. As shown in Table S4, load was not dropped in the Western Interconnection, and thus the larger storage capacity value was an appropriate assumption. These differences in relationship of storage capacity value to system capacity adequacy are impacted, at least in part, by regional differences in load, VRE profiles, and the underlying thermal fleet. These results support the need to develop a more robust method to calculate storage capacity value in ReEDS that accounts for regional specificity, as well as the declining value of capacity.

*Equation 2: Calculating storage capacity value for scenarios with dropped load by interconnection*

$${StorCV}_{new}= \frac{StorCap*{StorCV}_{old}-{max(ShedLoad}_{hour})}{StorCap}$$

Where $StorCap$ is the installed storage capacity, ${ShedLoad}_{hour}$ is the amount of load dropped in each hour, ${StorCV}_{old}$ is the storage capacity value used in ReEDS (in this case with 8-hour storage, this is 0.97), and ${StorCV}_{new}$ is the new calculated storage capacity value.

# Additional Results

## Peak Load and Net Load Hours

The total contribution of PV and storage during nationwide peak load and net load hours is summarized in Table S5 for each scenario. Peak load occurs on August 4 at 5 p.m. for all scenarios. Peak net load occurs on August 10 at 8 p.m. for the Reference and Low Cost PV scenarios and December 14 at midnight for the Low Cost PV+Storage scenario. The peak net load values correspond to the nationwide dispatch plots shown in Figure 2 of the main text.

Table S5 also reflects the important role that storage plays in the utilization of PV. As shown in Table 1 of the main text, the Low Cost PV+Storage scenario has the highest instantaneous curtailment in GW. However, as shown in Table S5 here, this scenario also sees the greatest overall utilization of PV due to the availability of storage. Note that not all curtailed energy recovered by storage is used because of the losses associated with charging and discharging.

Another way of looking at this important role of storage is the dramatic increase in peak storage usage, both for charging and discharging, as we move from the Reference to the Low-Cost PV and Low-Cost PV+Storage scenarios: roughly 2x from the Reference to Low-Cost PV for both charging and discharging penetrations, 11x from the Reference to Low-Cost PV+Storage for discharging, and 8x from the Reference to Low-Cost PV+Storage for charging. This reveals the significant value of storage in enabling a high PV penetration future, particularly in meeting load during periods with little to no solar via storage discharging.

## Thermal Generator Cycling

As PV is added (moving from the Reference to Low-Cost PV+Storage scenario) the average ramping of the nationwide CC fleet increases from 13%/hour to 37%/hour in the upward direction and 11%/hour to 37%/hour in the downward direction across these cases. In all scenarios, 100%/hr up and down ramp rates (i.e., ramping at the full installed capacity across a single hour) were observed, and the frequency of such full ramp rates increased by 4x-5x between the Reference and Low Cost PV+Storage scenarios.

## Curtailment

Total annual curtailment by ReEDS BA region is shown in Figure S4. Spatially, curtailment is roughly correlated to the areas with greatest PV deployment. Total curtailment by hour of day and month for each interconnection as a percentage of total wind and solar generation during each hour-month time block, including curtailed energy, are shown in the heat map in Figure S5. Temporally, curtailment is also roughly correlated to the times of solar generation and increases in intensity as more PV is deployed from the Reference to Low-Cost PV to Low-Cost PV+Storage scenarios.

## RTO-Level Dispatch for Day with Peak Load and Peak Net Load Hour

All 18 RTO daily dispatch plots for each scenario for the day with the peak load hour and they day with the peak net load hour (no storage) are shown in Figure S6 through Figure S23. These RTOs are defined in Figure S2. Nationwide results are shown in the main text Figure 2.

## RTO-Level Daily Dispatch of Maximum 3-Hour Up/Down System Ramps

All RTO daily dispatch plots for maximum upward/downward 3-hour ramp events are shown in Figure S24 through Figure S41; maximum upward 3-hour ramp events are shown for CAISO and ERCOT in the main text Figure 3. These RTOs are defined in Figure S2. We note that curtailments below the “load with storage” line indicate times when a region is curtailing zero-cost generation and also importing zero-cost generation. This reflects degeneracy in the model, where curtailment is occurring in multiple adjacent regions. We do not model “hurdle rates” or other sources of friction between regions to avoid these sorts of outcomes.

# Figures and Tables


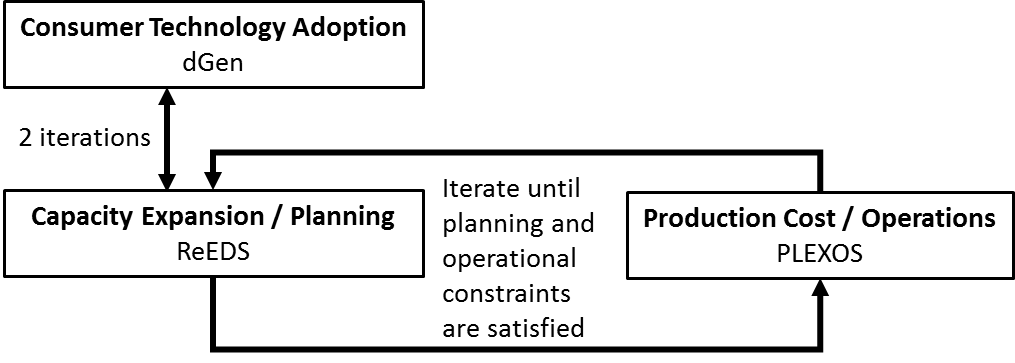


Figure S1. Iterative modeling workflow used for the analysis, related to all results (main text Figures 1-6).

Figure S2. ReEDS 134 BA regions and 18 RTOs, related to regional results in Figures 3-5 and Figures S4-S41.


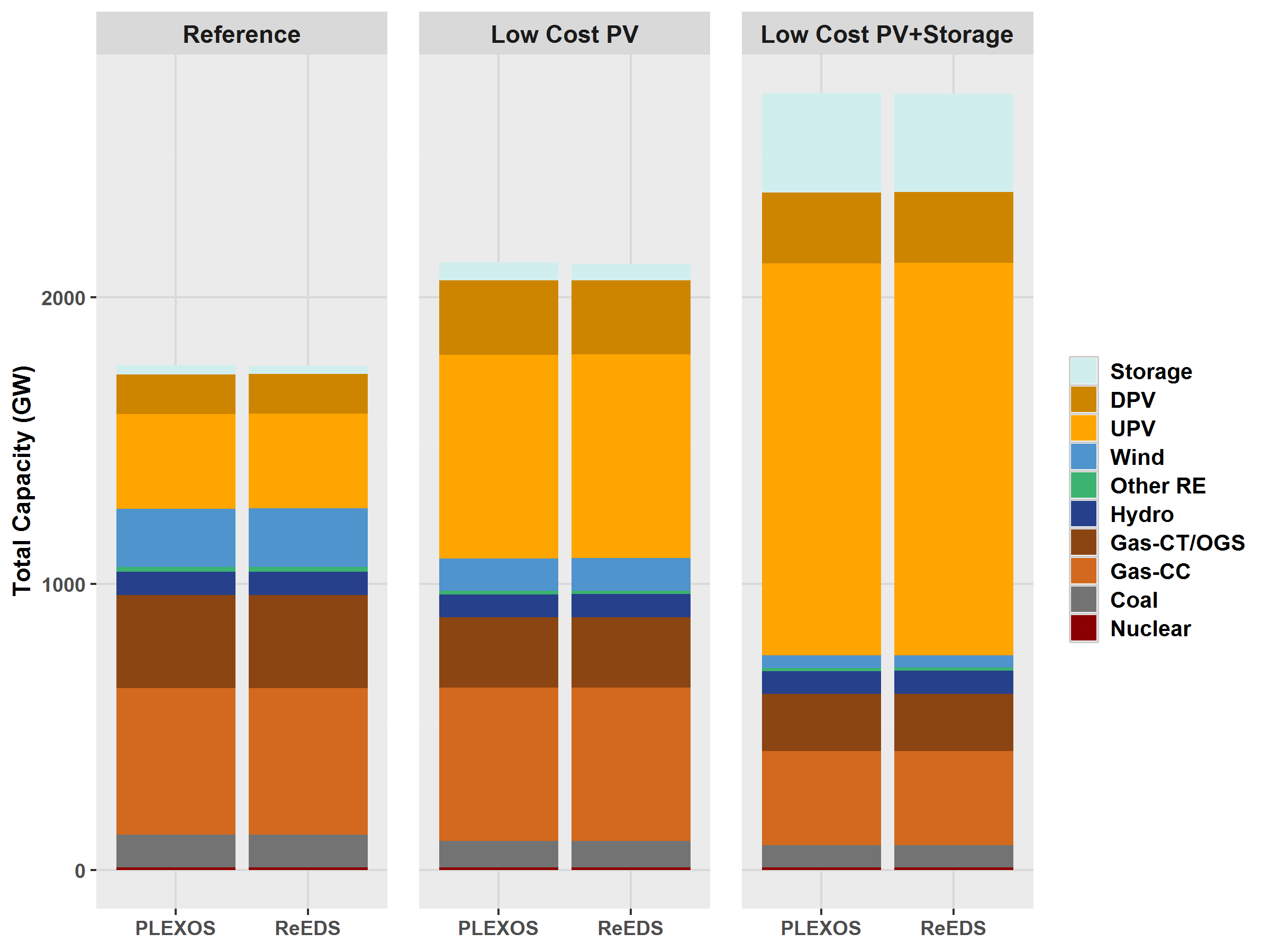


Figure S3. Installed capacity comparison between PLEXOS and ReEDS runs, related to all results (main text Figures 1-6).


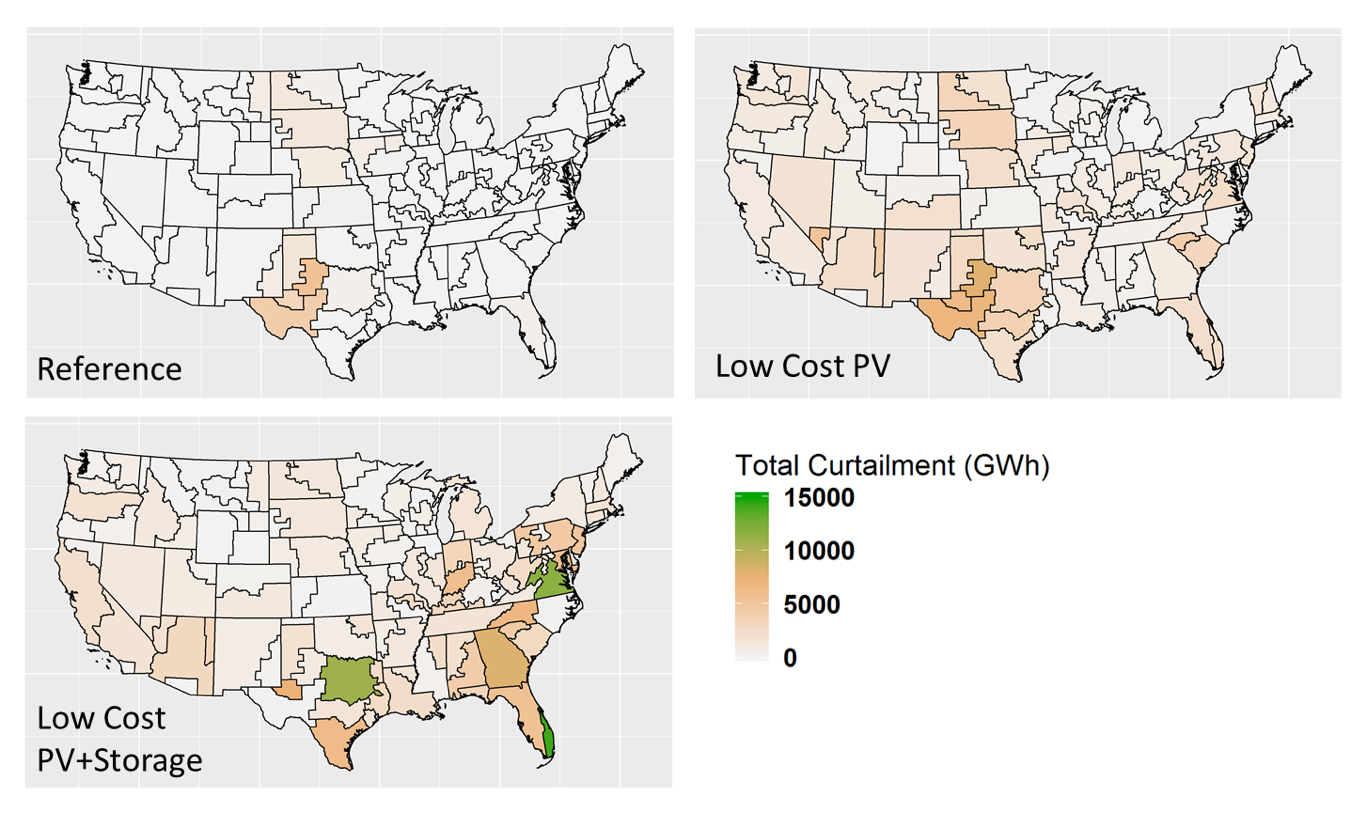


Figure S4. Total annual curtailment by ReEDS BA region, related to Figure S2 and Table 1.


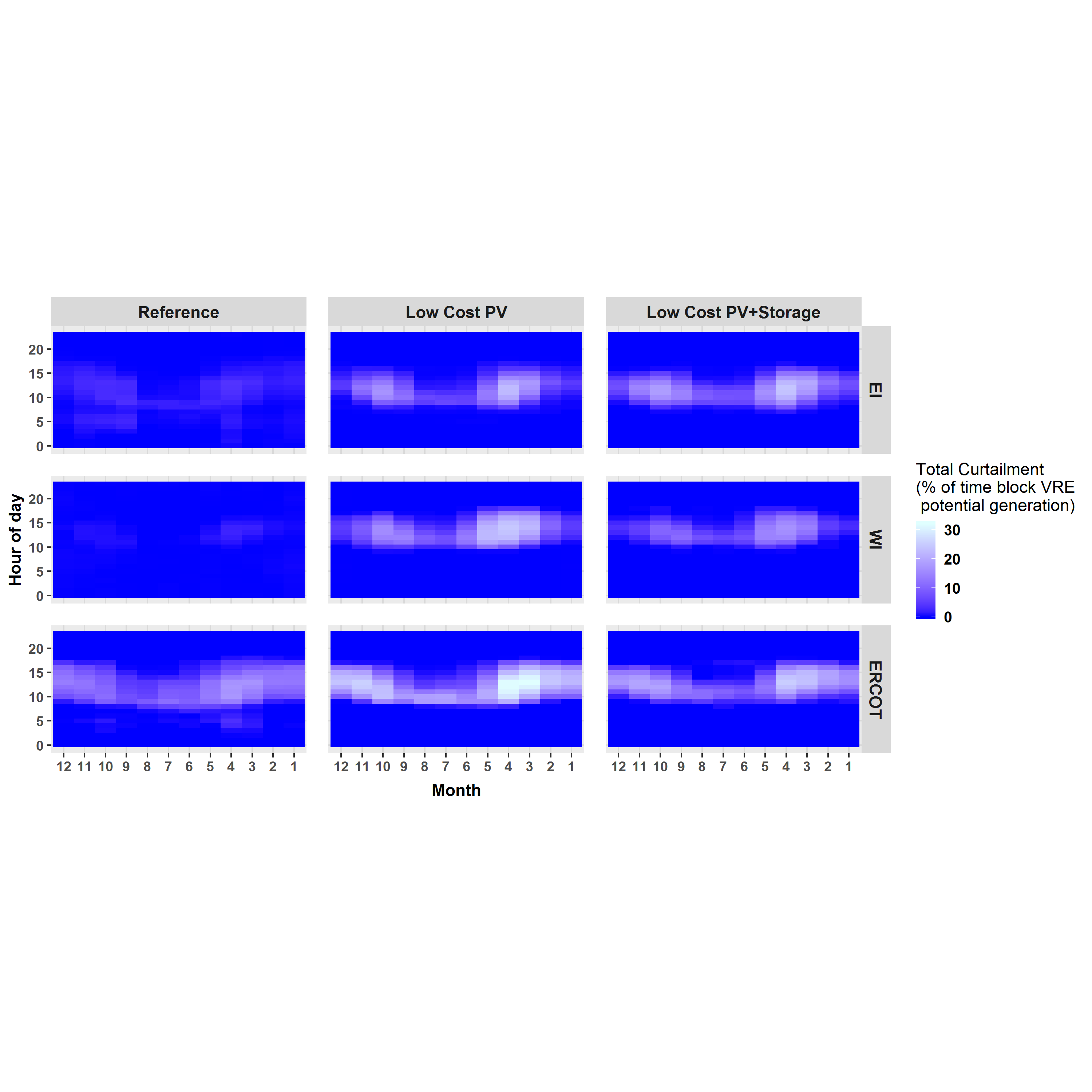


Figure S5. Total curtailment by hour and month as a percentage of total potential VRE during each hour-month time block by interconnection. Notes: EI = Eastern Interconnection, WI = Western Interconnection, ERCOT = Electric Reliability Council of Texas (ERCOT) Interconnection. See also Figure S2 and Table 1.


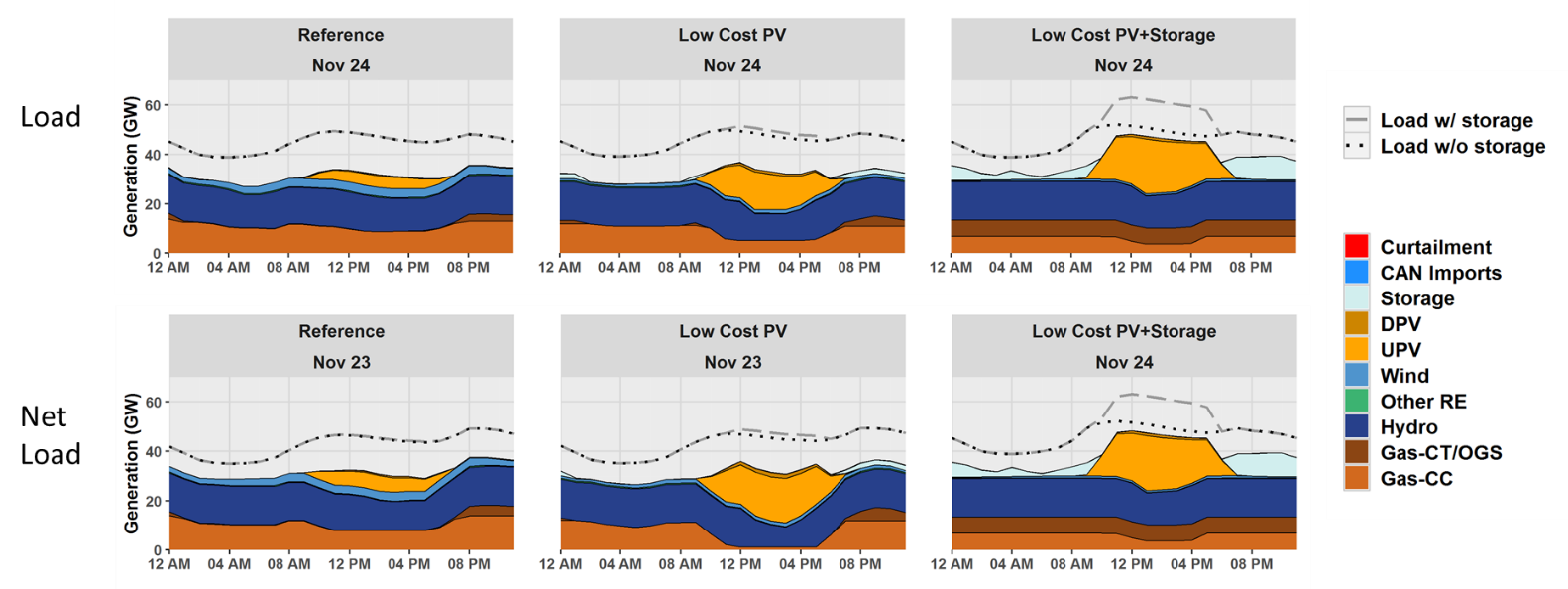


Figure S6. BPA hourly dispatch for each scenario's day with peak load and net load hour, related to Figure 2 and Figure S2.


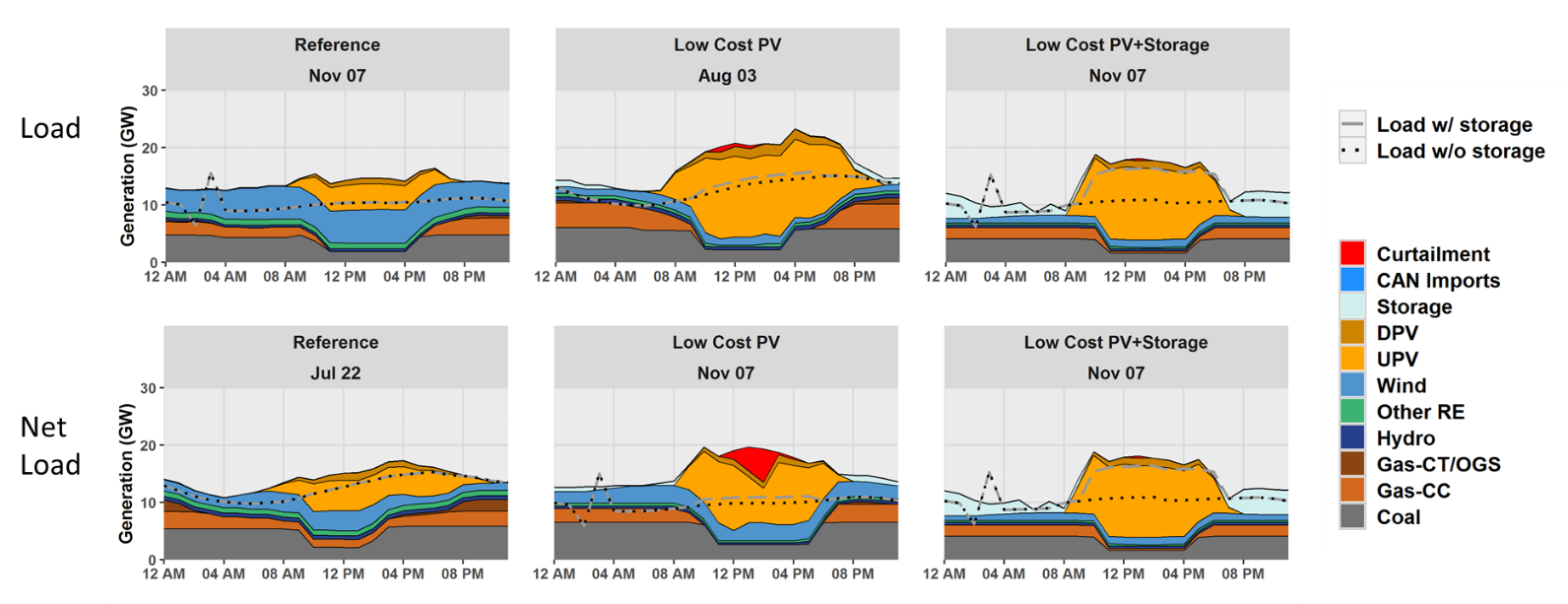


Figure S7. NWPP hourly dispatch for each scenario's day with peak load and net load hour, related to Figure 2 and Figure S2.


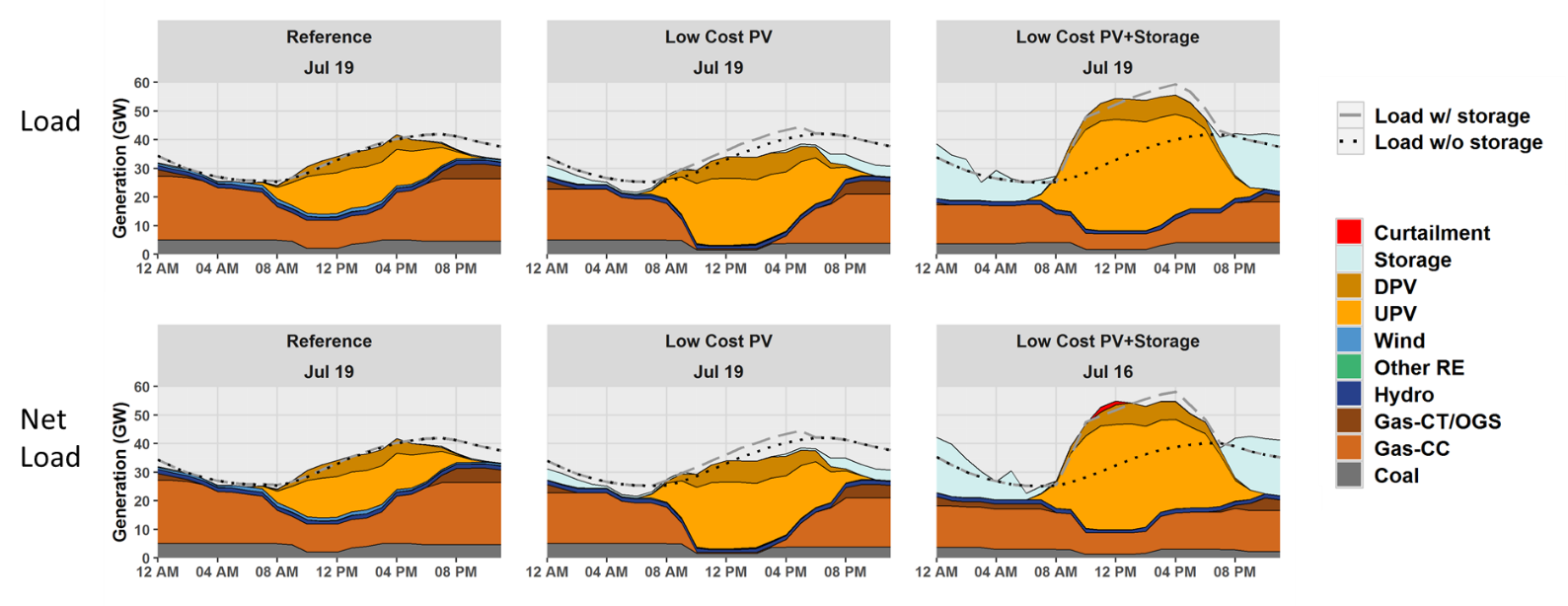


Figure S8. AZNM hourly dispatch for each scenario's day with peak load and net load hour, related to Figure 2 and Figure S2.


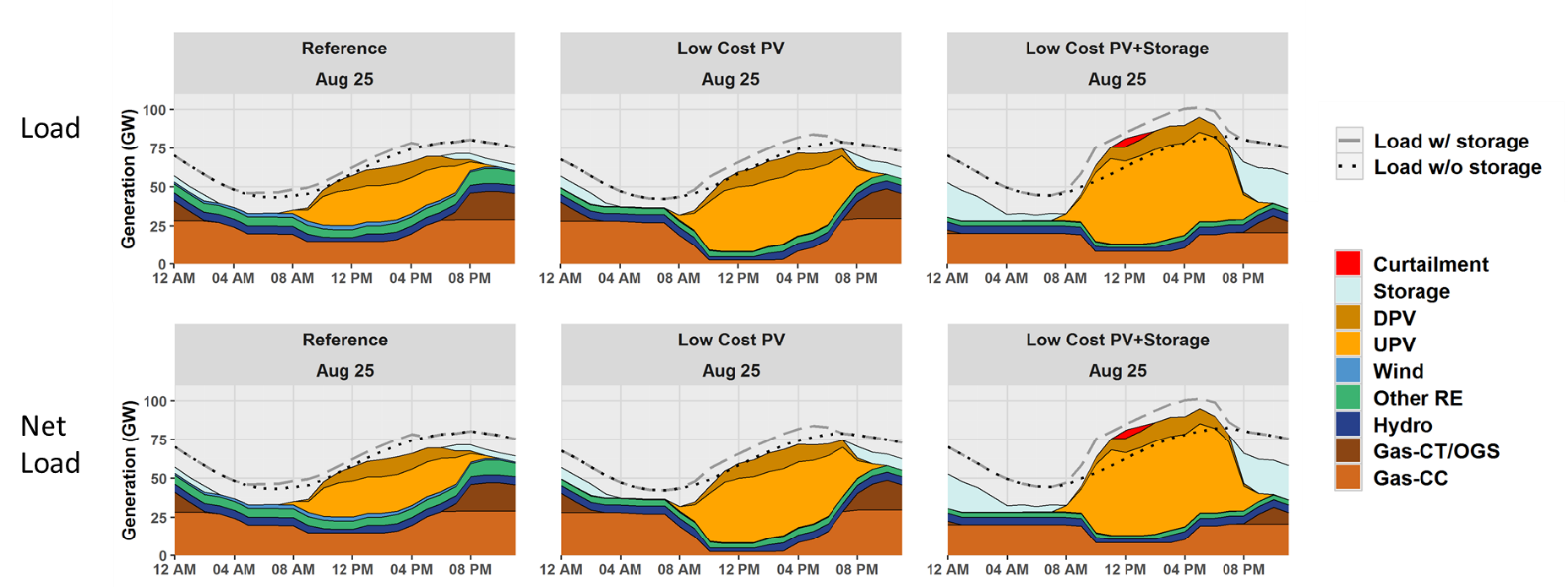


Figure S9. CAISO hourly dispatch for each scenario's day with peak load and net load hour, related to Figure 2 and Figure S2.


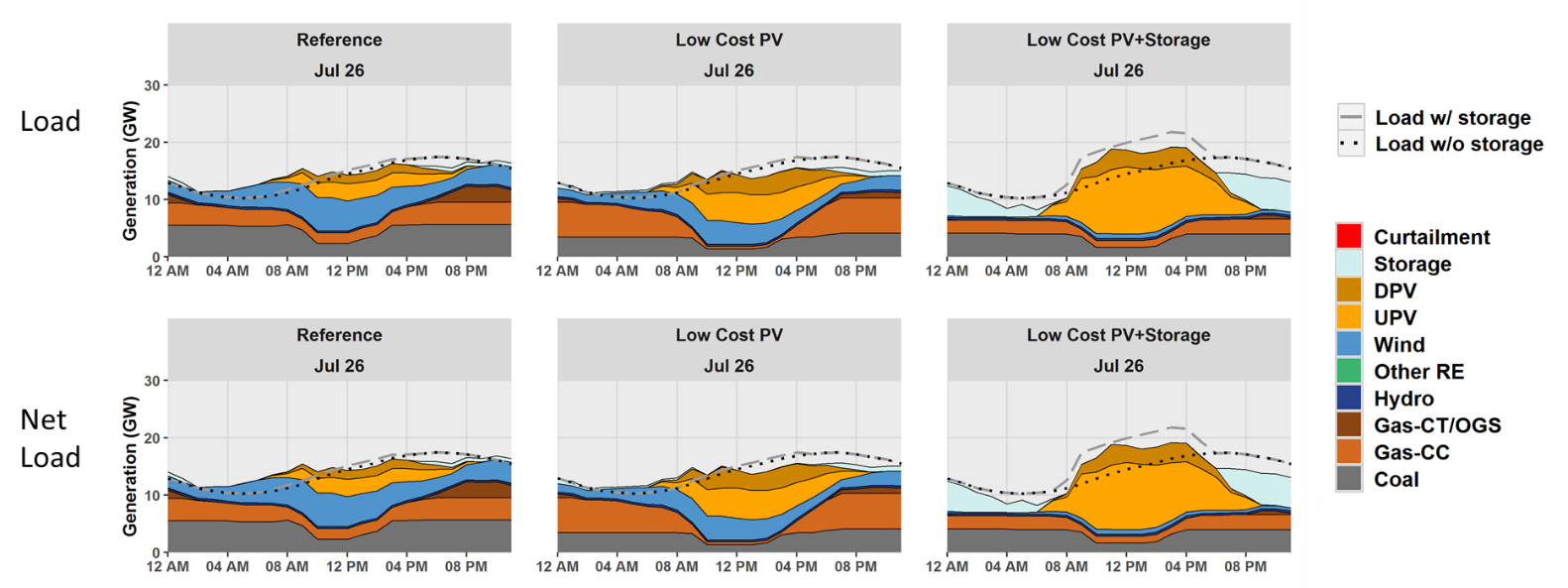


Figure S10. RMPP hourly dispatch for each scenario's day with peak load and net load hour, related to Figure 2 and Figure S2.


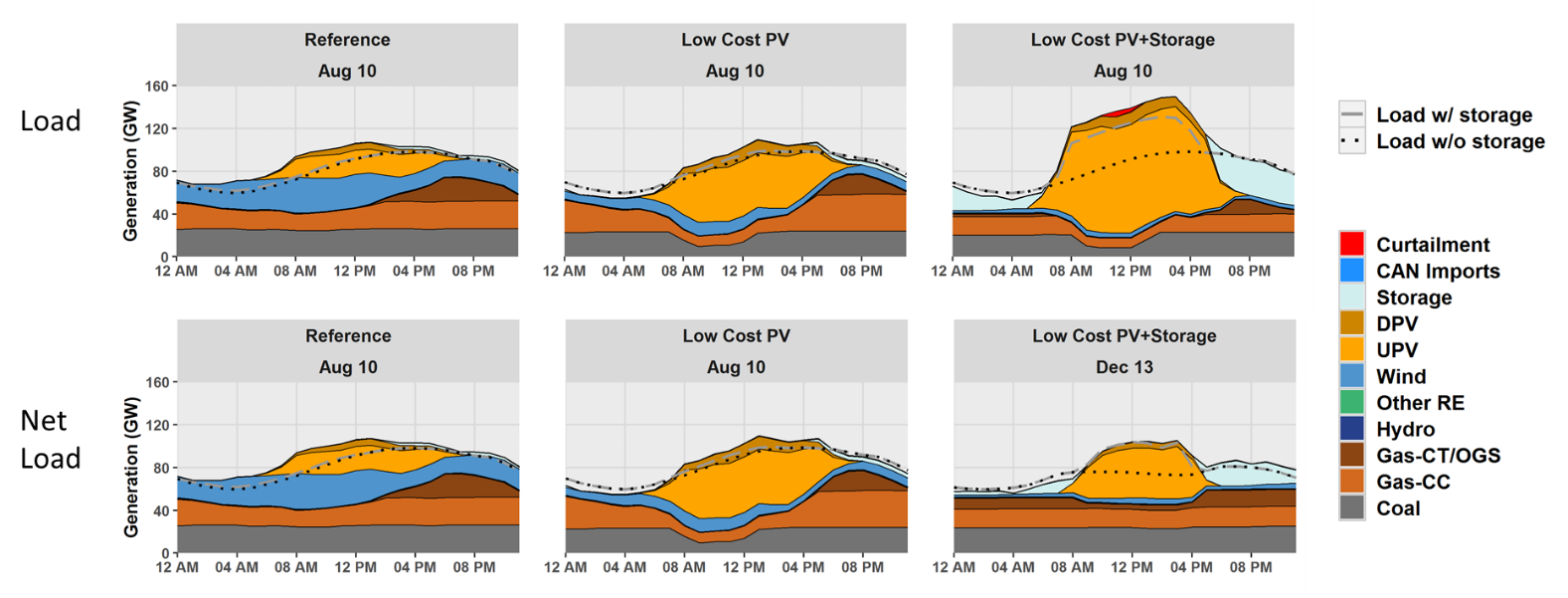


Figure S11. MISO-E hourly dispatch for each scenario's day with peak load and net load hour, related to Figure 2 and Figure S2.


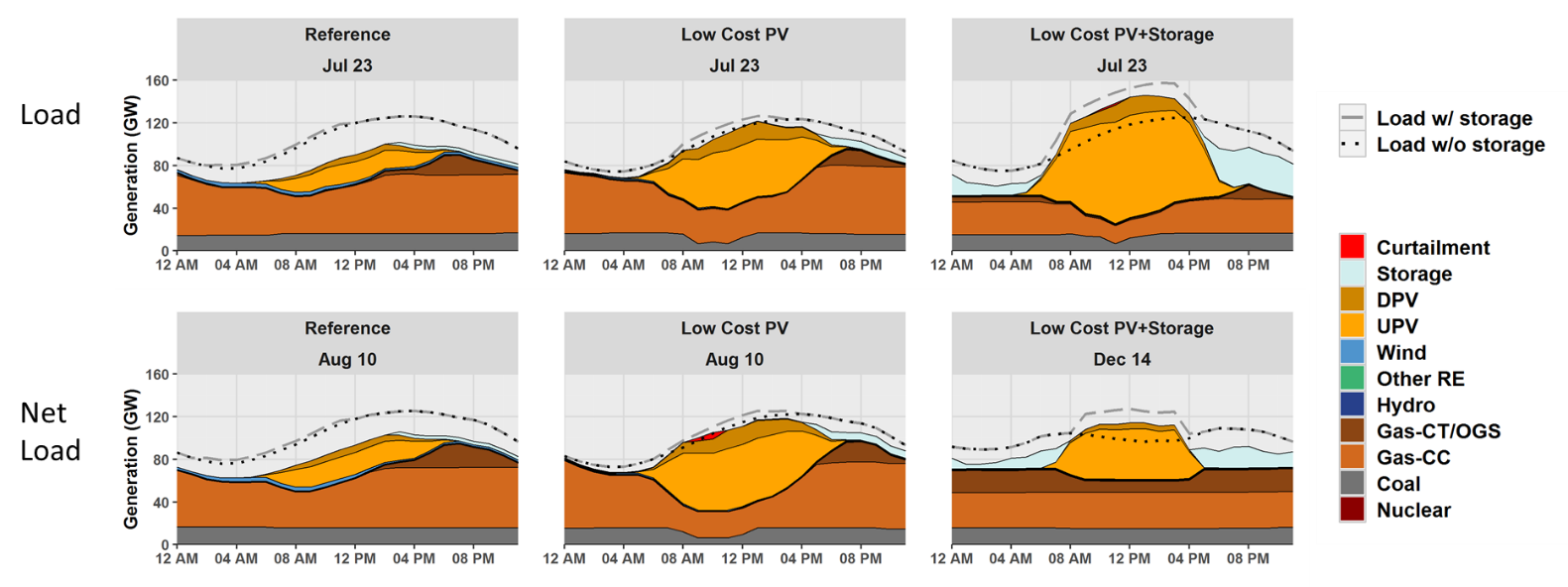


Figure S12. PJM-W hourly dispatch for each scenario's day with peak load and net load hour, related to Figure 2 and Figure S2.


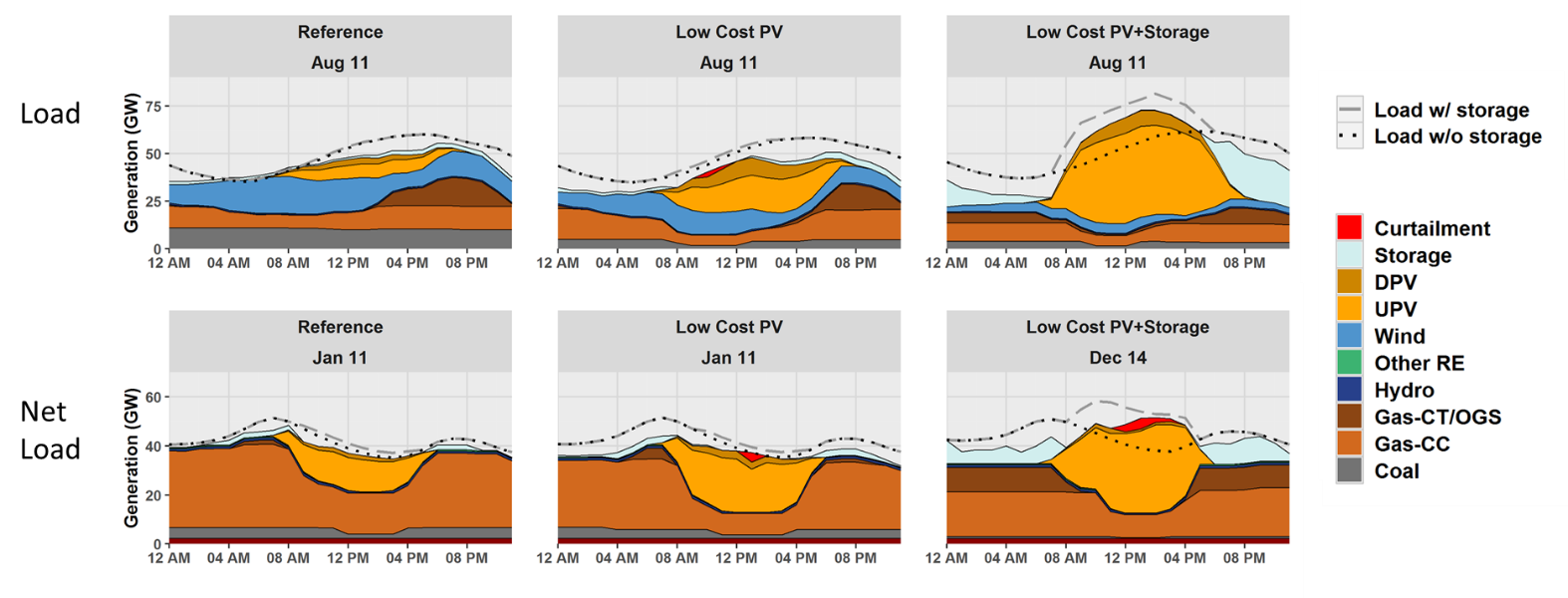


Figure S13. SPP hourly dispatch for each scenario's day with peak load and net load hour, related to Figure 2 and Figure S2.


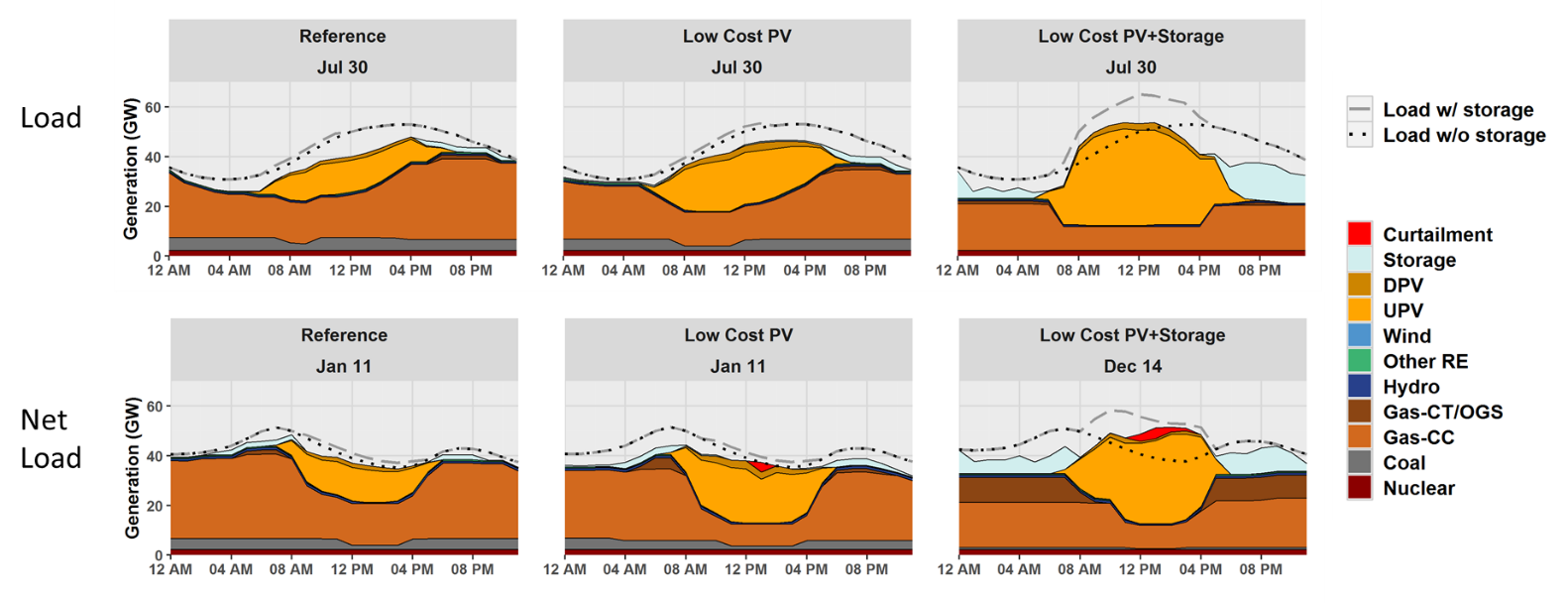


Figure S14. SE hourly dispatch for each scenario's day with peak load and net load hour, related to Figure 2 and Figure S2.


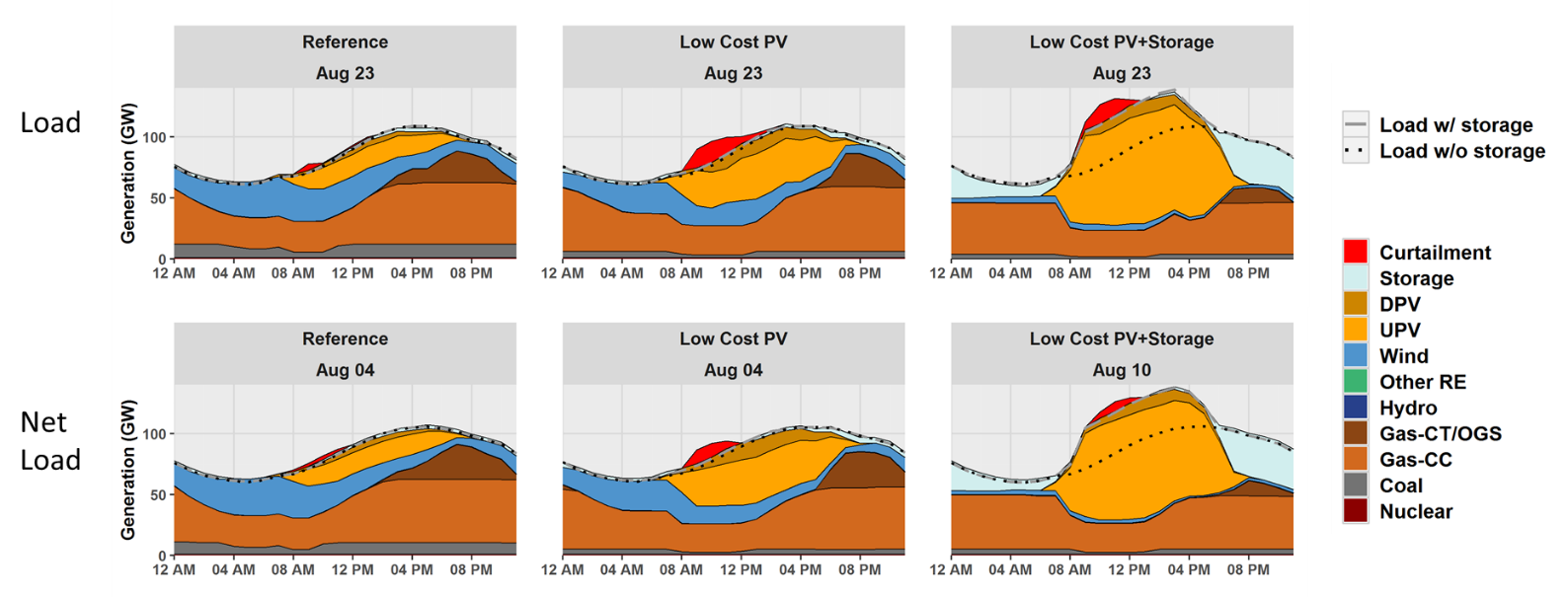


Figure S15. ERCOT hourly dispatch for each scenario's day with peak load and net load hour, related to Figure 2 and Figure S2.


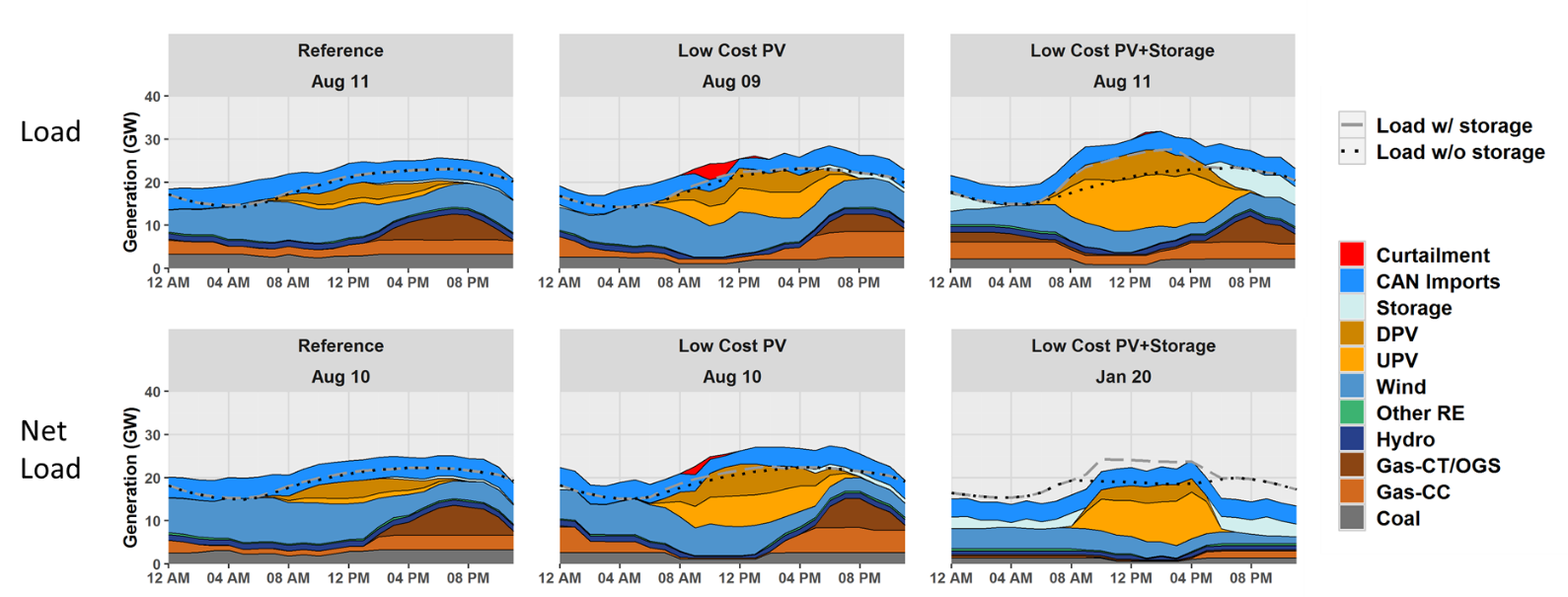


Figure S16. MISO-W hourly dispatch for each scenario's day with peak load and net load hour, related to Figure 2 and Figure S2.


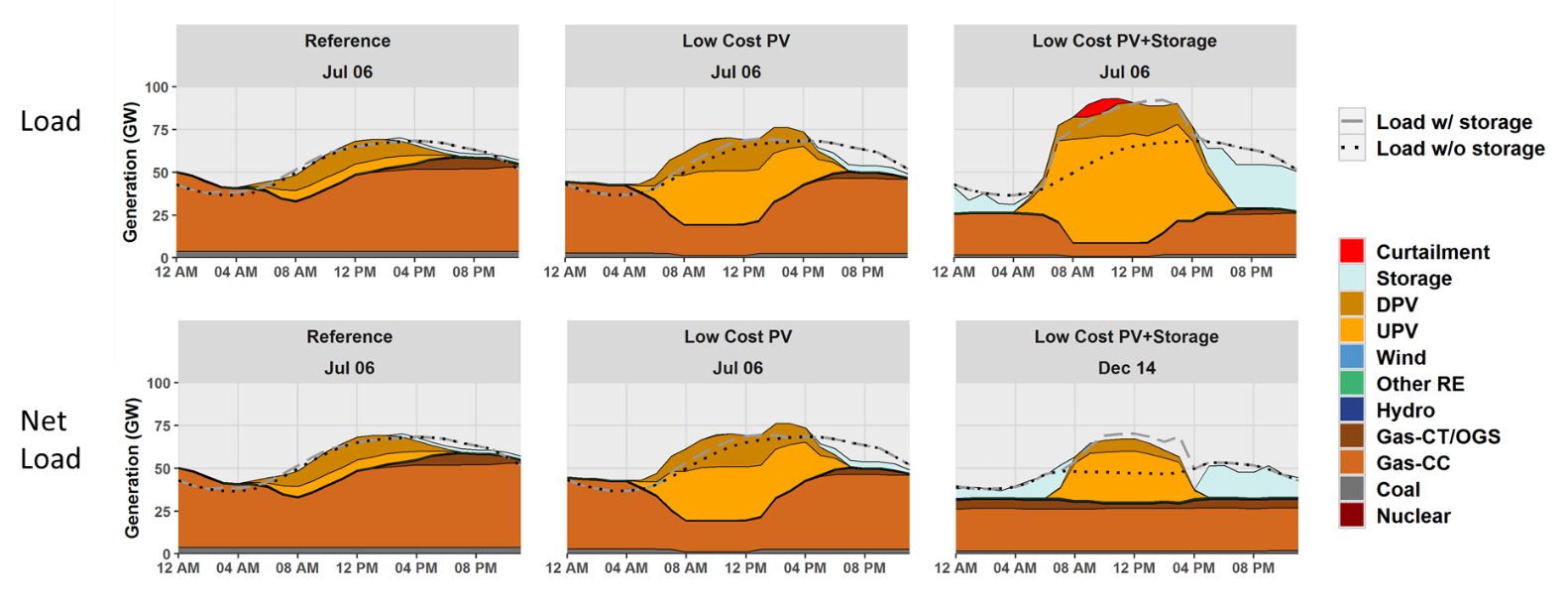


Figure S17. PJM-E hourly dispatch for each scenario's day with peak load and net load hour, related to Figure 2 and Figure S2.


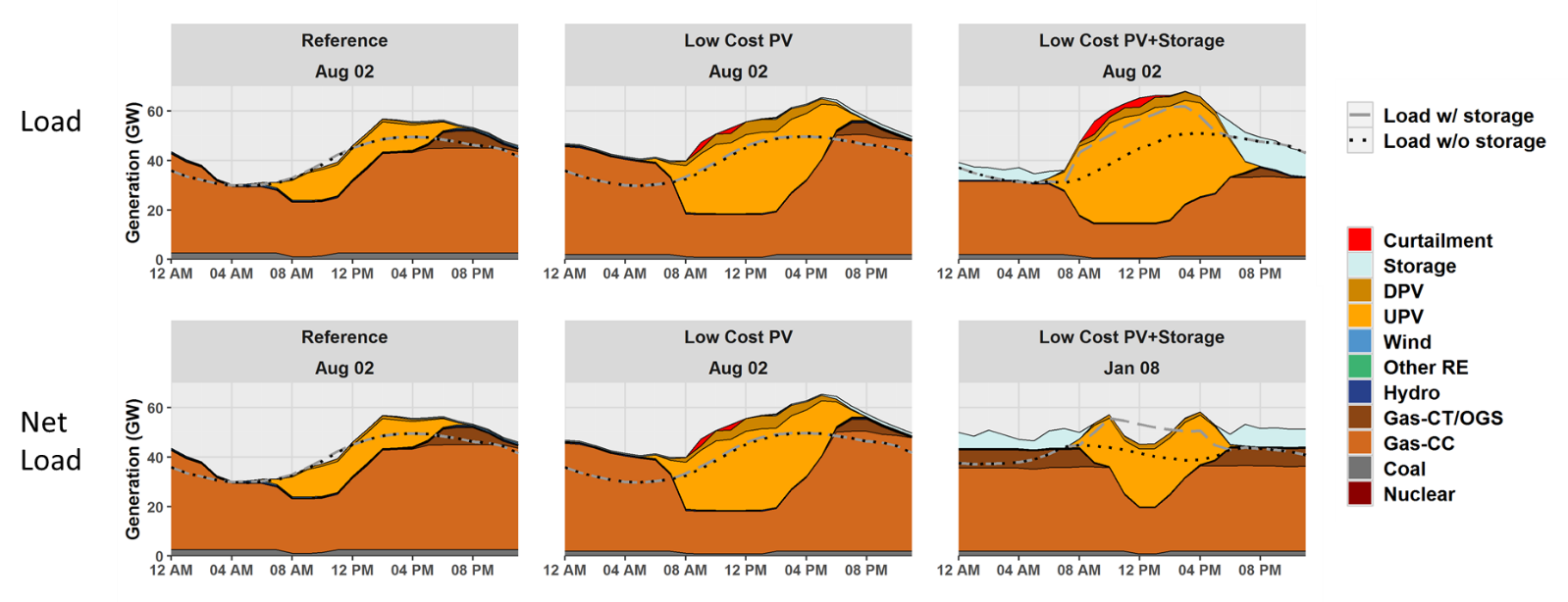


Figure S18. MISO-S hourly dispatch for each scenario's day with peak load and net load hour, related to Figure 2 and Figure S2.


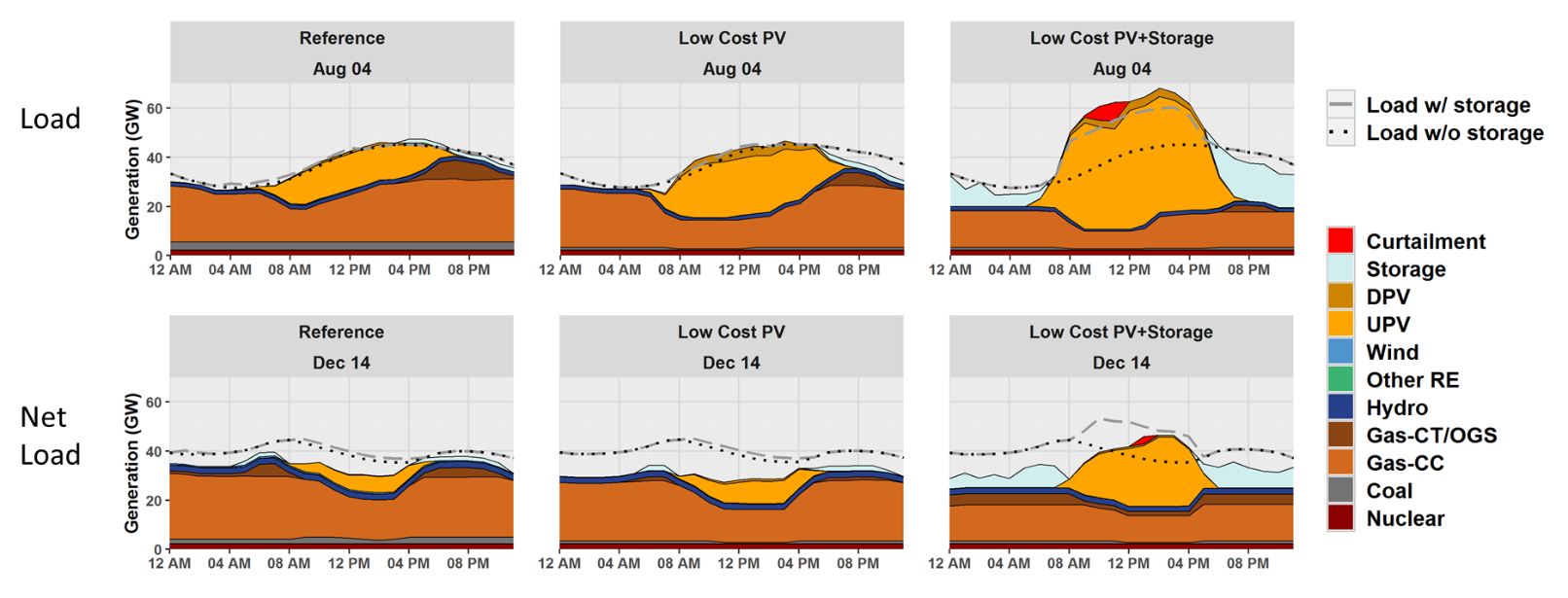


Figure S19. TVA hourly dispatch for each scenario's day with peak load and net load hour, related to Figure 2 and Figure S2.


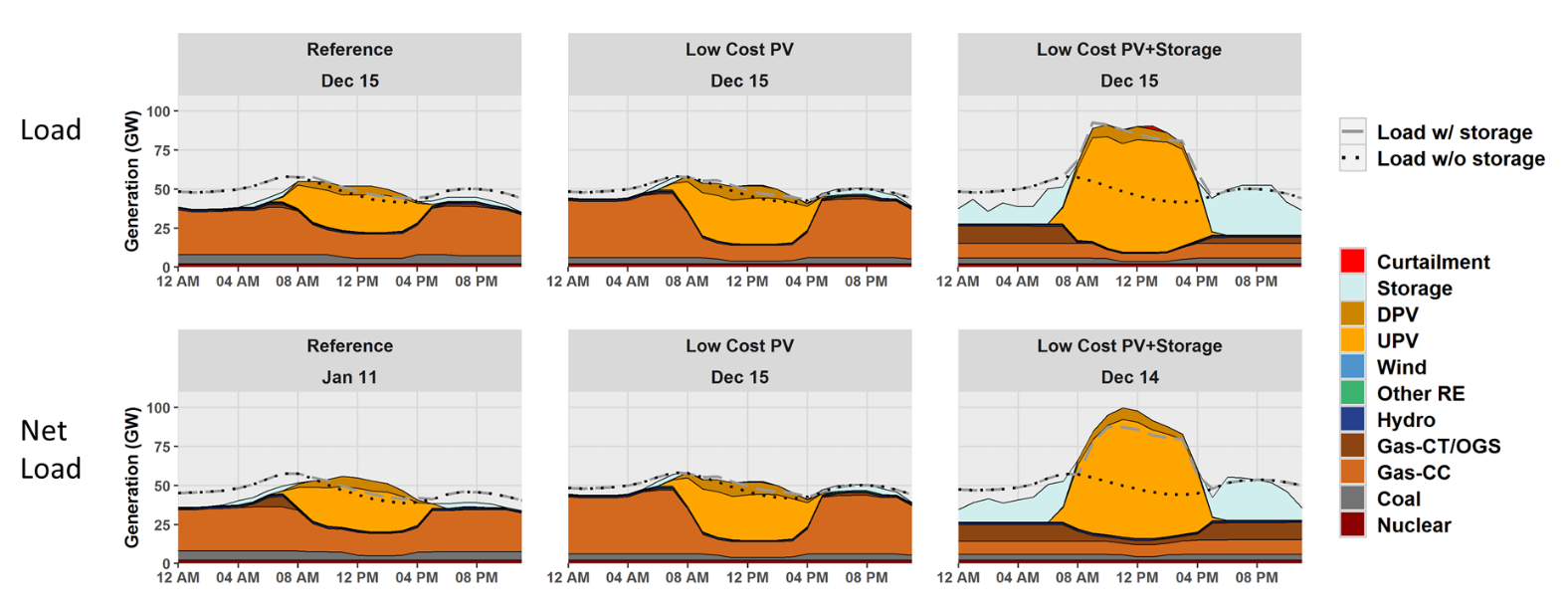


Figure S20. VACAR hourly dispatch for each scenario's day with peak load and net load hour, related to Figure 2 and Figure S2.


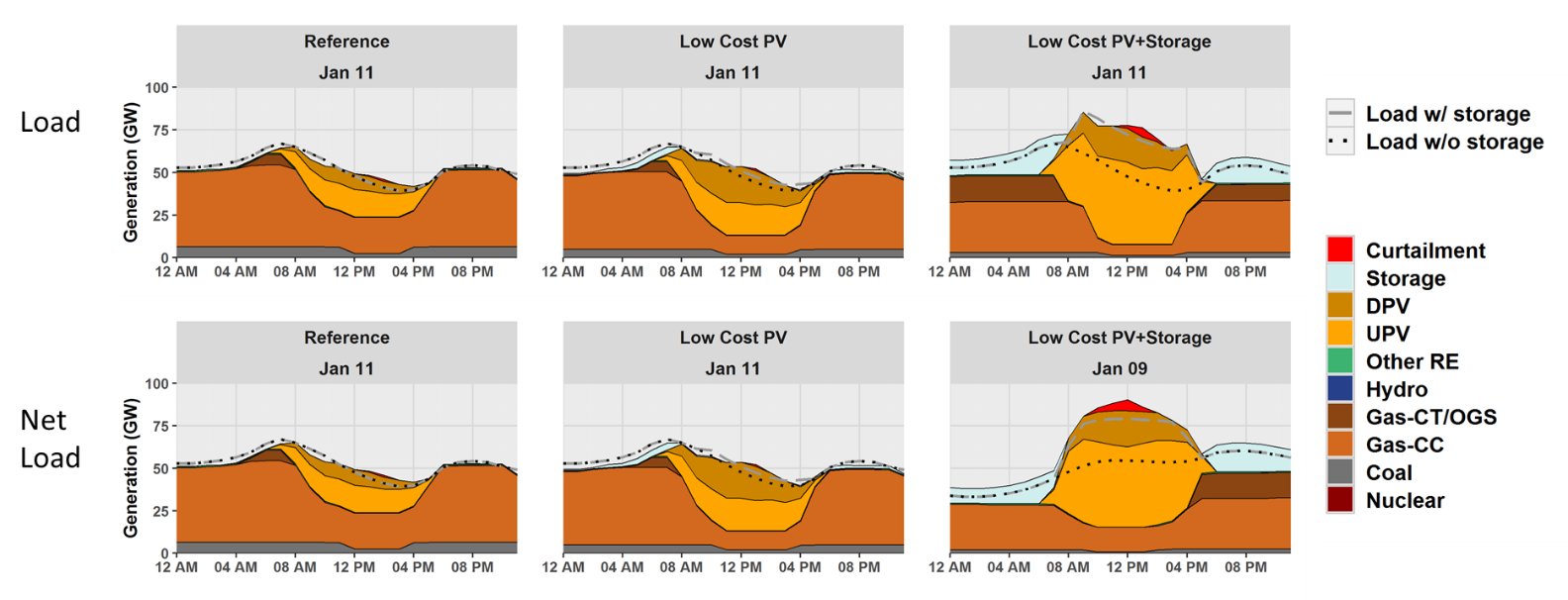


Figure S21. FRCC hourly dispatch for each scenario's day with peak load and net load hour, related to Figure 2 and Figure S2.


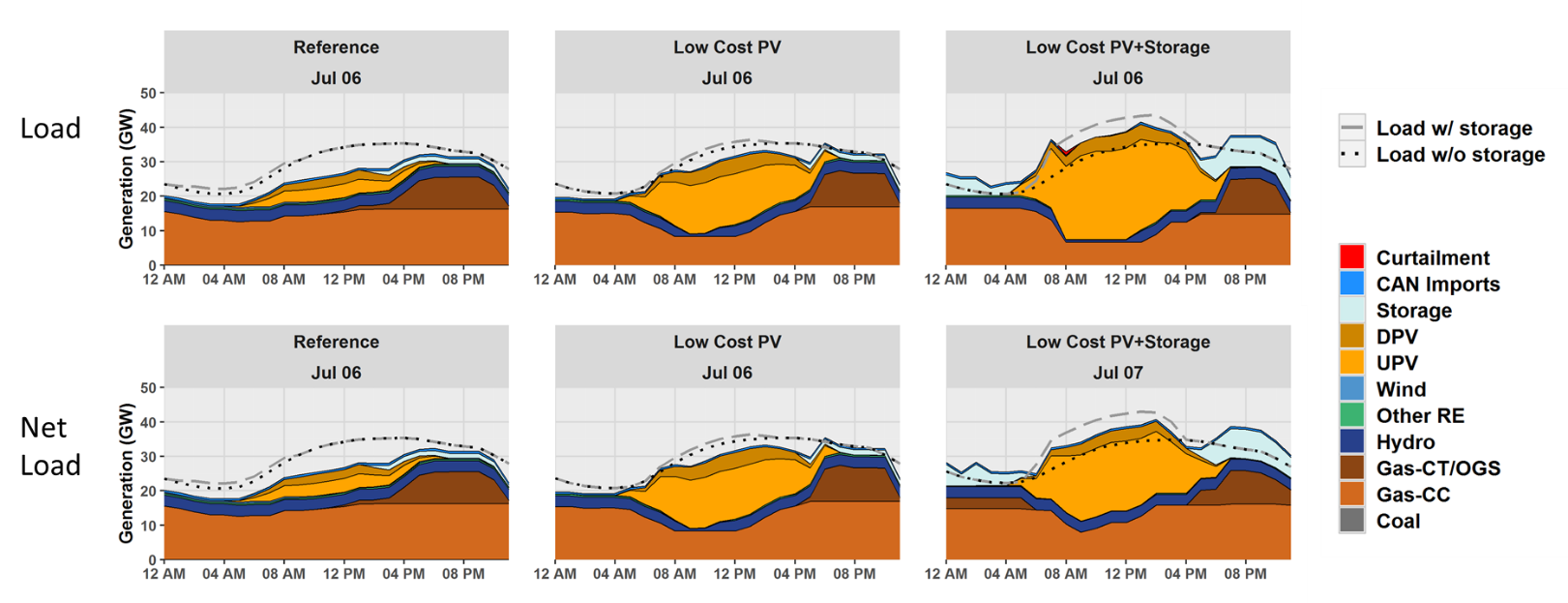


Figure S22. NYISO hourly dispatch for each scenario's day with peak load and net load hour, related to Figure 2 and Figure S2.


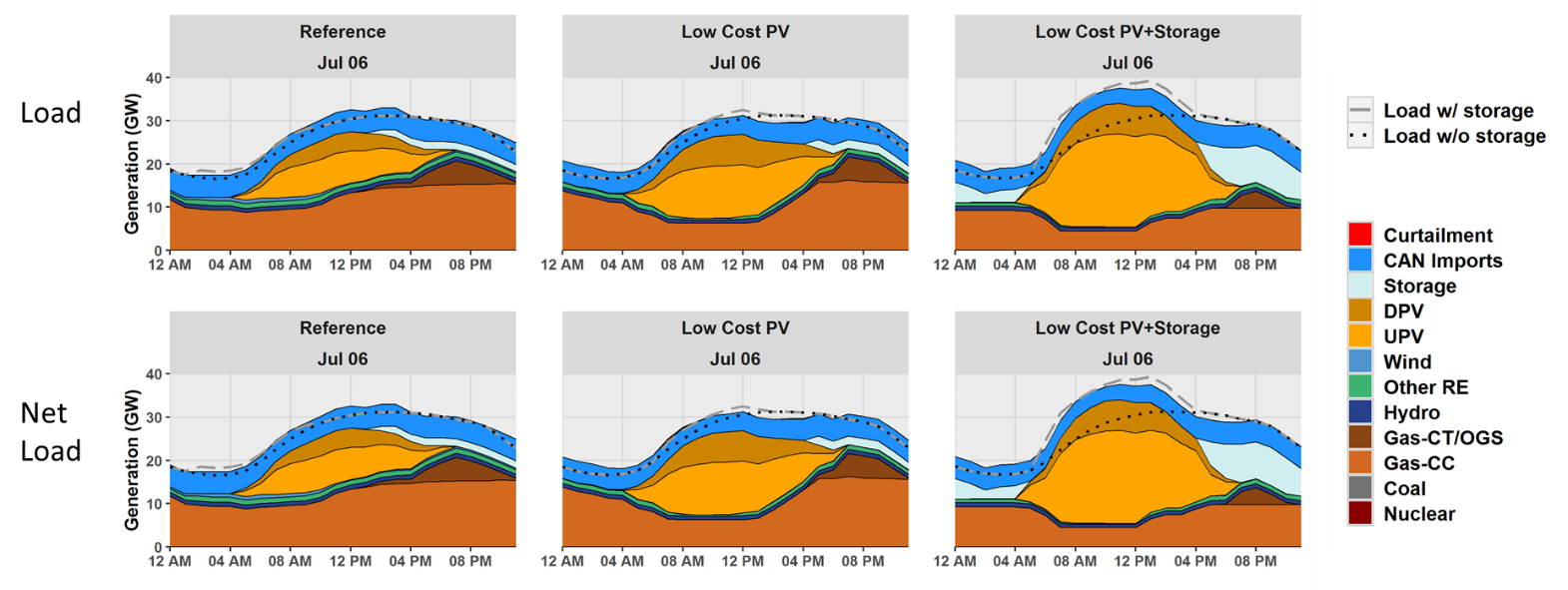


Figure S23. ISO-NE hourly dispatch for each scenario's day with peak load and net load hour, related to Figure 2 and Figure S2.


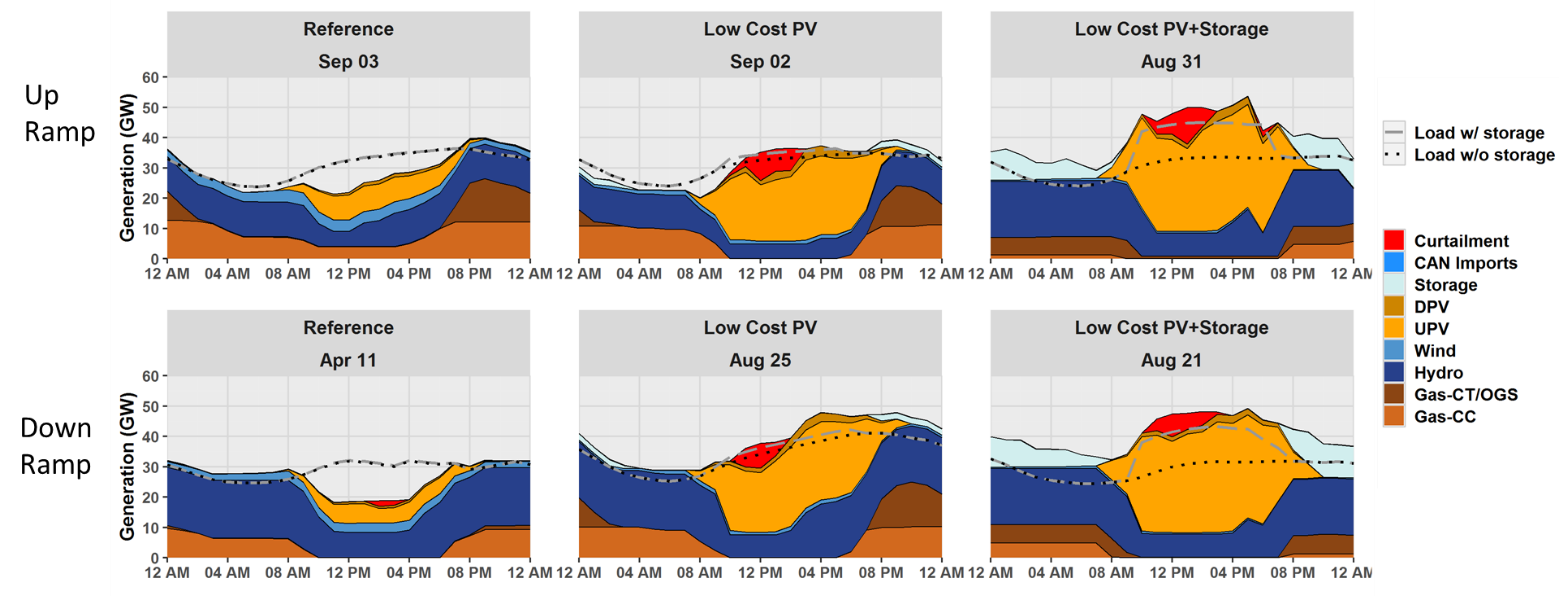


Figure S24. BPA hourly dispatch for each scenario's day of largest up and down 3-hour ramp, related to Figure 3 and Figure S2.


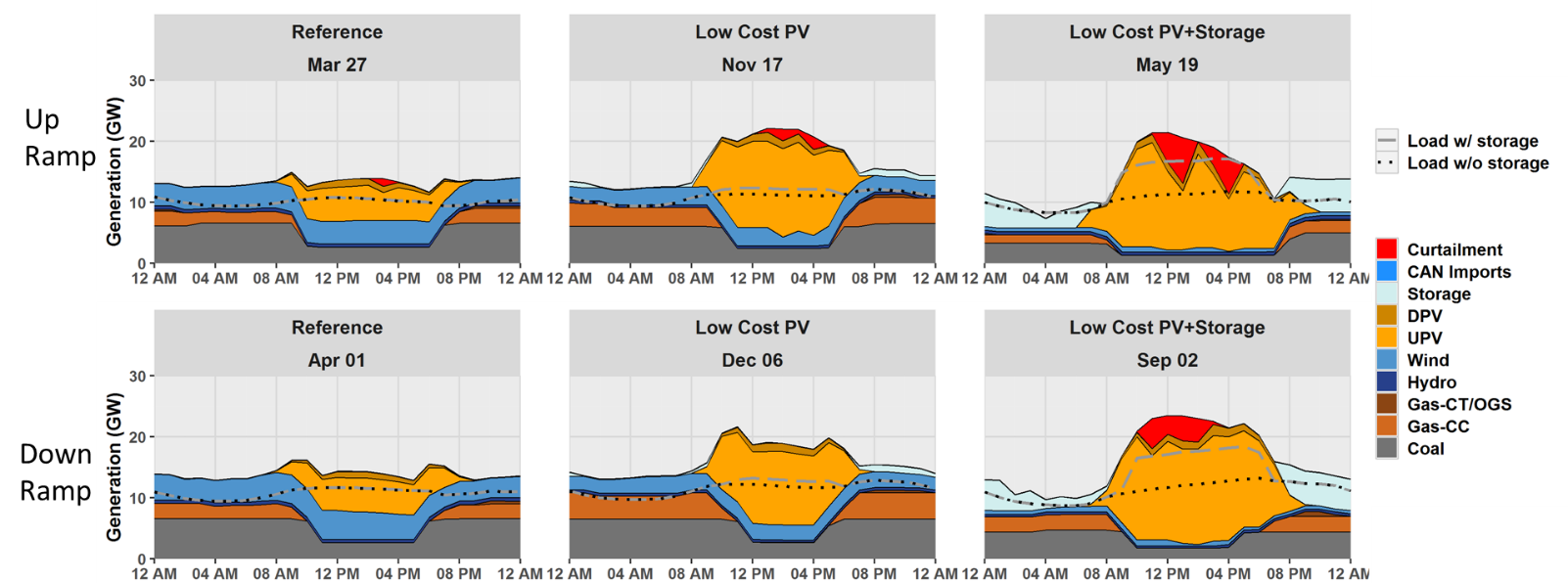


Figure S25. NWPP hourly dispatch for each scenario's day of largest up and down 3-hour ramp, related to Figure 3 and Figure S2.


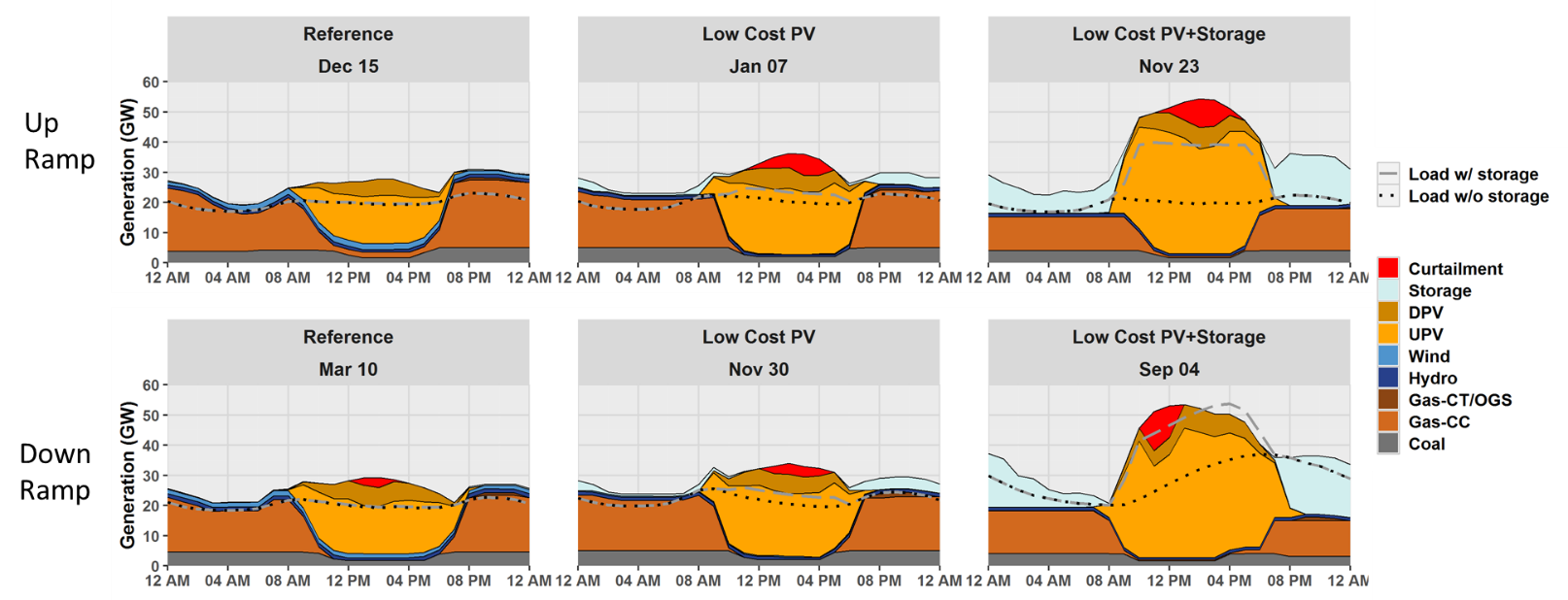


Figure S26. AZNM hourly dispatch for each scenario's day of largest up and down 3-hour ramp, related to Figure 3 and Figure S2.


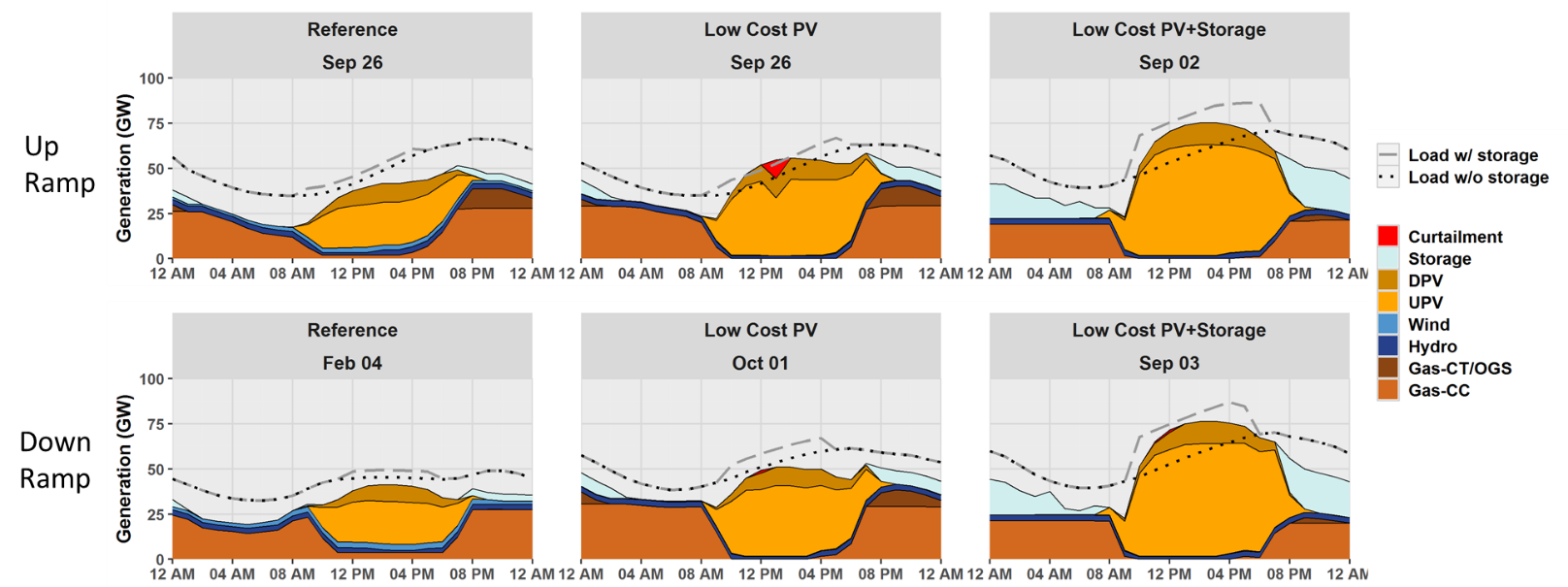


Figure S27. CAISO hourly dispatch for each scenario's day of largest up and down 3-hour ramp, related to Figure 3 and Figure S2.


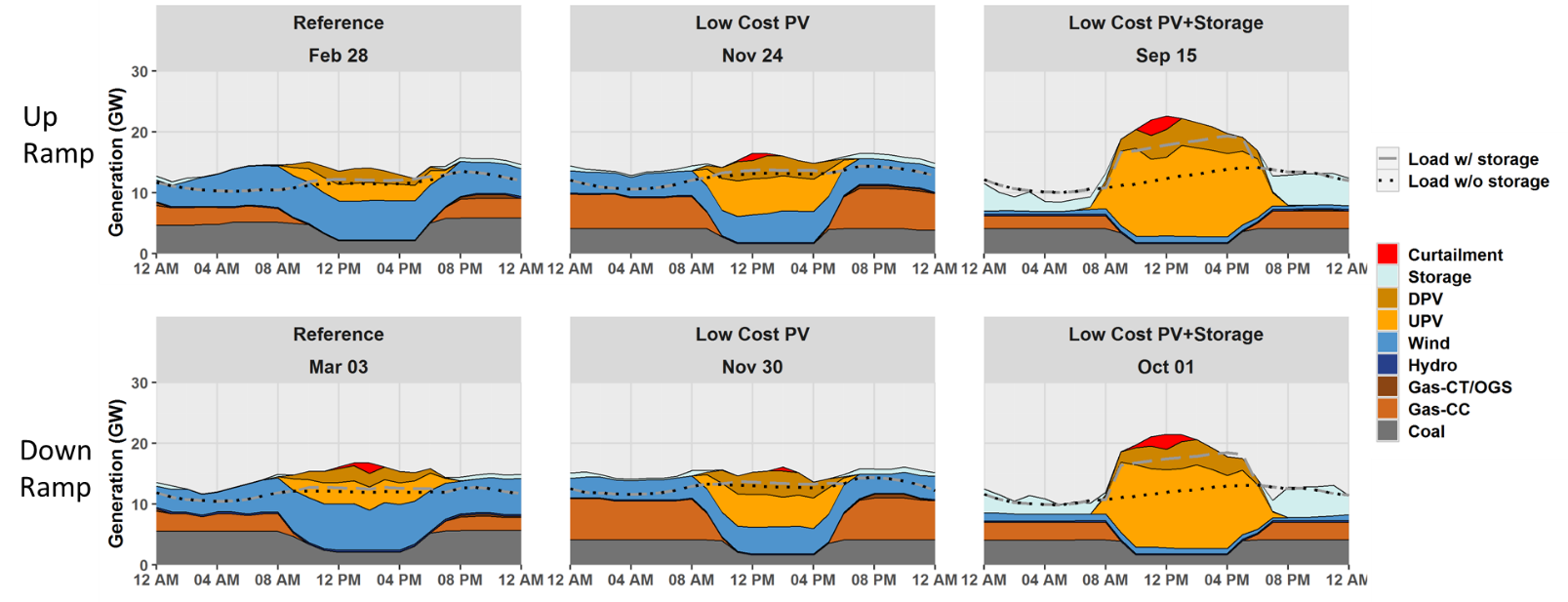


Figure S28. RMPP hourly dispatch for each scenario's day of largest up and down 3-hour ramp, related to Figure 3 and Figure S2.


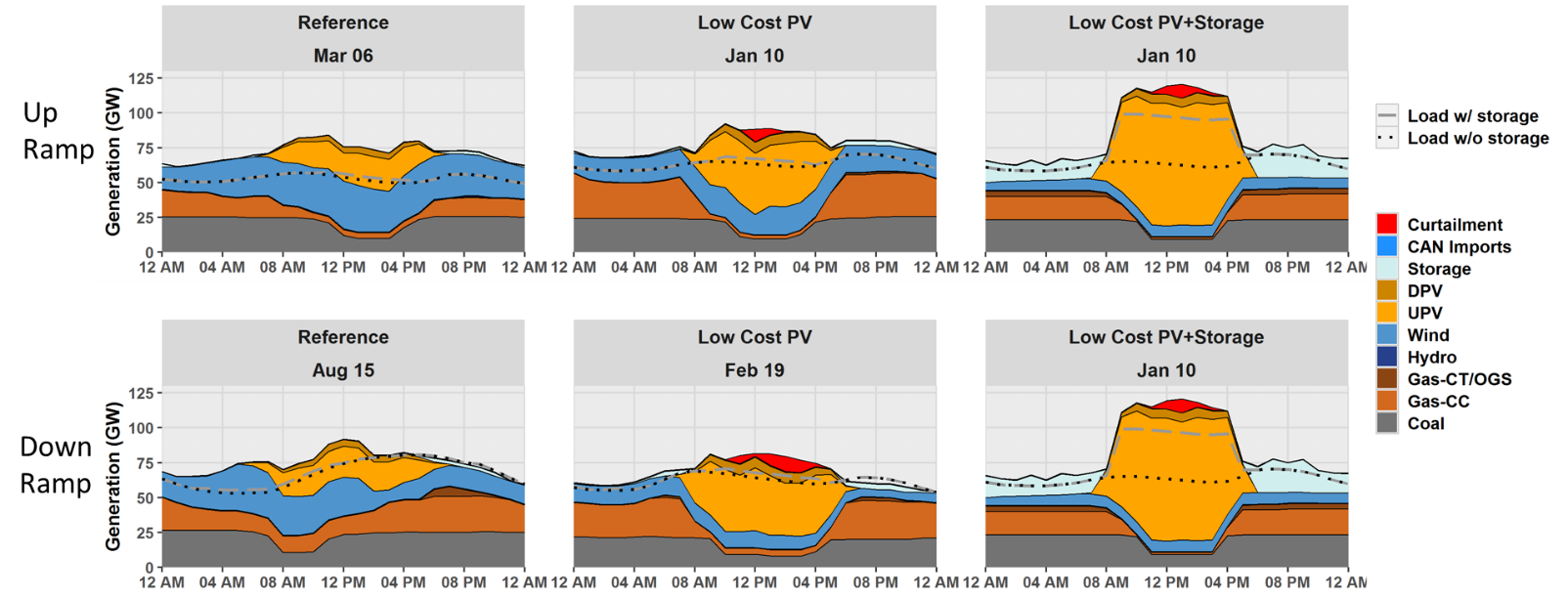


Figure S29. MISO-E hourly dispatch for each scenario's day of largest up and down 3-hour ramp, related to Figure 3 and Figure S2.


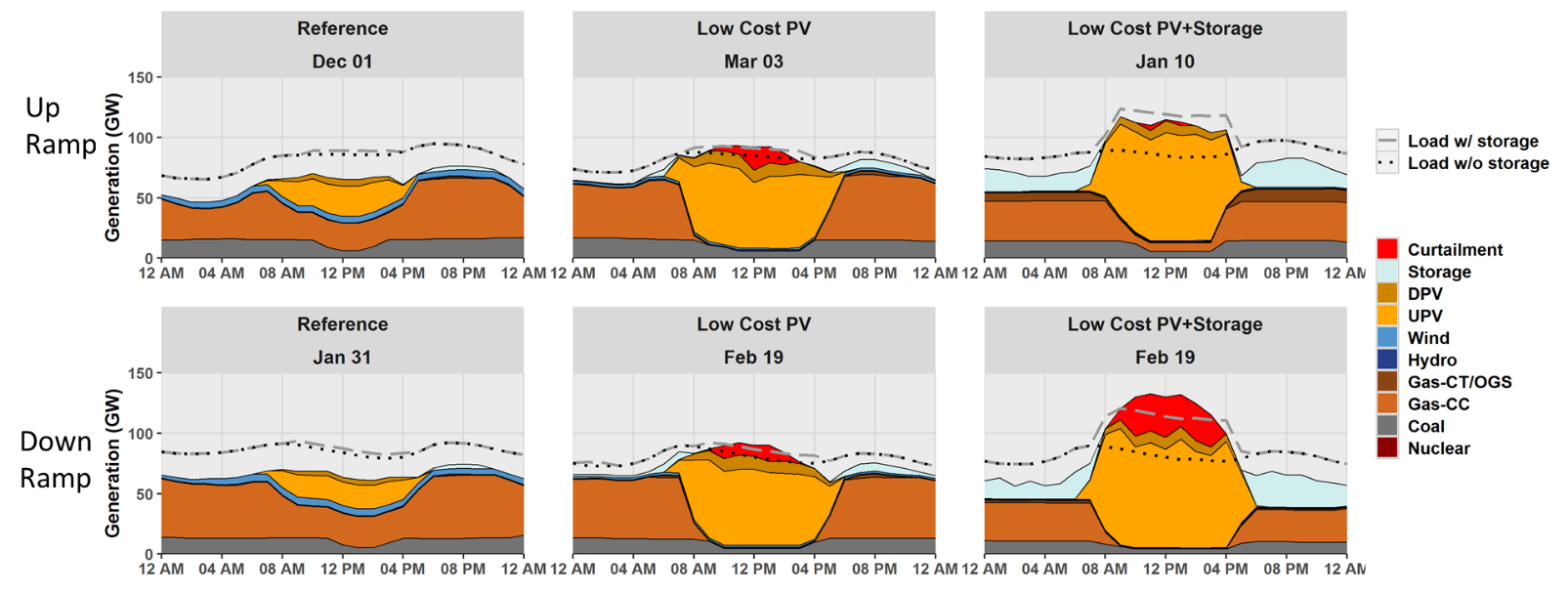


Figure S30. PJM-W hourly dispatch for each scenario's day of largest up and down 3-hour ramp, related to Figure 3 and Figure S2.


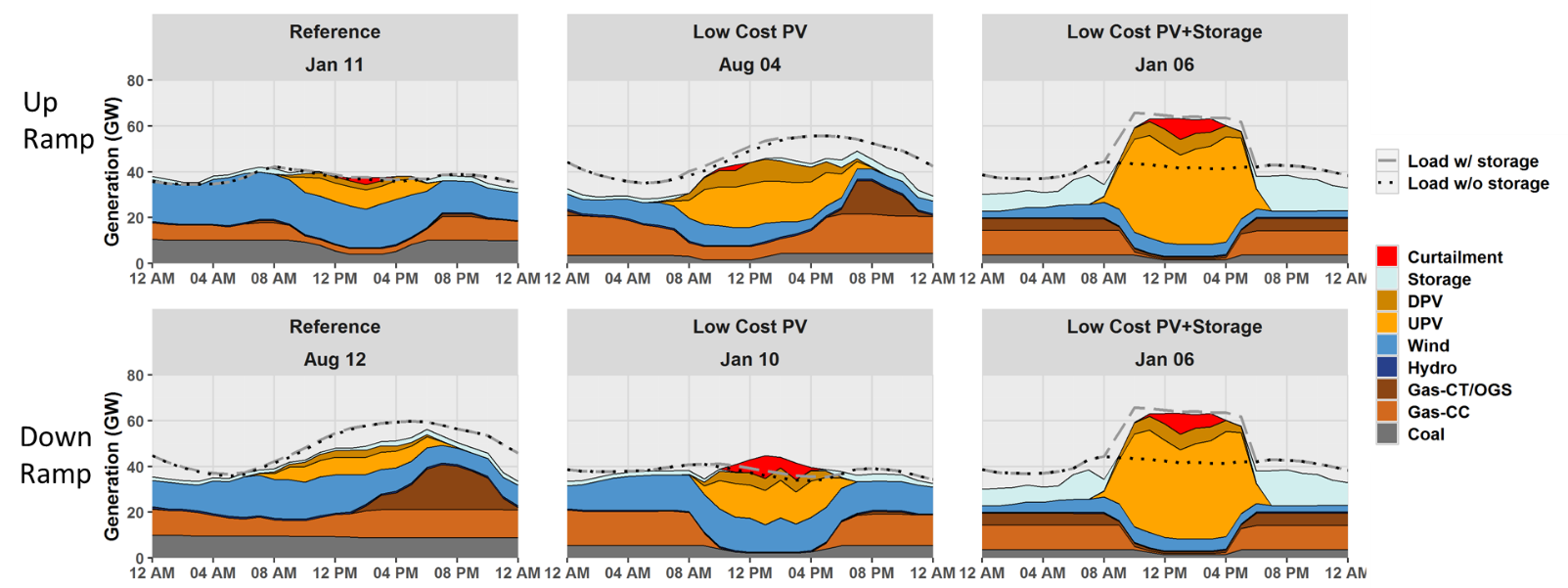


Figure S31. SPP hourly dispatch for each scenario's day of largest up and down 3-hour ramp, related to Figure 3 and Figure S2.


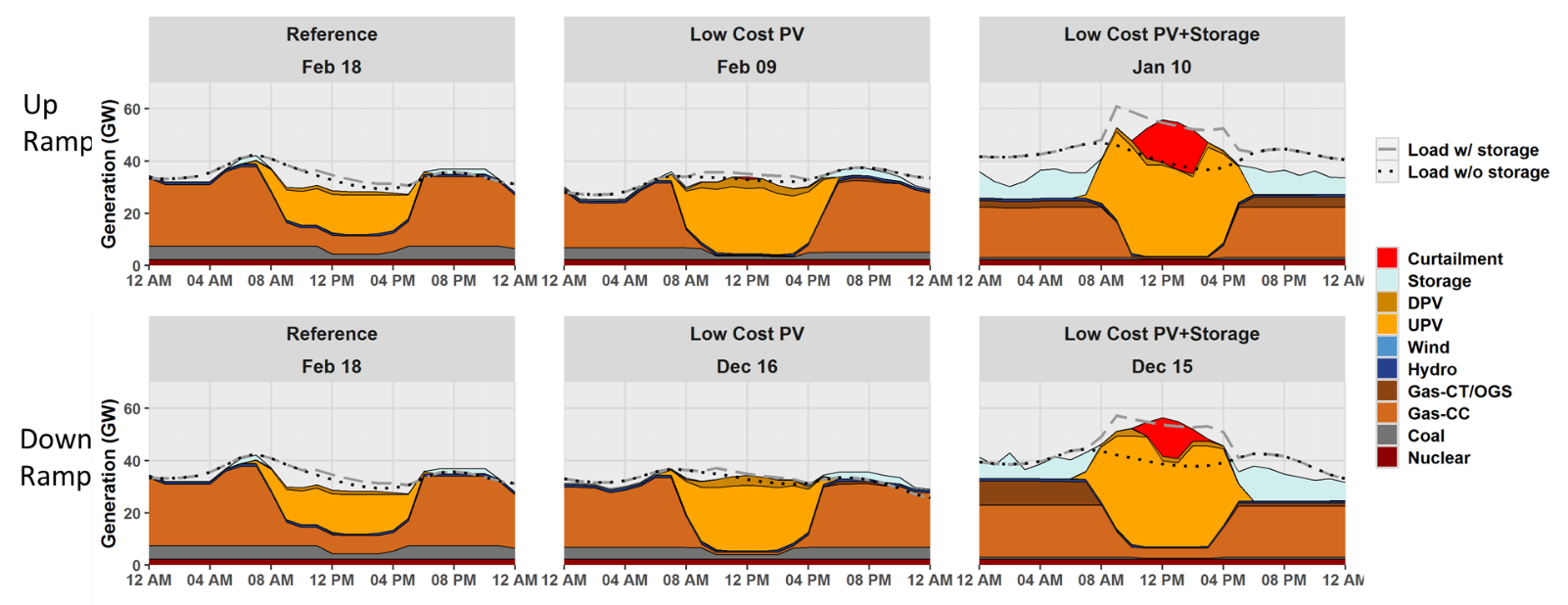


Figure S32. SE hourly dispatch for each scenario's day of largest up and down 3-hour ramp, related to Figure 3 and Figure S2.


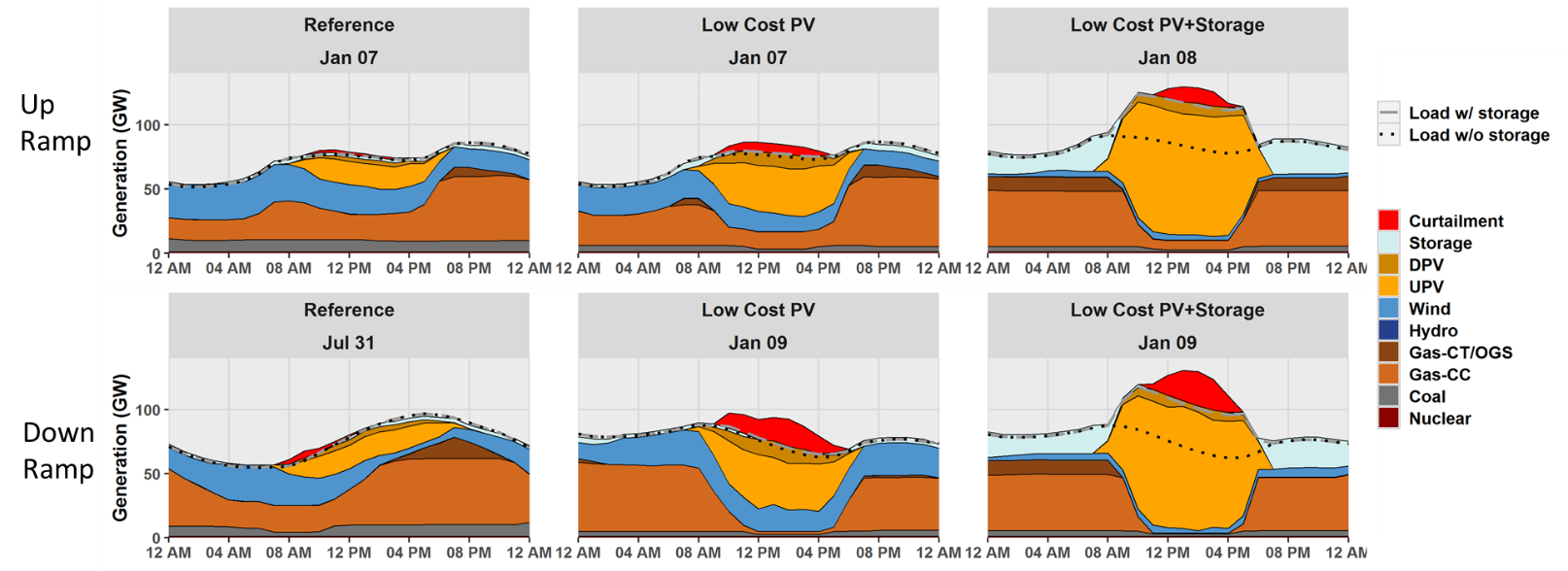


Figure S33. ERCOT hourly dispatch for each scenario's day of largest up and down 3-hour ramp, related to Figure 3 and Figure S2.


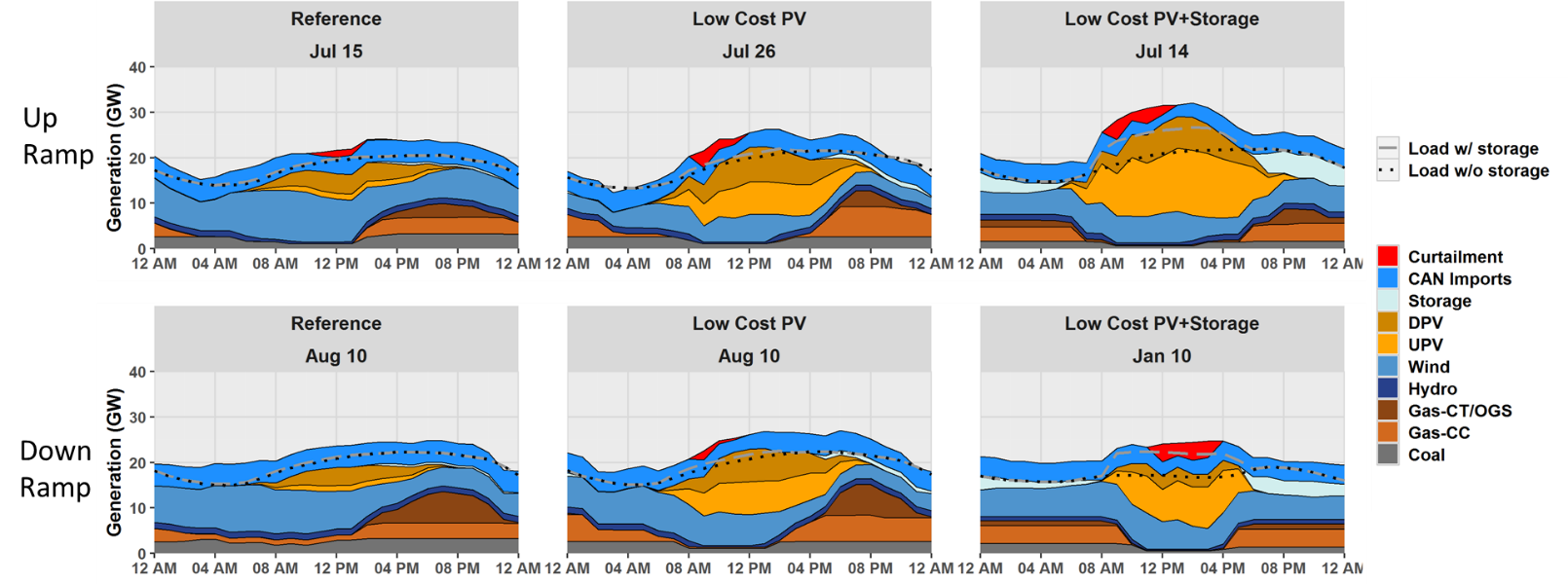


Figure S34. MISO-W hourly dispatch for each scenario's day of largest up and down 3-hour ramp, related to Figure 3 and Figure S2.


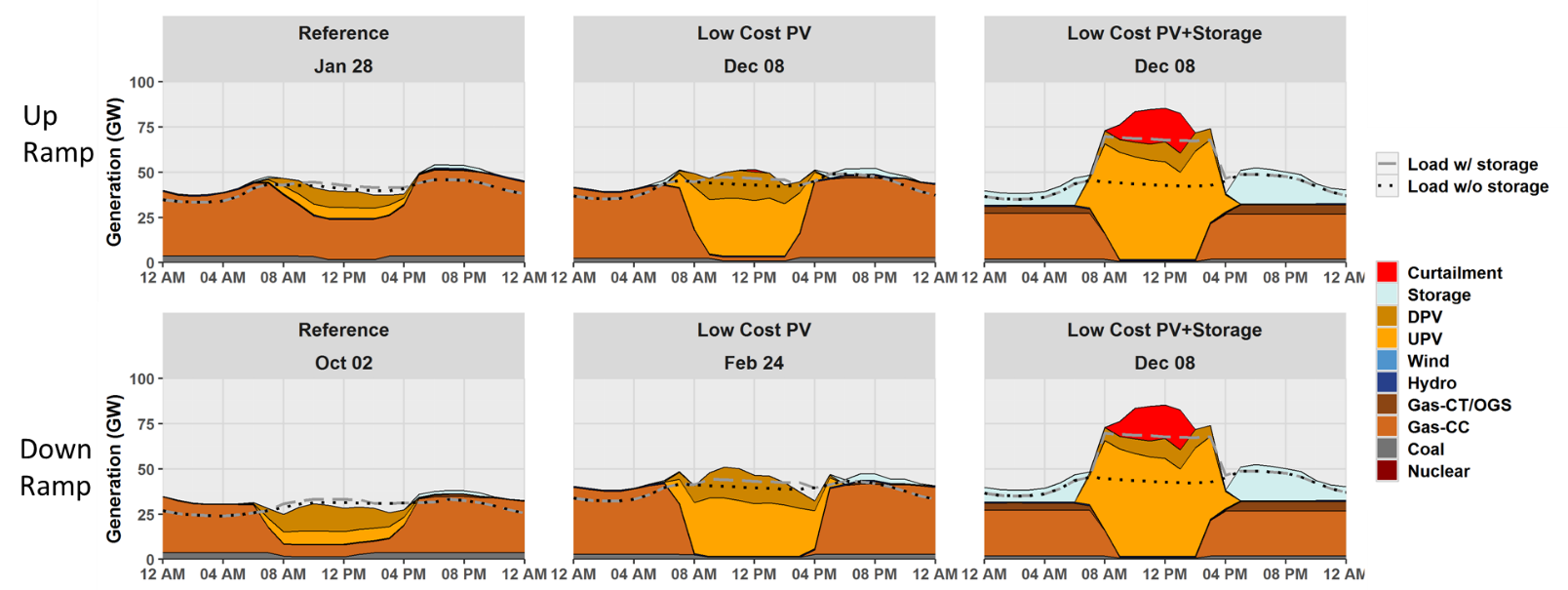


Figure S35. PJM-E hourly dispatch for each scenario's day of largest up and down 3-hour ramp, related to Figure 3 and Figure S2.


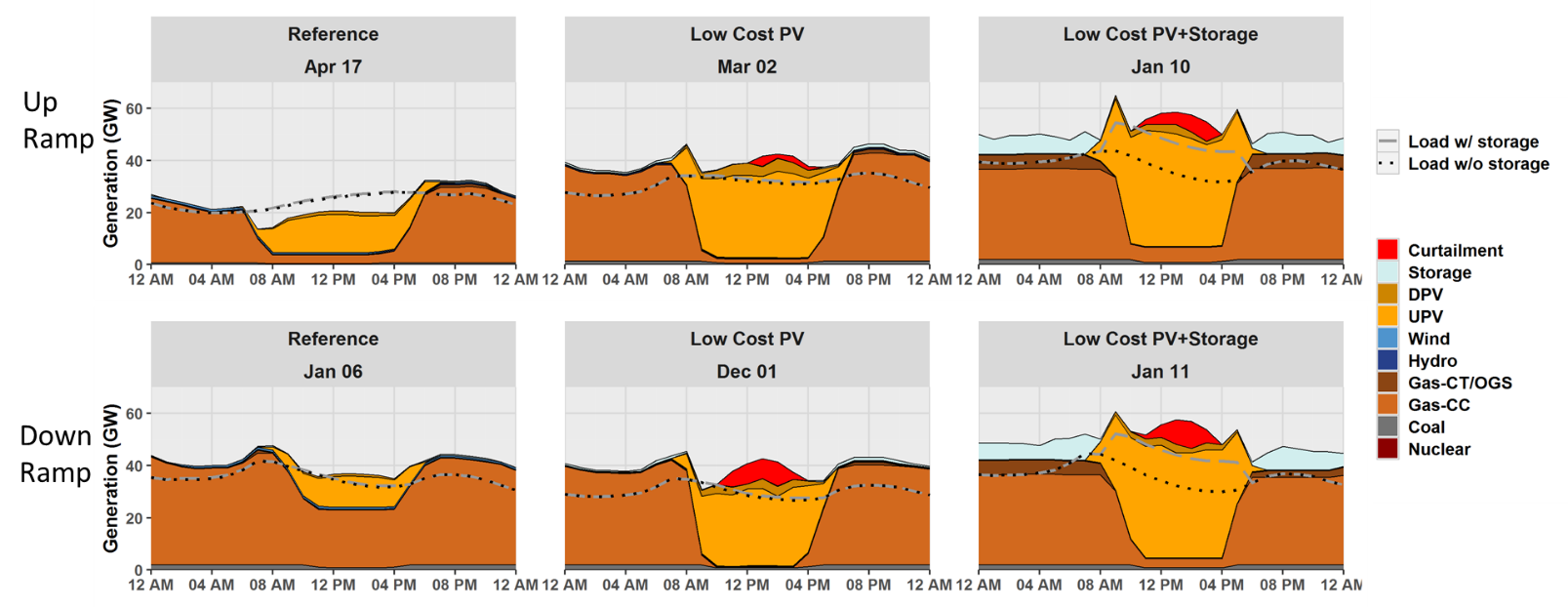


Figure S36. MISO-S hourly dispatch for each scenario's day of largest up and down 3-hour ramp, related to Figure 3 and Figure S2.


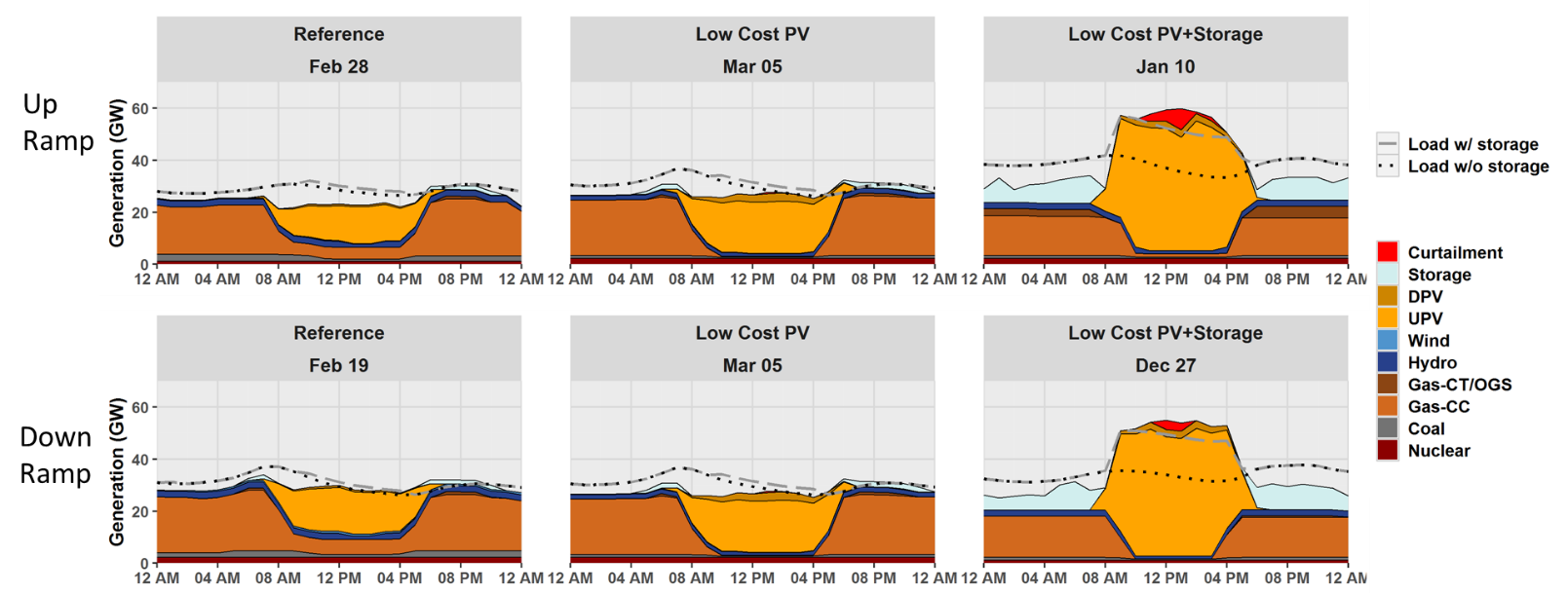


Figure S37. TVA hourly dispatch for each scenario's day of largest up and down 3-hour ramp, related to Figure 3 and Figure S2.


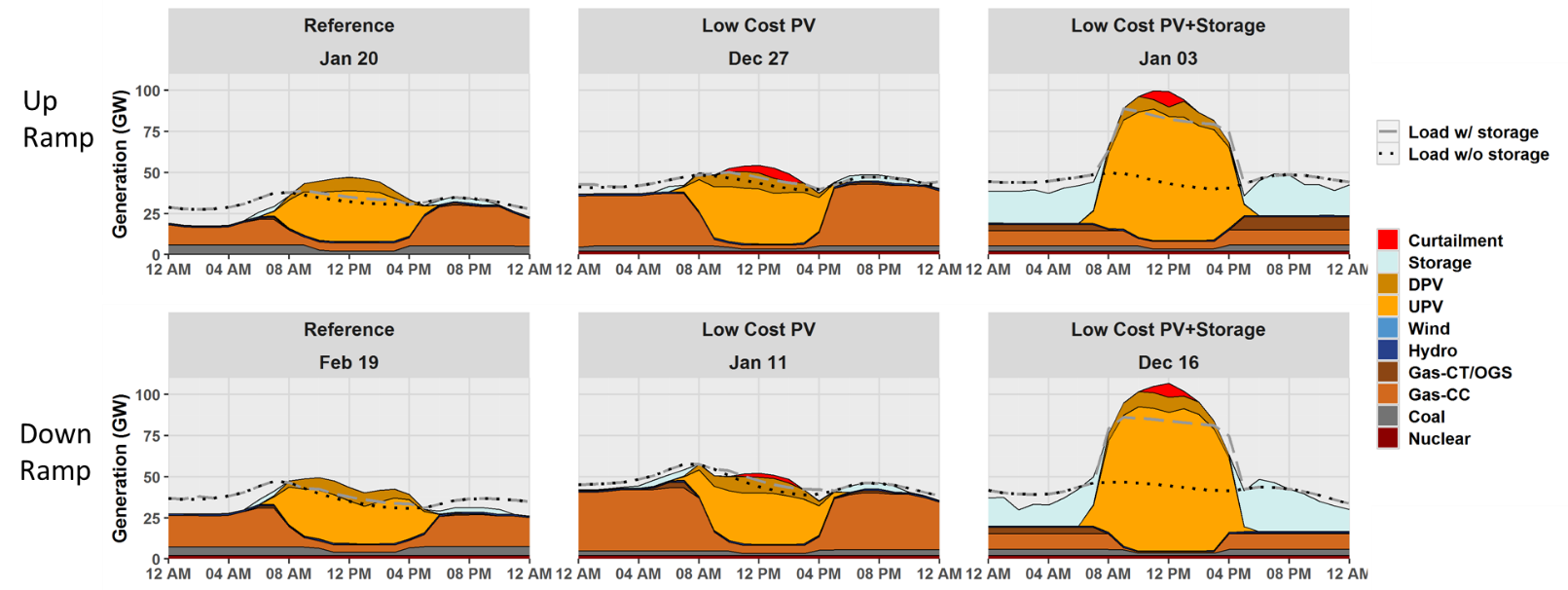


Figure S38. VACAR hourly dispatch for each scenario's day of largest up and down 3-hour ramp, related to Figure 3 and Figure S2.


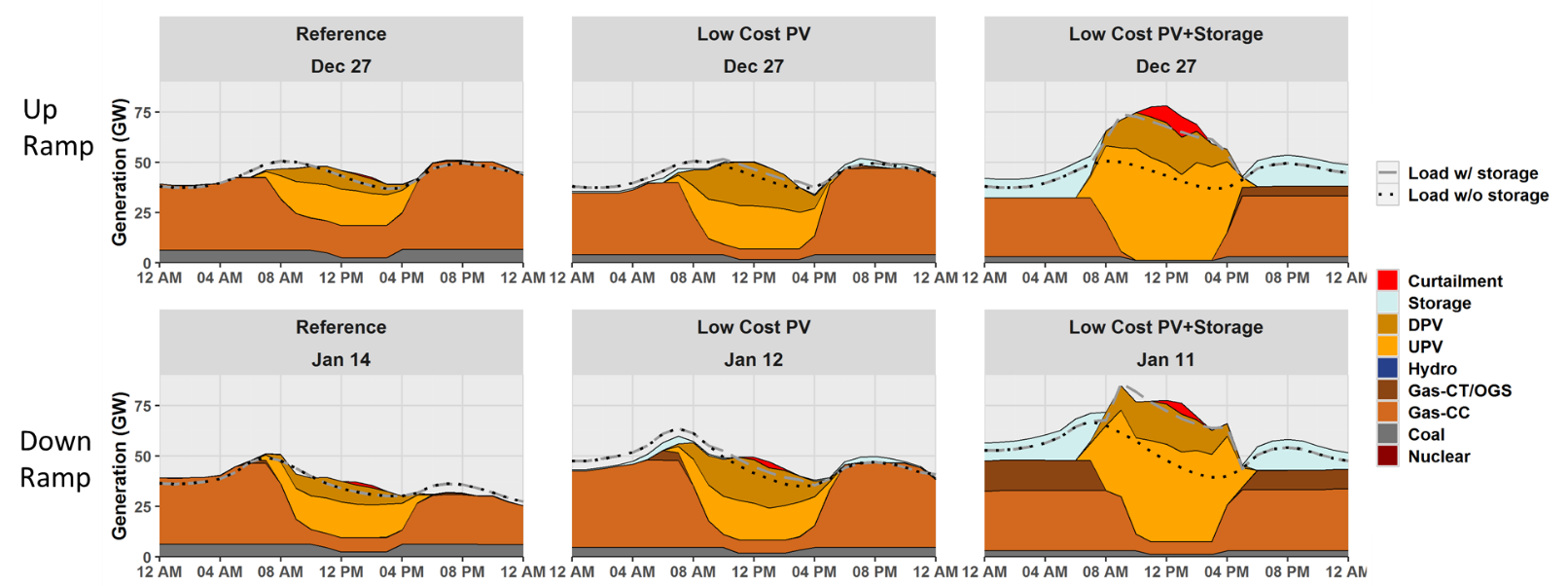


Figure S39. FRCC hourly dispatch for each scenario's day of largest up and down 3-hour ramp, related to Figure 3 and Figure S2.


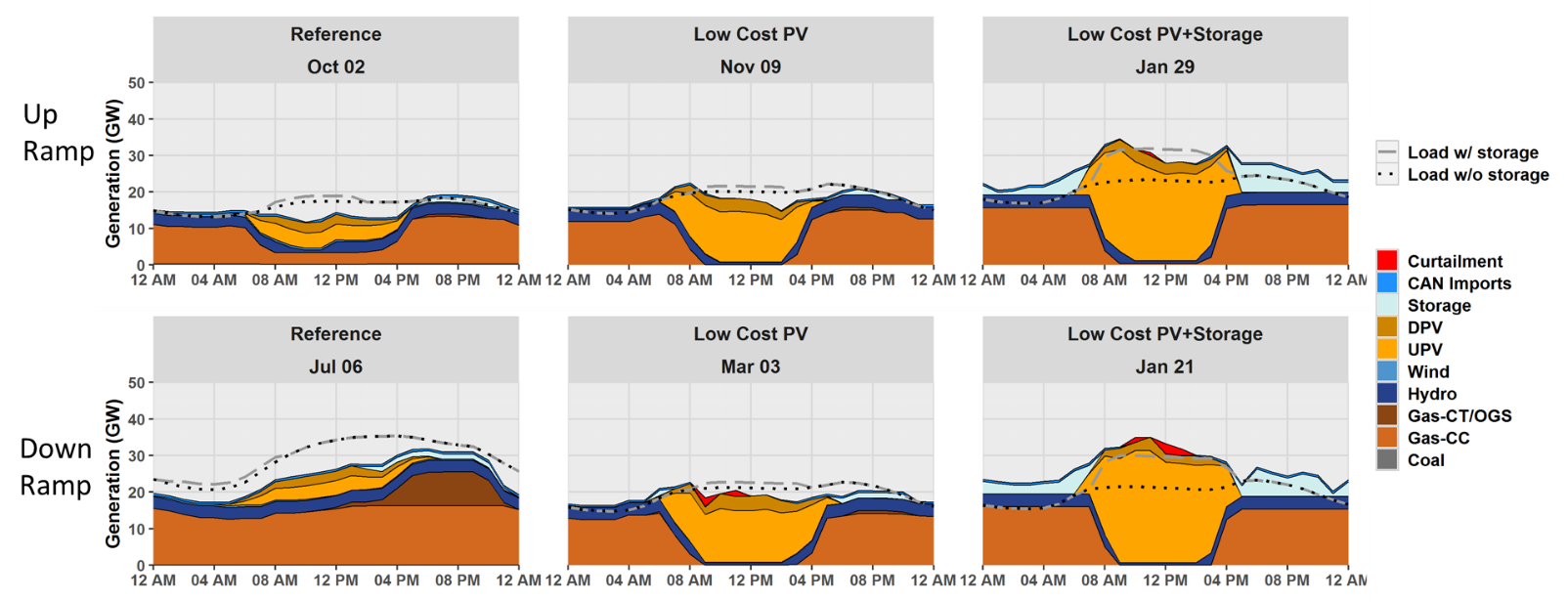


Figure S40. NYISO hourly dispatch for each scenario's day of largest up and down 3-hour ramp, related to Figure 3 and Figure S2.


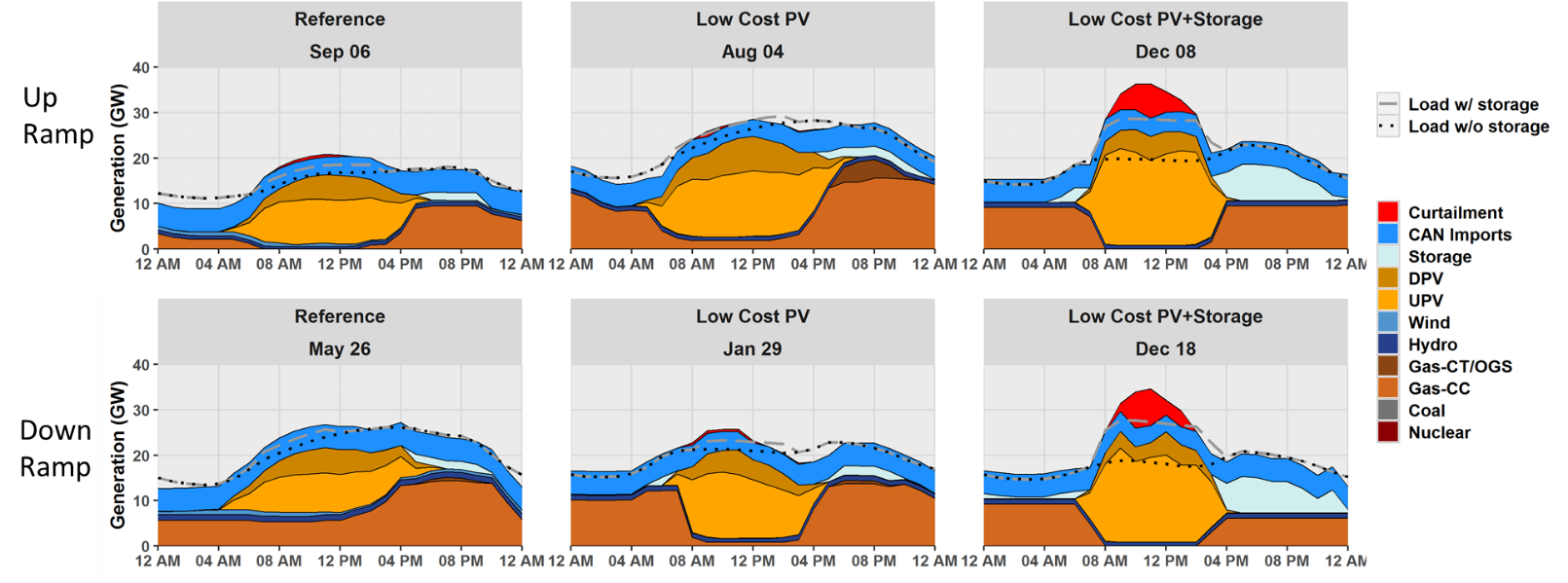


Figure S41. ISO-NE hourly dispatch for each scenario's day of largest up and down 3-hour ramp, related to Figure 3 and Figure S2.

Table S1. Summary of scenarios described in main text, related to all results (main text Figures 1-6).


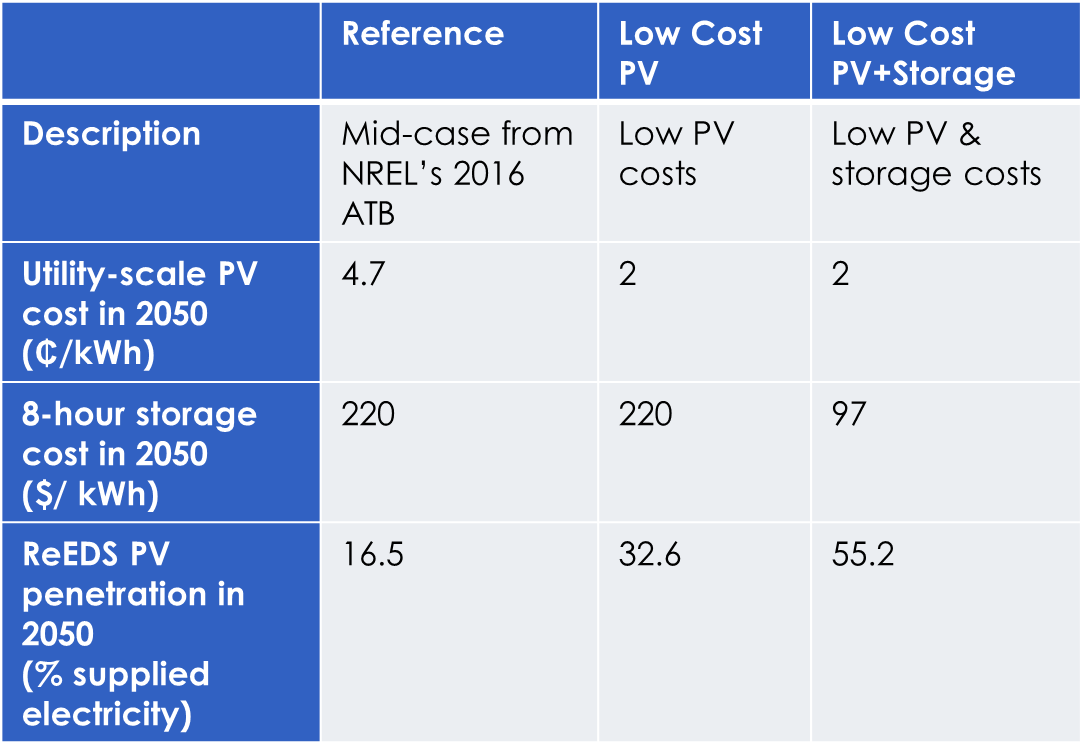


Table S2. Summary of natural gas price inputs to PLEXOS across the 134 regions, related to all results (main text Figures 1-6).


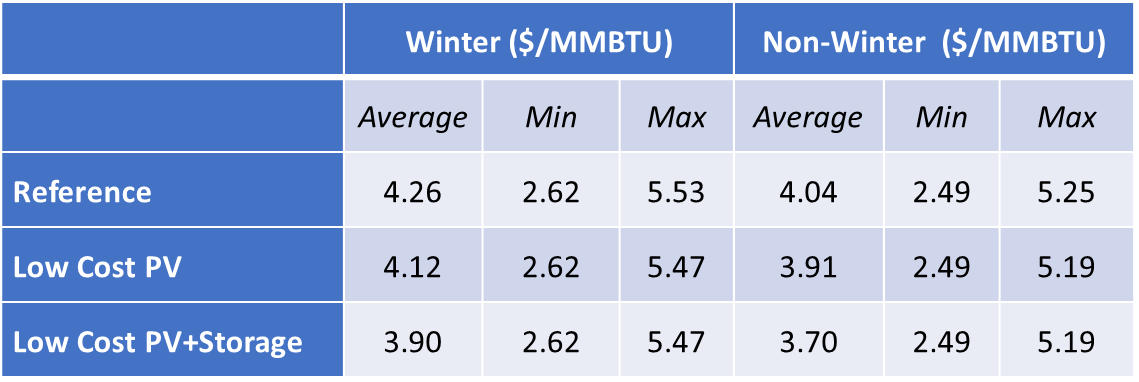


Table S3. LCGS parameters used for plants in PLEXOS. Notes: CC = Combined Cycle; CT = Combustion Turbine; IGCC=Integrated Gasification Combined Cycle; CCS = Carbon Capture and Storage. Related to all results (main text Figures 1-6).

| Category | Max. Capacity (MW) | Average Capacity (MW) | Min. Up Time (hours) | Min. Down Time (hours) | Forced Outage Rate (%) | Main-tenance Rate (%) | Mean Time to Repair (hours) | Start Cost  ($/MW) | Min. Stable Level (unitless) | Max. Ramp Up |
| --- | --- | --- | --- | --- | --- | --- | --- | --- | --- | --- |
| Natural Gas-CC | 944 | 320 | 8 | 4 | 3.28 | 6 | 52 | 83.7 | 0.55 | 0.0086 |
| Natural Gas-CT | 803 | 53 | 1 | 1 | 4.19 | 5 | 50 | 33.9 | 0.45 | 0.0534 |
| Nuclear | 1403.19 | 1239.94 | 48 | 48 | 4 | 6 | 298 | 116.6 | 1 | 0.0032 |
| Oil/Gas Steam | 803 | 53 | 1 | 1 | 3.63 | 11.57 | 51 | 33.9 | 0.45 | 0.0534 |
| Coal-IGCC | 856 | 302 | 168 | 48 | 4.29 | 12 | 37.5 | 155.8 | 0.4 | 0.0094 |
| Coal-CCS | 856 | 302 | 168 | 48 | 4.29 | 12 | 37.5 | 155.8 | 0.4 | 0.0094 |
| Coal-new | 856 | 302 | 168 | 48 | 4.29 | 10 | 37.5 | 155.8 | 0.4 | 0.0094 |
| Coal-old (Scrubbed) | 856 | 302 | 168 | 48 | 4.29 | 10 | 37.5 | 155.8 | 0.4 | 0.0094 |
| Coal-old (Unscrubbed) | 856 | 302 | 168 | 48 | 4.29 | 10 | 37.5 | 155.8 | 0.4 | 0.0094 |
| Coal-new (Co-fired) | 856 | 302 | 168 | 48 | 4.29 | 10 | 37.5 | 155.8 | 0.4 | 0.0094 |
| Coal-old (Co-fired) | 856 | 302 | 168 | 48 | 4.29 | 10 | 37.5 | 155.8 | 0.4 | 0.0094 |
| Biopower | 236.8 | 11.87 | 6 | 7 | 3.09 | 7.6 | 38 | 5.3 | 0.3 | 0.1389 |
| Landfill Gas | 236.8 | 11.87 | 6 | 7 | 3.09 | 5 | 38 | 5.3 | 0.3 | 0.1389 |
| Geothermal | 197 | 26 | 16 | 6 | 3.09 | 2.41 | 24 | 0.0 | 0.5 | 0.0342 |

Table S4. Low Cost PV+Storage scenario dropped load (initial run) and installed storage capacity resulting from modified storage capacity value (final run), related to all results (main text Figures 1-6).


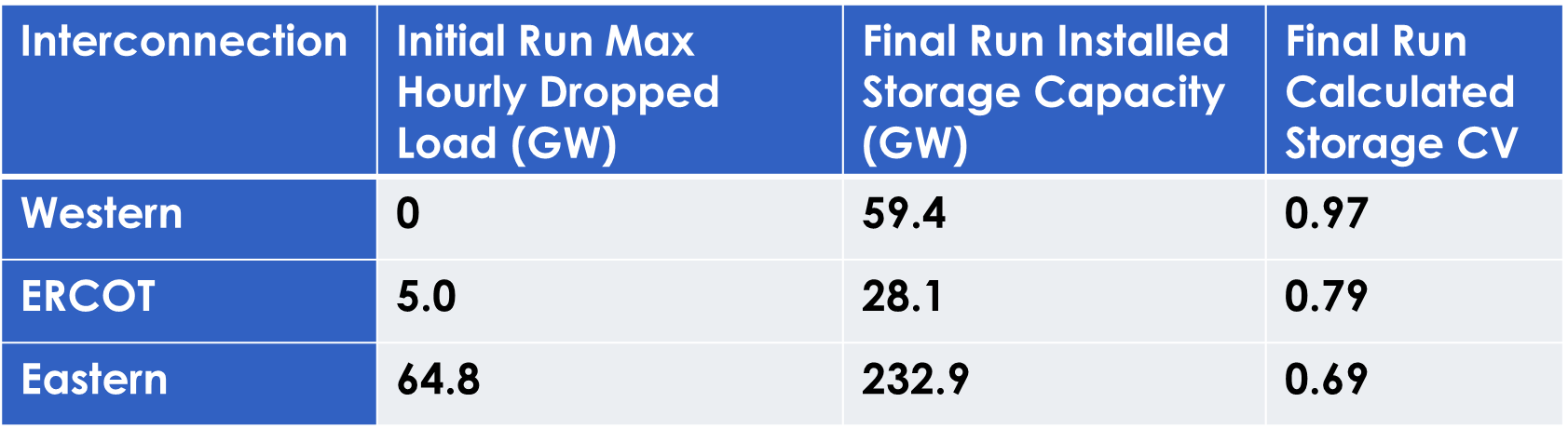


Table S5. Nationwide capacity factor and penetration of storage and PV during peak load and net load hours by scenario. Peak load occurs at 5 p.m. on August 4 for all scenarios. Peak net load occurs at 8 p.m. on August 10 for the Reference and Lo- Cost PV scenarios and at midnight on December 14 for the Low Cost PV+Storage scenario. The peak net load values correspond to the nationwide dispatch plots shown in Figure 2 of the main text.


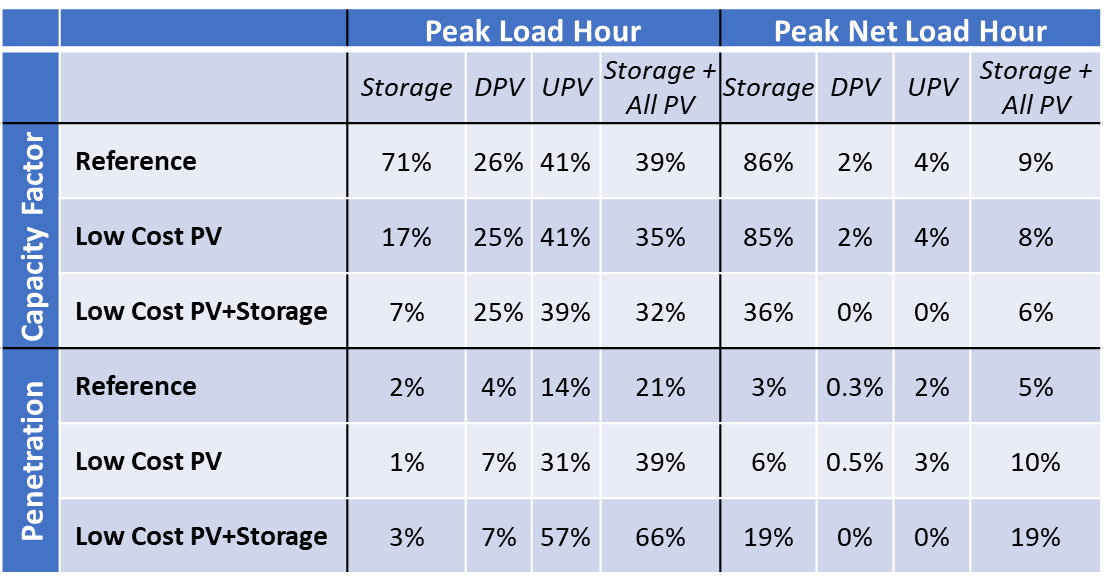


# References

Auffhammer, M., Baylis, P., Hausman, C.H., 2017. Climate change is projected to have severe impacts on the frequency and intensity of peak electricity demand across the United States. Proc. Natl. Acad. Sci. 114, 1886–1891. https://doi.org/10.1073/pnas.1613193114

Barrows, C., Hummon, M., Jones, W., Hale, E., 2014. Time Domain Partitioning of Electricity Production Cost Simulations (No. NREL/TP-6A20-60969). National Renewable Energy Lab. (NREL), Golden, CO (United States). https://doi.org/10.2172/1123223

Becker, S., Frew, B.A., Andresen, G.B., Jacobson, M.Z., Schramm, S., Greiner, M., 2015. Renewable build-up pathways for the US: Generation costs are not system costs. Energy 81, 437–445. https://doi.org/10.1016/j.energy.2014.12.056

Becker, S., Frew, B.A., Andresen, G.B., Zeyer, T., Schramm, S., Greiner, M., Jacobson, M.Z., 2014. Features of a fully renewable US electricity system: Optimized mixes of wind and solar PV and transmission grid extensions. Energy 72, 443–458. https://doi.org/10.1016/j.energy.2014.05.067

Brinkman, G., Jorgenson, J., Ehlen, A., Caldwell, J., 2016. Low Carbon Grid Study: Analysis of a 50% Emission Reduction in California (No. NREL/TP-6A20-64884). National Renewable Energy Laboratory, Golden, CO.

Bussar, C., Moos, M., Alvarez, R., Wolf, P., Thien, T., Chen, H., Cai, Z., Leuthold, M., Sauer, D.U., Moser, A., 2014. Optimal Allocation and Capacity of Energy Storage Systems in a Future European Power System with 100% Renewable Energy Generation. Energy Procedia, 8th International Renewable Energy Storage Conference and Exhibition (IRES 2013) 46, 40–47. https://doi.org/10.1016/j.egypro.2014.01.156

Cochran, J., Mai, T., Bazilian, M., 2014. Meta-analysis of high penetration renewable energy scenarios. Renew. Sustain. Energy Rev. 29, 246–253. https://doi.org/10.1016/j.rser.2013.08.089

Cole, W., Frazier, A.W., Das, P., Mai, T., Donohoo-Vallett, P., 2018a. 2018 Standard Scenarios Report: A U.S. Electricity Sector Outlook (No. NREL/TP-6A20-71913). National Renewable Energy Laboratory, Golden, CO. https://doi.org/10.2172/1481848

Cole, W., Frew, B., Gagnon, P., Reimers, A., Zuboy, J., Margolis, R., 2018b. Envisioning a low-cost solar future: Exploring the potential impact of Achieving the SunShot 2030 targets for photovoltaics. Energy Oxf. 155. https://doi.org/10.1016/j.energy.2018.04.166

Cole, W., Frew, B., Mai, T., Sun, Y., Bistline, J., Blanford, G., 2017. Variable Renewable Energy in Long-Term Planning Models: A Multi-Model Perspective (No. NREL/TP-6A20-70528). EERE, NREL, EIA, EPA ...

Connolly, D., Lund, H., Mathiesen, B.V., 2016. Smart Energy Europe: The technical and economic impact of one potential 100% renewable energy scenario for the European Union. Renew. Sustain. Energy Rev. 60, 1634–1653. https://doi.org/10.1016/j.rser.2016.02.025

Denholm, P., Margolis, R., 2018. The Potential for Energy Storage to Provide Peaking Capacity in California Under Increased Penetration of Solar Photovoltaics. (Technical Report No. NREL/TP-6A20-70905). National Renewable Energy Laboratory, Golden, CO.

Denholm, P.L. (ORCID:0000000171284643), Nunemaker, J., Cole, W.J., Gagnon, P.J., 2019. The Potential for Battery Energy Storage to Provide Peaking Capacity in the United States (No. NREL/TP-6A20-74184). National Renewable Energy Lab. (NREL), Golden, CO (United States). https://doi.org/10.2172/1530173

Department of Energy Office of Electricity Delivery and Energy Reliability, n.d. DOE OE Energy Storage Database [WWW Document]. URL https://www.energystorageexchange.org/ (accessed 1.15.19).

DOE, 2015. Wind Vision: A New Era for Wind Power in the United States (Technical Report No. DOE/GO-102015-4557). U. S. Department of Energy, Washington, D.C.

EIA, 2016. Annual Energy Outlook 2016 (No. DOE/EIA-0383(2016)). U.S. DOE Energy Information Administration, Washington, D.C.

Electric Power Research Institute (EPRI), 2017. US-REGEN Model Documentation (No. EPRI Technical Update #3002010956). EPRI, Palo Alto, CA.

Energy Exemplar, n.d. PLEXOS Simulation Software.

Eurek, K., Cole, W., Bielen, D.A., Blair, N., Cohen, S., Frew, B., Ho, J., Krishnan, V., Mai, T., Steinberg, D., 2016. Regional Energy Deployment System (ReEDS) Model Documentation: Version 2016 (No. NREL/TP-6A20-67067). National Renewable Energy Laboratory, Golden, CO.

Frew, B.A., Becker, S., Dvorak, M.J., Andresen, G.B., Jacobson, M.Z., 2016. Flexibility mechanisms and pathways to a highly renewable US electricity future. Energy 101, 65–78. https://doi.org/10.1016/j.energy.2016.01.079

Frew, B.A., Cole, W.J., Vincent, N.M. (ORCID:0000000221442954), Reimers, A., Margolis, R.M., 2018. Impact of Dynamic Storage Capacity Valuation in Capacity Expansion Models: Preprint (No. NREL/CP-6A20-71462). National Renewable Energy Lab. (NREL), Golden, CO (United States).

G. Blanford, J. Bistline, D. Young, J. Merrick, 2016. Simulating Annual Variation in Load, Wind, and Solar by Representative Hour Selection (No. 3002008653). EPRI, Palo Alto, CA.

Habte, A., Sengupta, M., Lopez, A., 2017. Evaluation of the National Solar Radiation Database (NSRDB): 1998-2015 (No. NREL/TP-5D00-67722). National Renewable Energy Lab. (NREL), Golden, CO (United States). https://doi.org/10.2172/1351858

Lew, D., Brinkman, G., Ibanez, E., Hodge, B.M., Hummon, M., Florita, A., Heaney, M., 2013. The Western Wind and Solar Integration Study Phase 2 (No. NREL/TP-5500-55588). National Renewable Energy Laboratory (NREL), Golden, CO.

Loutan, C., Klauer, P., Chowdhury, S., Hall, S., Morjaria, M., Chadliev, V., Milam, N., Milan, C., Gevorgian, V., 2017. Demonstration of Essential Reliability Services by a 300-MW Solar Photovoltaic Power Plant (No. NREL/TP-5D00-67799). National Renewable Energy Lab. (NREL), Golden, CO (United States). https://doi.org/10.2172/1349211

Mai, T., Barrows, C., Lopez, A., Hale, E., Dyson, M., Eurek, K., 2015. Implications of Model Structure and Detail for Utility Planning: Scenario Case Studies Using the Resource Planning Model (No. NREL/TP--6A20-63972). National Renewable Energy Lab. (NREL), Golden, CO (United States).

Mai, T., Mulcahy, D., Hand, M.M., Baldwin, S.F., 2014. Envisioning a renewable electricity future for the United States. Energy 65, 374–386. https://doi.org/10.1016/j.energy.2013.11.029

Mai, T.T., Jadun, P., Logan, J.S., McMillan, C.A., Muratori, M. (ORCID:0000000316886742), Steinberg, D.C. (ORCID:0000000317692261), Vimmerstedt, L.J., Haley, B., Jones, R., Nelson, B., 2018. Electrification Futures Study: Scenarios of Electric Technology Adoption and Power Consumption for the United States (No. NREL/TP-6A20-71500). National Renewable Energy Lab. (NREL), Golden, CO (United States). https://doi.org/10.2172/1459351

Martinek, J., Jorgenson, J., Mehos, M., Denholm, P., 2018. A comparison of price-taker and production cost models for determining system value, revenue, and scheduling of concentrating solar power plants. Appl. Energy 231, 854–865. https://doi.org/10.1016/j.apenergy.2018.09.136

Mathiesen, B.V., Lund, H., Karlsson, K., 2011. 100% Renewable energy systems, climate mitigation and economic growth. Appl. Energy, The 5th Dubrovnik Conference on Sustainable Development of Energy, Water and Environment Systems, held in Dubrovnik September/October 2009 88, 488–501. https://doi.org/10.1016/j.apenergy.2010.03.001

Pfenninger, S., 2017. Dealing with multiple decades of hourly wind and PV time series in energy models: A comparison of methods to reduce time resolution and the planning implications of inter-annual variability. Appl. Energy 197, 1–13. https://doi.org/10.1016/j.apenergy.2017.03.051

RTS-GMLC: Reliability Test System - Grid Modernization Lab Consortium [WWW Document], 2018. URL https://github.com/ GridMod/RTS-GMLC

Sigrin, B., Gleason, M., Preus, R., Baring-Gould, I., Margolis, R., 2016. The Distributed Generation Market Demand Model (dGen): Documentation (No. NREL/TP-6A20-65231). National Renewable Energy Laboratory, Golden, CO.

Sioshansi, R., Madaeni, S.H., Denholm, P., 2014. A Dynamic Programming Approach to Estimate the Capacity Value of Energy Storage. IEEE Trans. Power Syst. 29, 395–403. https://doi.org/10.1109/TPWRS.2013.2279839

U.S. Energy Information Administration (EIA), 2014. The Electricity Market Module of the National Energy Modeling System: Model Documentation 2014. Washington, D.C.

WECC, 2013. 2013 Interconnection-wide Plan Tools and Models.

1. Renewable energy plants are further distinguished by resource classes, such that there is a single model plant for each class in each region. Coal technologies are also subdivided into as many as four heat rate bins in order to better represent operating cost differences among coal plants. [↑](#footnote-ref-2)
